# Manifold-constrained plasticity enables stable learning in recurrent neural circuits

**DOI:** 10.1371/journal.pcbi.1014719

**Published:** 2026-08-25

**Authors:** Camille Godin, Jean-Philippe Thivierge

**Affiliations:** 1 School of Psychology, University of Ottawa, Ottawa, Canada; 2 Brain and Mind Research Institute, University of Ottawa, Ottawa, Canada; Swiss Federal Institute of Technology Zurich Institute of Neuroinformatics: Institut fur Neuroinformatik UZH/ETH, SWITZERLAND

## Abstract

The activity of large neuronal populations is often confined to low-dimensional manifolds that can drift over time, posing a challenge for learning rules that assume stable, full-rank representations. Here, we introduce SPLiT (Synaptic Projection Learning with intrinsic Tracking), a synaptic plasticity rule for recurrent neural networks that combines unsupervised manifold tracking with supervised learning. SPLiT uses an online Oja rule to continuously estimate the intrinsic low-dimensional activity subspace and performs normalized least-mean-squares learning in manifold coordinates. In this way, SPLiT keeps synaptic weights aligned with evolving population dynamics. Using a rate-based recurrent network, we show that SPLiT reliably learns time-varying target signals under both constrained dynamics, where activity is restricted to a fixed low-dimensional manifold, and unconstrained dynamics exhibiting changes in the dominant activity subspace. We show that manifold-constrained learning with SPLiT yields faster convergence, less sensitivity to recurrent gain, smaller weight updates, and is more robust to noisy teaching signals. Analytical results show that SPLiT learns the optimal decoder projected onto the instantaneous principal subspace and maintains bounded error under drift. Together, these findings provide a mechanistic account of how synaptic plasticity can leverage the structure of neural manifolds to enable stable and efficient supervised learning despite representational drift.

## Author summary

Neural activity in the brain can often be described by a small number of coordinated patterns that are far fewer than the total number of active neurons. These patterns, known as neural “manifolds,” can gradually change over time. This creates a fundamental challenge for learning: how can downstream circuits maintain stable behavior if the internal representations they depend on are drifting? In this work, we introduce a learning rule designed to address this problem by continuously tracking the dominant patterns of population activity and restricting learning to those patterns. Our model combines two complementary components: an unsupervised mechanism that estimates the current low-dimensional structure of neural activity, and a supervised mechanism that adjusts output connections to minimize task error within that structure. We show that this approach enables stable and efficient learning even as internal representations evolve. Across several tasks, including periodic signals, handwritten digit trajectories, and chaotic dynamics, our method outperforms existing learning rules when activity is confined to low-dimensional subspaces. It further predicts that learning within an intrinsic manifold should be faster and require smaller synaptic adjustments than learning that depends on activity outside that manifold. Together, these results suggest that tracking low-dimensional population structure provides a general strategy that allows neural circuits to function reliably despite ongoing representational drift.

## Introduction

Neural computation is thought to emerge from the coordinated activity of large populations of neurons [1–3]. Although the space of possible firing patterns is high-dimensional, neural activity in many brain areas is strikingly low-dimensional, with most variance confined to a limited set of prominent covariation patterns [4–9]. These patterns span a subspace, called a *neural manifold*, that captures the dominant structure of population activity [6,10–14].

**Fig 1:**
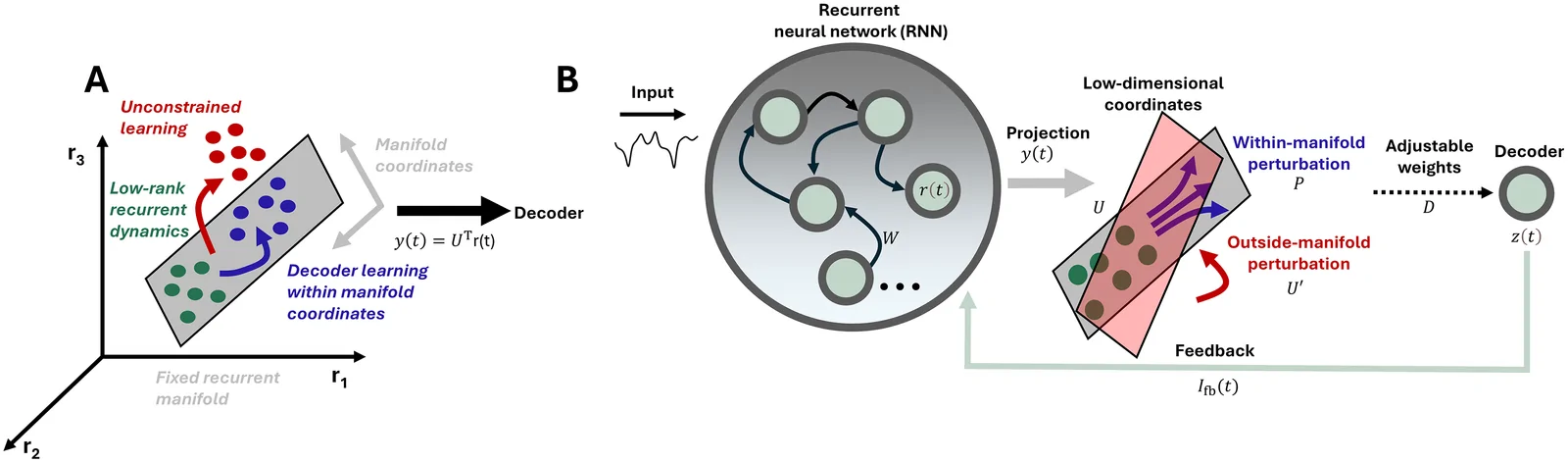
Constraining recurrent neural activity to a low-dimensional manifold. **(A)** Illustration of how the activity of three neurons (r_1_, r_2_, r_3_) at discrete time points (filled circles) can be constrained to a fixed manifold (grey rectangle) or explore an unconstrained space outside of the manifold. Grey arrows indicate the principal manifold coordinates, representing the dominant orthogonal directions that span the low-dimensional neural activity subspace within the higher-dimensional state space. Recurrent activity is projected to manifold coordinates via y(t), which then enters a linear decoder. **(B)** Architecture of a network where a time-dependent input activates recurrent units that send their output to a decoder. Perturbations are applied at the decoder. Within-manifold perturbations P remap latent coordinates y(t) within the estimated activity subspace U(t), whereas outside-manifold perturbations replace U(t) with a perturbed decoder basis $U^{\prime }$ rotated toward directions orthogonal to the manifold M. Decoding is performed through adjustable weights (D). Feedback from the decoder ($I_{\text{fb}})$ projects back to the recurrent network.

While manifolds were traditionally considered stable over time [15,16], recent work suggests that they can evolve dynamically [11,17–22]. Neural circuits can reorganize over time, even on short timescales, with implications for learning and memory. Specifically, population codes may change in two different ways: (*i*) changes constrained to lie within a fixed manifold, where firing rates drift along existing co-variation patterns [23], and (*ii*) unconstrained changes, where the correlation structure itself is altered over time [24] (Fig 1A). Both types of changes have been observed in brain-computer interface experiments, where monkeys learn new motor responses through either rapid changes constrained to a given manifold or slower, unconstrained changes outside of the manifold [22].

These observations raise the question of how neural circuits maintain intact function when their internal representations shift over time [25–32]. One possibility is that drift is confined to non-coding dimensions. Some theoretical models support this idea by showing that an accurate readout can be maintained when drift occurs in dimensions orthogonal to the coding space (so-called *null-space drift*) [27,33–35]. However, experimental and modeling studies increasingly report representational drift in both null and coding dimensions, challenging the idea that readouts remain static [26,36–40]**.**

An alternative possibility is that downstream readouts continually adapt to drifting neural representations. This adaptation may be achieved through mechanisms of synaptic plasticity that compensate for changes in population dynamics caused by drifting manifolds [41]. Studies in mammalian cortex reveal that synaptic connectivity is highly plastic, with a significant turnover on timescales ranging from hours to days [42–45]. This continual remodeling may allow readouts to adapt to ongoing changes in population activity [19,36].

Recurrent neural networks (RNNs) offer a powerful framework for modeling the complex temporal computations of neural systems [16,46–48]. Training algorithms such as FORCE can learn biologically realistic dynamics and perform behaviorally-relevant tasks [49–53]. However, they are limited in their ability to accommodate drifting manifolds, particularly when these manifolds represent low-dimensional fluctuations in neural activity. Supervised approaches that assume full-rank population representations may systematically fail in regimes where neural activity is intrinsically low-dimensional, which is a hallmark of cortical population recordings.

Here, we introduce a new learning rule termed SPLiT (Synaptic Projection Learning with intrinsic Tracking) that integrates unsupervised learning of low-dimensional manifolds with a supervised least mean squares (LMS) update. Specifically, SPLiT learning combines an online Oja rule [54] with a supervised decoder trained via error feedback. By dynamically tracking changes in the underlying neural manifold and incorporating this information into the learning process, SPLiT enables RNNs to adapt when internal representations evolve over time.

We demonstrate that SPLiT supports learning in networks with dynamically evolving manifolds by coupling online manifold tracking to supervised decoder adaptation. Unlike approaches that compensate for drift through readout plasticity alone [26,31,40,55], SPLiT continuously estimates the empirical low-dimensional activity subspace and restricts learning to manifold coordinates. This allows decoder weights to remain aligned with evolving population dynamics while suppressing unstable high-dimensional activity modes. Using a rate-based recurrent network, we show that SPLiT reliably learns time-varying target signals under both constrained and unconstrained dynamics, yielding faster convergence, smaller weight updates, and greater robustness to noisy teaching signals than full-rank learning rules. Analytical results demonstrate that SPLiT learns the optimal decoder projected onto the instantaneous principal subspace and maintains bounded error under drift. These results show that online tracking of low-dimensional population activity constitutes a computational mechanism for stable supervised learning under representational drift.

Below, we begin by describing a recurrent model that constrains RNN dynamics to low-dimensional manifolds. Then, we illustrate the performance of SPLiT learning on tasks that require tracking of time-evolving signals. Comparisons with FORCE learning and related approaches are provided along with a formal analysis of the computational advantages of SPLiT relative to LMS approaches.

## Results

### A learning rule that combines online manifold tracking with supervised decoder adaptation

We designed a randomly connected RNN that received external input in the form of a time-evolving signal (Fig 1B). Neurons within the network produced activity in response to this input as well as through their interconnections. This activity was projected to output units via adjustable connections that were trained to produce a target signal. The architecture of the model followed the framework of reservoir computing, with two key exceptions. First, neural activity within the RNN was controlled to generate either low-dimensional dynamics that were constrained to a fixed manifold, or high-dimensional dynamics that were unconstrained. Second, a new learning rule (SPLiT) was introduced to train output connections to track changes in latent dynamics.

Activity within the RNN evolved according to a standard mean-rate model (see Table 1 for default parameters),

**Table 1:** Default model parameters.

Parameter	Symbol	Default value
Time constant	τ	0.3
Number of recurrent units	N	100 (2 Hz target) 500 (MNIST targets)
Drift noise	$\eta_{r}$	0.001
Gain	g	1.5
Manifold dimension	k	3
Time step	Δt	0.01
Feedback strength	α	1.5
Oja manifold adaptation rate	$\eta_{U}$	0.01
Decoder learning rate	$\eta_{D}$	0.1


$$\begin{array}{c} \tau \dot{r}(t)=-r(t)+\text{tanh}\left(Wr(t)+I(t)+I_{\text{fb }}(t)+\eta_{r}\xi (t)\right), \end{array}$$ (1)

where τ is a time constant, tanh(·) is applied elementwise, $W\in R^{N\times N}$ is the recurrent connectivity matrix for N neurons, I(t) is an external input, $I_{\text{fb }}(t)$ is learning feedback (described below) and $\xi (t)\sim N\left(\text{0},I_{N}\right)$ is independent identically distributed zero-mean Gaussian noise with standard deviation $\eta_{r}$.

In the constrained dynamics condition, neural activity remains confined to a d-dimensional subspace of $R^{N}$. This condition is an idealized regime that could emerge biologically from network connectivity and stabilization mechanisms rather than an explicit projection as implemented here. This fixed projection serves as a controlled manipulation to isolate the effect of manifold alignment.

To restrict recurrent dynamics to a low-dimensional manifold, we defined an orthonormal basis


$$\begin{array}{c} Q\in R^{N\times k}, \end{array}$$ (2)

with $Q^{\text{T}}Q=I_{k}$, initialized randomly and held constant throughout learning. We refer to the “manifold” M=span(Q) as a fixed linear subspace. Low-dimensional coordinates are denoted as $x(t)\in R^{k}$,


$$\begin{array}{c} x(t)=Q^{\text{T}}r(t). \end{array}$$ (3)

The recurrent weight matrix $W_{k}\in R^{k\times k}$ was initialized by $W_{k}\sim N(\text{0},g^{\text{2}}/N)$ with recurrent gain g. The full N×N connectivity was then constructed as


$$\begin{array}{c} W=QW_{k}Q^{\text{T}}. \end{array}$$ (4)

When W is rank-k, all outside-manifold components decay exponentially. Because activity confined to M evolves under $W_{k}$, the stability of the within-manifold dynamics is directly determined by $\rho^{*}$. This procedure is similar to low-rank RNN connectivity in related work [56] except that the basis vectors are not designed to encode task-specific modes. Instead, they reflect random orthonormal directions.

For comparison, we considered an “unconstrained” version of the network without a restricted manifold. The firing-rate dynamics followed Eq. 1, but the recurrent weight matrix W was left unprojected. As before, weights were initialized from $N\left(\text{0},g^{\text{2}}/N\right)$. In this case, population activity evolved in the full N-dimensional space without restrictions.

Network outputs were generated through a trainable decoder that mapped recurrent activity to a target signal. Decoder weights were updated using a new synaptic rule termed SPLiT. As in standard supervised learning, the objective of this rule is to minimize the difference between the network output and a target. However, SPLiT augments decoder updates with an online estimate of the activity manifold, allowing the readout weights to adapt in a way that remains aligned with the evolving subspace of the RNN. SPLiT works by first tracking the activity manifold using an unsupervised Oja rule. Then, it applies LMS supervised learning in manifold space. The manifold-tracking component of SPLiT can be interpreted as a population-level mechanism that stabilizes dominant modes of neural variability.

To track the activity manifold, SPLiT begins by computing the scalar population mean activity,


$$\begin{array}{c} \mu (t)=\frac{\text{1}}{N}\sum_{j=\text{1}}^{N}r_{j}(t), \end{array}$$ (5)

and centered activity as


$$\begin{array}{c} \tilde{r}(t)=r(t)-\mu (t)1, \end{array}$$ (6)

where $1\in R^{N}$ is the all-ones vector. An estimate of the k-dimensional activity manifold is represented by an orthonormal basis $U(t)\in R^{N\times k}$ whose columns $u_{i}(t)$ span the dominant directions of variability. This basis is updated online using Oja’s rule,


$$\begin{array}{c} \dot{U}=\eta_{U}\left[\tilde{r}(t)\tilde{r}(t{)}^{\text{T}}U-U\left(U^{\text{T}}\tilde{r}(t)\tilde{r}(t{)}^{\text{T}}U\right)\right], \end{array}$$ (7)

where $\eta_{U}$ is a learning rate, and


$$\begin{array}{c} y(t)=U^{\text{T}}(t)\tilde{r}(t) \end{array}$$ (8)

represents the projection of the full population activity into manifold coordinates. This rule tracks slow changes in the subspace generated by the recurrent dynamics. More precisely, the rule tracks the empirical activity subspace U, not the imposed structural subspace Q. Oja’s rule converges when inputs change slowly compared to the update rate of the manifold, the learning rate $\eta_{U}$ is sufficiently small, and the data distribution is approximately stationary over the update window (see S1 Fig). The subspace U(t) is re-orthonormalized at every time step (1 ms) to maintain numerical stability.

The manifold coordinates y(t) should not be interpreted as requiring a distinct neural population that represents a latent state separate from the recurrent activity r(t). Rather, y(t) corresponds to dominant covariance patterns embedded within the population activity. Thus, the projection in Eq. 8 provides a description of ongoing activity rather than a separate dynamical variable.

The network output was computed as a linear readout of the manifold,


$$\begin{array}{c} z(t)=D(t)y(t), \end{array}$$ (9)

where $D(t)\in R^{m\times k}$ is a decoder mapping the k-dimensional manifold into an m-dimensional output. Each column of D corresponds to a readout applied to one dimension of the learned manifold. Thus, output weights were updated by mapping low-dimensional features y(t) to a desired output z(t). This differs from standard LMS, where the decoder acts on the full activity r(t). In SPLiT, learning is restricted to the manifold tracked by U(t).

Given a target signal $z^{*}(t)\in R^{m}$, the cost function was defined as


$$\begin{array}{c} L(t)=\frac{\text{1}}{\text{2}}{\left\|z(t)-z^{*}(t)\right\|}^{\text{2}}, \end{array}$$ (10)

with instantaneous error


$$\begin{array}{c} e(t)=z(t)-z^{*}(t). \end{array}$$ (11)

SPLiT updates the decoder D(t) using a normalized LMS rule in manifold coordinates,


$$\begin{array}{c} \dot{D}(t)=-\eta_{D}e(t)\frac{y(t{)}^{\text{T}}}{{\left\|y(t)\right\|}^{\text{2}}+\epsilon }, \end{array}$$ (12)

with


$$\begin{array}{c} {\left\|y(t)\right\|}^{\text{2}}=\sum_{i=\text{1}}^{k}y_{i}^{\text{2}}(t), \end{array}$$ (13)

where $\eta_{D}$ is the learning rate of the decoder and ϵ is a small positive constant.

For the constrained network, the learning feedback $I_{\text{fb}}(t)$ from the learning rule to the RNN (Eq. 1) is given by


$$\begin{array}{c} I_{\text{fb}}(t)=\alpha QQ^{\text{T}}U(t)D(t{)}^{\text{T}}z^{*}(t), \end{array}$$ (14)

where α controls the feedback amplitude. In the unconstrained network, the projection $QQ^{\text{T}}$ is omitted. The feedback term $I_{\text{fb}}$ encourages RNN activity to visit manifold directions relevant to the target output. This feedback should be interpreted as a teacher signal that shapes the states that are visited during learning. While this makes SPLiT similar to adaptive control during training [57], the learning goal remains the decoder mapping, and performance during autonomous replay (see Results) shows that the network does not simply rely on the external target to produce its output.

SPLiT is designed to track the empirical covariance structure of population activity rather than architectural constraints imposed on the network. Although simulations of constrained activity restrict recurrent dynamics to a fixed low-dimensional subspace (defined by Q), the manifold tracked by SPLiT is the activity-dependent subspace U(t) extracted from ongoing neural activity. Thus, the learning rule adapts to the intrinsic population activity patterns expressed by the network, whether they arise from constrained or unconstrained recurrent connectivity.

In the present framework, drift in the covariance structure arises from the interaction between decoder adaptation and recurrent dynamics. Specifically, changes in decoder weights D(t) alter the feedback input $I_{\text{fb}}(t)$, which in turn modifies the trajectories of recurrent activity explored by the network. As learning progresses, these trajectories reshape the covariance structure of the network. Thus, even though the recurrent connectivity matrix W remains fixed, the low-dimensional manifold can evolve over time. The stochastic noise term ξ(t) contributes additional variability to neural activity but it is not the primary source of manifold drift in the present simulations.

An important assumption of the proposed framework is a separation of timescales between changes in the population activity manifold and the plasticity mechanism that tracks it. Specifically, we assume that drift in the empirical low-dimensional structure of recurrent activity is sufficiently slow relative to the update of the unsupervised manifold-tracking rule (Eq. 7). Under this condition, Oja’s rule provides a valid estimate of the instantaneous principal subspace of population activity. This assumption is consistent with experimental observations showing that population-level representational drift typically unfolds over long timescales [22].

SPLiT is not intended as a biologically detailed implementation of the precise synaptic mechanisms by which neural circuits track ongoing changes in low-dimensional manifolds. While the Oja rule used to track the principal subspace takes the form of a Hebbian rule, the decoder update relies on an explicit error term and normalization, which requires nonlocal information. Thus, SPLiT should be viewed as an abstraction of underlying synaptic processes operating at the population level, which remain to be fully understood. As such, our goal is to examine whether the combination of subspace-tracking and supervised learning constitutes a plausible computational mechanism by which synaptic plasticity can support stable information processing in the presence of drifting population dynamics. Focusing on mechanistic principles rather than biophysical details allows us to investigate the role of learning mechanisms based on low-dimensional manifolds in solving tasks relevant to neural computation.

### Manifold-constrained learning stabilizes decoder adaptation in low-dimensional networks

**Fig 2:**
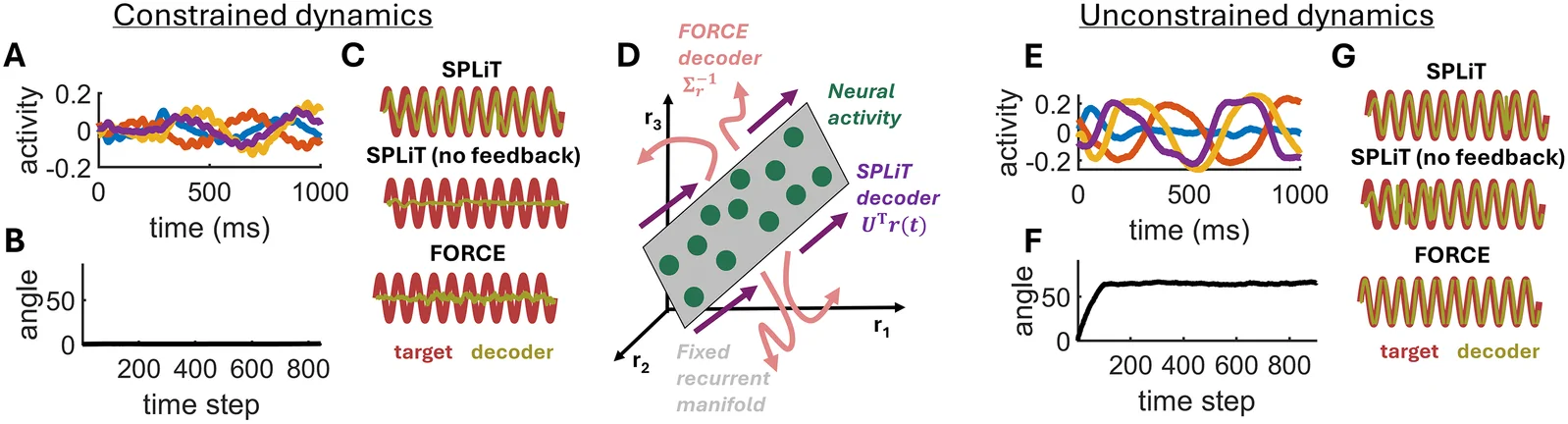
Learning to track an oscillation with SPLiT using recurrent dynamics that are constrained to a fixed manifold. **(A)** Example of neural activity from four recurrent neurons (shown by distinct colors). **(B)** The mean principal angle (Methods Eq. 37) was close to zero, showing that activity remained confined to the manifold. **(C)** A decoder trained by SPLiT learning learned to track the target signal, while FORCE learning and SPLiT without feedback ($I_{\text{fb}}$) did not. **(D)** Schematic comparing SPLiT and FORCE decoders applied to a low-dimensional manifold. Colored arrows show that SPLiT decoder directions are aligned with the activity subspace while FORCE directions are not aligned. Panels **(E-G)** are obtained from a recurrent network that was not constrained to a fixed manifold.

As a simple starting point, we considered a task where the RNN tracked a target signal that consisted of a 2 Hz sine wave (more complex signals are considered below). The network readout was trained using SPLiT to approximate this signal at the output of the RNN. Before examining the performance of SPLiT on this task, we verified that in a scenario where an RNN was designed with constrained activity (Eqs. 1–4), dynamics remained confined to a fixed manifold. This was quantified by computing the mean principal angle (Methods, Eqs. 35–37) between the initial manifold and the instantaneous activity subspace over time. Under constrained dynamics, this angle remained close to zero (Fig 2A-2B), indicating strong alignment between the two subspaces. Thus, constrained RNN activity remained within a fixed manifold determined at the onset of training.

When operating within the constrained activity regime, SPLiT successfully approximated the target signal (Fig 2C), whereas FORCE performed poorly. Intuitively, FORCE assumes that neural activity spans a sufficiently rich high-dimensional space such that small changes in decoder weights can reconstruct the target signal. However, when activity is restricted to a low-dimensional manifold, many directions in neural space become unavailable, causing the covariance structure of neural activity to become low-rank. In this regime, FORCE becomes ill-conditioned because the decoder attempts to invert activity patterns that no longer contain independent dimensions. This instability arises because FORCE distributes decoder updates across directions that are poorly aligned with the intrinsic manifold. In contrast, SPLiT continuously aligns decoder updates with the dominant low-dimensional activity subspace (Fig 2D). Although FORCE could in principle be modified through a pseudoinverse formulation, or by rewriting the learning rule in intrinsic manifold coordinates, such approaches assume prior access to the manifold structure itself. In contrast, SPLiT provides a synaptic mechanism that continuously estimates and tracks the evolving activity subspace during learning, thereby constraining decoder adaptation to directions supported by the intrinsic population dynamics.

Results obtained from a constrained RNN were compared to an unconstrained scenario, where recurrent dynamics were free to explore the full N-dimensional activity space. In this regime, activity no longer remained confined to a stable low-dimensional subspace, yielding large principal-angle deviations relative to the initial activity subspace (Fig 2E-2F). These deviations reflect the emergence of additional high-dimensional activity modes rather than a single rotating manifold. When network dynamics were unconstrained, both SPLiT learning and FORCE accurately tracked the sine wave (Fig 2G). In SPLiT, removing the feedback term ($I_{\text{fb}}$) (Eq. 1) disrupted performance under constrained dynamics but not when dynamics were left unconstrained (Fig 2C vs. 2G).

### Decoder performance depends on alignment with the intrinsic activity manifold

**Fig 3:**
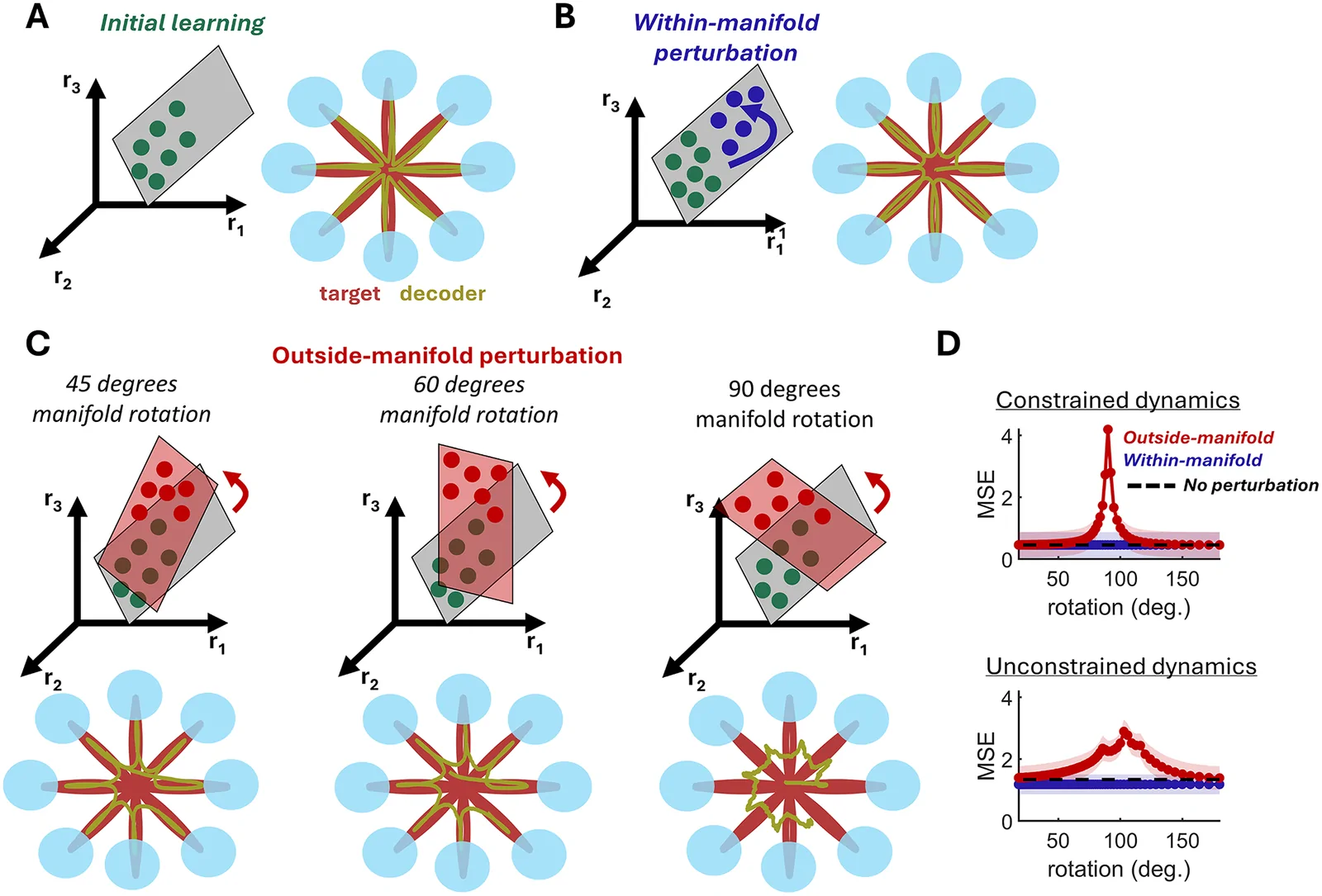
Impact of manifold perturbations on learning a two-dimensional center-out reaching task. **(A)** During the initial learning stage, RNN activity evolved according to a low-dimensional manifold and the decoder was trained to follow the radial targets. Blue circles represent putative reach targets. **(B)** After training, a within-manifold perturbation was applied by rotating activity within the manifold coordinates y(t), which did not disrupt performance. **(C)** An outside-manifold perturbation was applied to rotate activity away from the manifold M, which disrupted performance. Simulations were run using unconstrained dynamics. **(D)** MSE was higher for outside-manifold than within-manifold perturbations for both constrained and unconstrained dynamics. Shaded area is standard error of the mean across 10 independent realizations.

To examine the impact of an abrupt remapping of the decoder, we first trained the RNN to perform a two-dimensional center-out reaching task consisting of eight radially arranged targets around a central origin (Fig 3A) [58]. The decoder was trained using SPLiT while the recurrent dynamics evolved either under constrained or unconstrained activity regimes. After this initial learning phase, we introduced an abrupt perturbation at the decoding stage while leaving both the recurrent connectivity matrix W and the manifold Q unchanged (Fig 1B).

Two types of perturbations were examined. In a within-manifold perturbation, latent coordinates were remapped through an orthogonal rotation within the intrinsic activity subspace,


$$\begin{array}{c} y_{\text{rotated}}(t)=Py(t),\;P^{\text{T}}P=I, \end{array}$$ (15)

where $P\in R^{k\times k}$ is an orthonormal random rotation matrix obtained by QR decomposition. This perturbation preserves the underlying manifold while rotating the coordinate representation within that subspace. In an outside-manifold perturbation, the estimated decoder basis itself was rotated toward directions orthogonal to the manifold M,


$$\begin{array}{c} U^{\prime }=\text{orth}\left(\text{cos}(\theta )U+\text{sin}(\theta )U_{⊥}\right), \end{array}$$ (16)

where $U_{⊥}$ satisfies $Q^{\text{T}}U_{⊥}=\text{0}$ and θ controls the degree of outside-manifold rotation. Both within- and outside-manifold perturbations involved rotational changes in manifold structure rather than additive translations or shifts in neural activity. Trials lasted 10 s, with perturbations introduced half-way through each trial. Following perturbations, decoder learning was suspended for 1.6 s while recurrent activity and feedback dynamics continued to evolve. Decoder adaptation was then reactivated and performance was evaluated during the subsequent period.

For within-manifold perturbations, task performance was largely preserved (Fig 3B), indicating that the learned solution remained accessible within the same manifold. In contrast, outside-manifold perturbations produced a performance degradation (Fig 3C). This disruption increased systematically with the rotation angle: modest error was observed at intermediate rotations (e.g., 45°), whereas orthogonal rotations (90°) led to higher error (Fig 3D). With outside-manifold perturbations, error increased with the rotation angle θ because the decoder basis was rotated toward directions orthogonal to the manifold M, thereby reducing the alignment between decoder coordinates and the dominant recurrent activity subspace.

As an additional test, we initialized the decoder basis U outside of the subspace M. The mean principal angle between U and M was initially high but declined rapidly over training (S2 Fig), indicating that SPLiT continuously drives the decoder basis toward the dominant intrinsic activity subspace generated by the network.

In sum, these results demonstrate that task performance depends on the alignment between the decoder and the activity manifold. Perturbations confined to the learned subspace preserve performance, whereas rotations that drive the readout outside that manifold disrupt learning in proportion to the degree of misalignment. In constrained networks, perturbations produced only minor changes in the dominant recurrent activity subspace because the recurrent dynamics remained restricted to the fixed low-dimensional manifold. Thus, performance degradation primarily reflected a mismatch between decoder and recurrent activity rather than a large-scale reorganization of recurrent population dynamics.

These findings are consistent with experiments in motor cortex where animals rapidly adapted their behavior when neural activity was restricted to a pre-existing low-dimensional manifold, whereas learning that required exploration outside the intrinsic manifold unfolded over multiple training sessions spanning days [22]. Our computational framework recapitulates the mechanistic distinction identified experimentally: fast adaptation can proceed through reweighting existing neural population patterns, whereas larger rotations that require activity outside the intrinsic manifold impose a greater burden on learning.

### SPLiT supports decoding of complex trajectories

**Fig 4:**
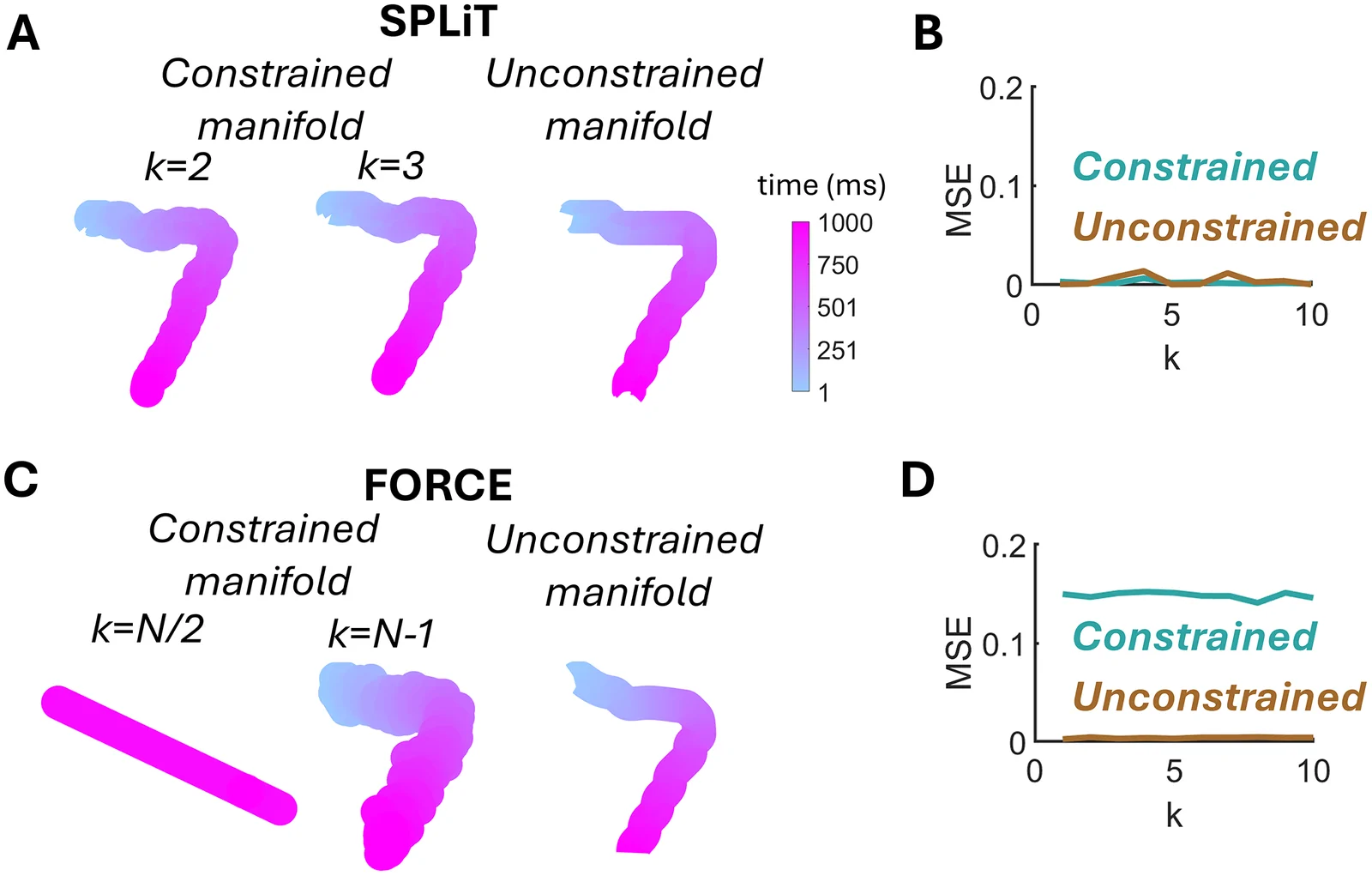
Tracing MNIST digits using constrained and unconstrained manifolds. **(A)** Examples of traced digits using SPLiT learning with various manifold dimensionality (k). **(B)** SPLiT yielded low tracking mean squared error (MSE) across values of k. **(C-D)** FORCE learning yielded high error when dynamics were constrained to a fixed low-dimensional manifold and lower error when dynamics were unconstrained.

Moving to a more elaborate task, we examined the ability of an RNN to trace two-dimensional handwritten digits from the MNIST dataset. Individual MNIST digits were first reduced to a one-pixel-wide representation, preserving their overall shape and topology. The resulting coordinates were then converted to a continuous two-dimensional trajectory of 1,000 data points that evolved over time (one point per time step). The decoder was trained using SPLiT to reproduce these trajectories. A total of 1,000 randomly chosen digits were employed on networks with randomized initial conditions. One data point was presented for each time step (1 ms). SPLiT learning can accurately trace digits with both constrained and unconstrained recurrent dynamics (Fig 4A-4B). Simulations examining the impact of parameters including manifold adaptation rate ($\eta_{U}$) and number of recurrent neurons (N) are shown in S1 Fig. FORCE learning, however, performed poorly when dynamics were constrained to a fixed manifold, even when the dimensions of the manifold were close to the full network (N−1) (Fig 4C-4D). Thus, SPLiT learning tracked ongoing changes in population activity within a given fixed manifold and employed this activity to learn a temporally evolving signal.

Because FORCE learning relies on recursive least-squares updates in the full N-dimensional neural space, it becomes ill-conditioned when the covariance matrix is close to singular. Even removing a small number of degrees of freedom can destabilize inversion-based learning updates. By contrast, SPLiT learns within the intrinsic subspace, avoiding the need to invert poorly conditioned directions outside the manifold. The MNIST task makes this effect prominent because accurate digit tracing requires stable decoder updates over many time steps.

### Manifold-constrained dynamics reduce sensitivity to recurrent gain

**Fig 5:**
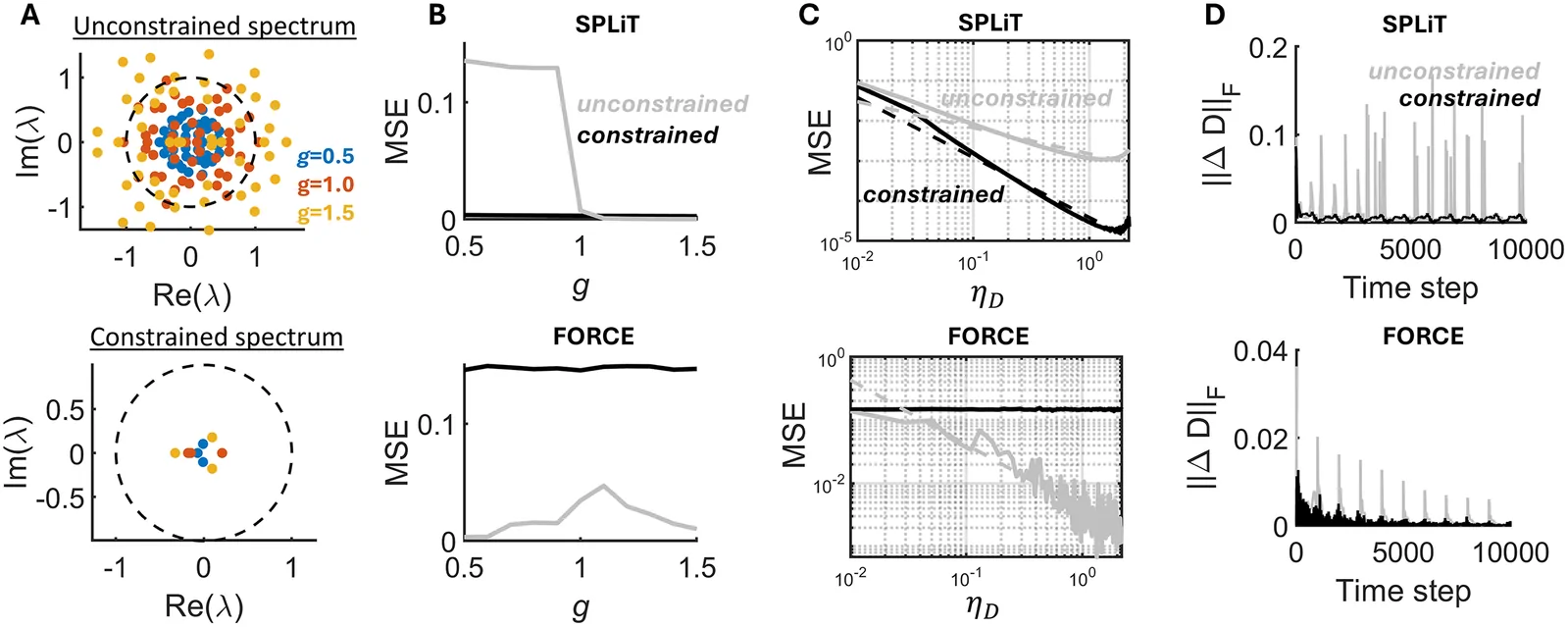
Impact of recurrent gain (g) and decoder learning rate ($\eta_{D}$) on SPLiT learning. **(A)** Eigenvalue spectrum of recurrent activity. The recurrent gain (g) impacts the distribution of eigenvalues when recurrent dynamics are unconstrained but not when they are constrained. Dashed line: unit circle. **(B)** Mean squared error (MSE) is sensitive to network gain when activity is not constrained to a fixed manifold. **(C)** With constrained dynamics, error decreases steeply as the decoder learning rate increases. Dashed lines: best-fitting power laws. **(D)** The magnitude of decoder weight modifications (${\left\|\Delta D\right\|}_{F}$) is higher when dynamics are unconstrained.

In follow-up simulations using the MNIST task, we explored the role of the recurrent gain parameter (g), which controls chaotic dynamics. Specifically, recurrent activity enters a chaotic regime when g>1 [59]. In unconstrained networks, increasing g broadens the eigenvalue distribution of the recurrent connectivity matrix and increases the spectral radius of the recurrent dynamics (Fig 5A, top). By contrast, in the constrained regime, recurrent activity is projected to a fixed low-dimensional manifold, suppressing many of the high-dimensional modes that would otherwise emerge as g increases. As a result, constrained dynamics are less sensitive to g (Fig 5A, bottom).

When SPLiT was trained with unconstrained dynamics, error decreased rapidly when g>1, showing that learning was sensitive to the network state (Fig 5B). In comparison, constrained dynamics yielded low error regardless of the value of g (ranging from 0.5 to 1.5). In these networks, activity is forced to evolve strictly within a low-dimensional manifold, which drastically diminishes the dependence on g. Put differently, compressing the RNN down to k dimensions (Eqs. 2–3) eliminates much of the structure in W that gets altered by g, and particularly the high-dimensional modes that become unstable as g increases. In contrast, in unconstrained networks, increasing g expands the spectral radius of the recurrent matrix, which drives the system toward high-dimensional activity. In turn, this increases the expressive capacity of the reservoir and allows the decoder to better represent the target signal.

Under constrained dynamics, recurrent activity evolves within a rank-k subspace. Because decoder learning depends only on activity projected into this low-dimensional subspace, increasing g cannot recruit additional high-dimensional modes. In contrast, unconstrained networks allow gain-dependent amplification across the full recurrent spectrum, producing increasingly high-dimensional activity as g increases.

Because the target signal enters the recurrent dynamics through the feedback term $I_{\text{fb}}(t)$ (Eq. 1), noisy targets can perturb the network not only through decoder error but also by injecting fluctuations directly into recurrent activity. To quantify this effect, we measured the mean trajectory deviation


$$\begin{array}{c} \Delta r=\frac{\text{1}}{T}\sum_{t=\text{1}}^{T}\left\|r_{\text{noisy}}(t)-r_{\text{clean}}(t)\right\|, \end{array}$$ (17)

while varying feedback strength α. Using frozen initial conditions, we compared recurrent activity $r_{\text{noisy}}(t)$ generated when the target signal contained additive noise (zero-mean, unit variance) against activity $r_{\text{clean}}(t)$ generated with a noise-free target. Increasing feedback strength produced progressively larger Δr, suggesting a trade-off between stronger feedback improving learning while at the same time allowing target noise to disrupt recurrent dynamics (S3 Fig). Perturbations induced by noisy feedback remained smaller than the variability Δr observed across different initial conditions with a noise-free target. Hence, feedback-driven perturbations produced smaller alterations in recurrent activity than the variability induced by differences in initial conditions alone.

In the absence of feedback (α=0), constrained networks exhibited high error that remained largely insensitive to g, indicating that constrained manifolds alone did not lead to accurate decoding (S4A Fig). Increasing feedback strength (α>0) reduced error across all values of g. Across values of α, decoder performance remained insensitive to recurrent gain, suggesting that manifold-constrained dynamics suppress instabilities due to recurrent gain. The relative drive of feedback and recurrent activity was quantified using the mean ratio


$$\begin{array}{c} R_{\text{fb}}=\frac{\text{1}}{T}\sum_{t=\text{1}}^{T}\frac{\left\|I_{\text{fb}}(t)\right\|}{\left\|Wr(t)\right\|}. \end{array}$$ (18)

Across values of α>0 and g between 0.5 and 1.5, $R_{\text{fb}}$ remained above one, indicating that feedback input was larger than recurrent input, and hence played a dominant role in shaping the instantaneous recurrent dynamics during learning.

### Manifold-constrained learning improves optimization and reduces synaptic modification

A series of simulations examined the impact of the decoder learning rate ($\eta_{D}$). In the constrained case, error decreased rapidly as $\eta_{D}$ increased (Fig 5C), whereas the unconstrained case exhibited a shallower improvement. Power-law fits revealed that error scaled approximately as MSE~$\eta_{D}^{-\beta }$, with a larger exponent for the constrained network (β=1.498, R^2^ = 0.955) than the unconstrained network (β= 0.653, R^2^ = 0.819). Thus, manifold-constrained learning not only approached optimal solutions more rapidly as $\eta_{D}$ increased, but also attained lower error rates.

These results suggest that constraining recurrent dynamics to a stable low-dimensional manifold improves decoder optimization. In the constrained regime, learning occurs within a compact and well-conditioned feature space, allowing larger learning rates to accelerate convergence without destabilizing the decoder. In contrast, unconstrained dynamics generate high-dimensional activity patterns that produce poorer conditioning and higher residual error.

Recent theoretical work has shown that learning performance depends on the conditioning of the optimization landscape [59]. In particular, Raman et al. [60] showed that learning speed is constrained by the learning rate and the shape of the error surface. Our results are consistent with this framework. By constraining recurrent activity to a low-dimensional manifold, SPLiT reduces the dimensionality of the optimization problem and suppresses high-dimensional activity modes. This produces a more structured feature space, allowing larger decoder learning rates to accelerate convergence without destabilizing learning. In contrast, unconstrained recurrent dynamics generate high-dimensional activity patterns that slow error reduction.

Next, we compared the magnitude of decoder weight changes in constrained and unconstrained networks. We found that decoder weight updates were larger in unconstrained networks than constrained networks throughout training (Fig 5D). Unconstrained dynamics generate high-dimensional and continuously drifting activity patterns, forcing the decoder to make larger corrective adjustments to track the target output. In contrast, constraining activity to a stable low-dimensional manifold produces well-structured features, allowing accurate decoding to be achieved with relatively small weight modifications. This is relevant from a biological perspective given that large synaptic updates are metabolically costly and could have a destabilizing effect on neural dynamics.

As an additional control, simulations were performed where we equalized the variance of the in-manifold activity between constrained and unconstrained regimes. The performance of constrained networks persisted in this scenario (S5 Fig). Thus, the performance of constrained networks cannot be explained solely by a lower variance in activity. Rather, the computational advantage stems from improved optimization under constrained dynamics.

These results are consistent with experimental observations showing that learning constrained to a stable neural manifold occurs on faster timescales than unconstrained learning. In motor cortex recordings from monkeys, animals adapted their behavior within minutes to hours when neural activity was constrained to a pre-existing low-dimensional manifold [22]. However, learning that required exploration outside the intrinsic manifold unfolded over multiple training sessions spanning days.

Analogously, in our model, constrained dynamics yielded a well-conditioned feature space in which increasing the decoder learning rate led to rapid error reduction, whereas unconstrained dynamics exhibited slower improvements and higher residual error. With SPLiT learning, a longer timescale of unconstrained learning is suggested to emerge from the need to have large cumulative weight changes that compensate for unstable neural representations [61], thus providing a mechanistic link between manifold structure and learning efficiency. These findings suggest that manifold-constrained learning supports fast and efficient adaptation, while unconstrained learning is limited by the need to reorganize higher dimensional and less stable neural activity patterns.

### Manifold-constrained learning confers robustness to noisy teaching signals

Next, we hypothesized that an advantage of low-dimensional RNN activity is its robustness to noise injected in the target signal, given that constrained dynamics may prevent the network from tracking noisy components of the target. Technically, because SPLiT performs regression within the instantaneous principal subspace U(t), noisy target signals should have a reduced influence on the learned decoder compared to full-rank LMS.

**Fig 6:**
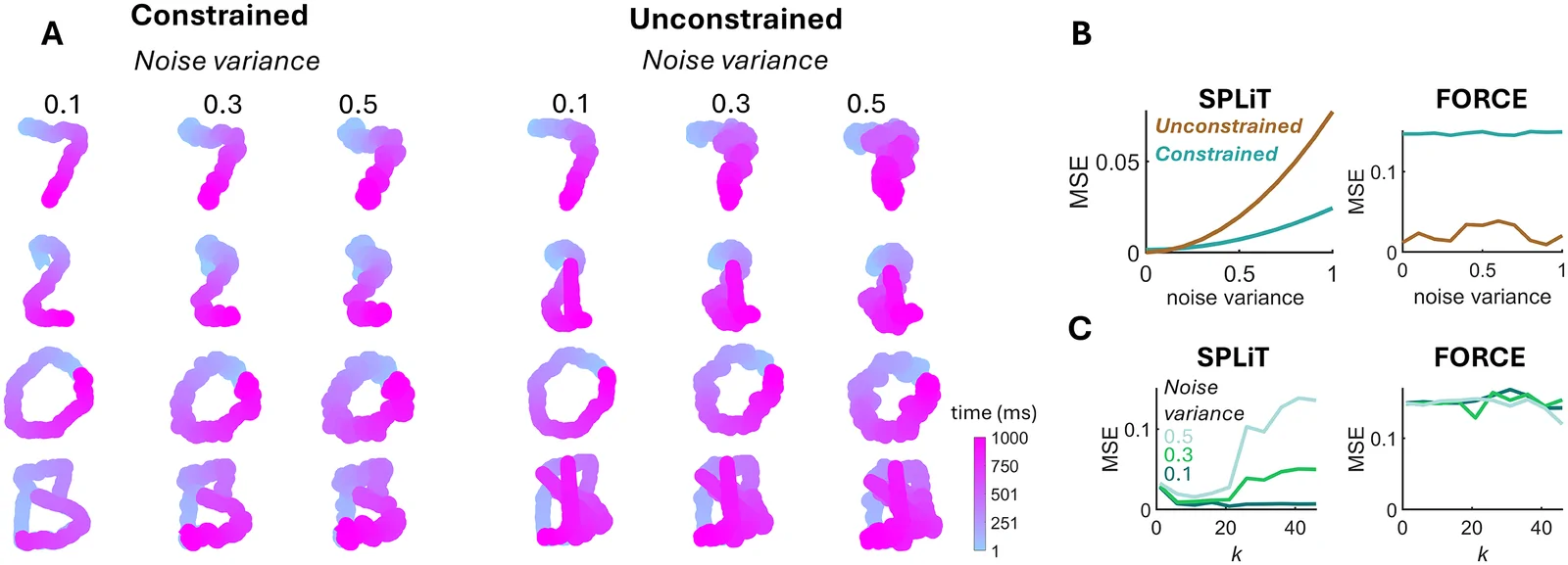
Impact of noise on SPLiT learning. **(A)** Examples of single MNIST digits injected with various levels of noise and trained with constrained vs. unconstrained networks. **(B)** When the target signal is injected with random noise, error (MSE) rises more rapidly with unconstrained than constrained (k=3) dynamics. **(C)** In constrained networks, higher dimensionality of the manifold (k) results in higher error. Panels B-C are means over 10 independent realizations with random initial seeds.

To test this idea, we added zero-mean Gaussian noise to MNIST handwritten digits. When the variance of the noise was low (i.e., with a magnitude of 0.1 or below), only a marginal difference in training error was found between constrained and unconstrained networks (Fig 6A, 6B). However, with increased noise variance, error in the unconstrained scenario rose sharply while the constrained scenario remained lower. Thus, constrained dynamics provide an advantage in robustness when learning noisy target signals.

To examine the impact of neural dimensionality (k) on performance, we performed simulations with constrained RNN dynamics where k was varied systematically. We found that as dimensionality increased, error became increasingly sensitive to noise in the target signal (Fig 6C). In this way, the highest error was found when both dimensionality and noise variance were high. These results further support the role of low-dimensional dynamics in dampening the impact of target noise on performance error.

To determine the contribution of feedback ($I_{\text{fb}}$) to robustness to noisy targets, simulations were performed where recurrent dynamics remained unconstrained while the feedback was restricted to a low-dimensional subspace. Lowering the rank of the feedback to 1 produced only minimal changes in decoder performance compared to rank k (S6 Fig). Thus, the improved robustness of manifold-constrained learning was largely independent of the feedback rank. Rather, robustness arises from constraining the recurrent dynamics themselves, which suppresses high-dimensional activity modes.

### Manifold-constrained dynamics improve the stability of autonomous replay

**Fig 7:**
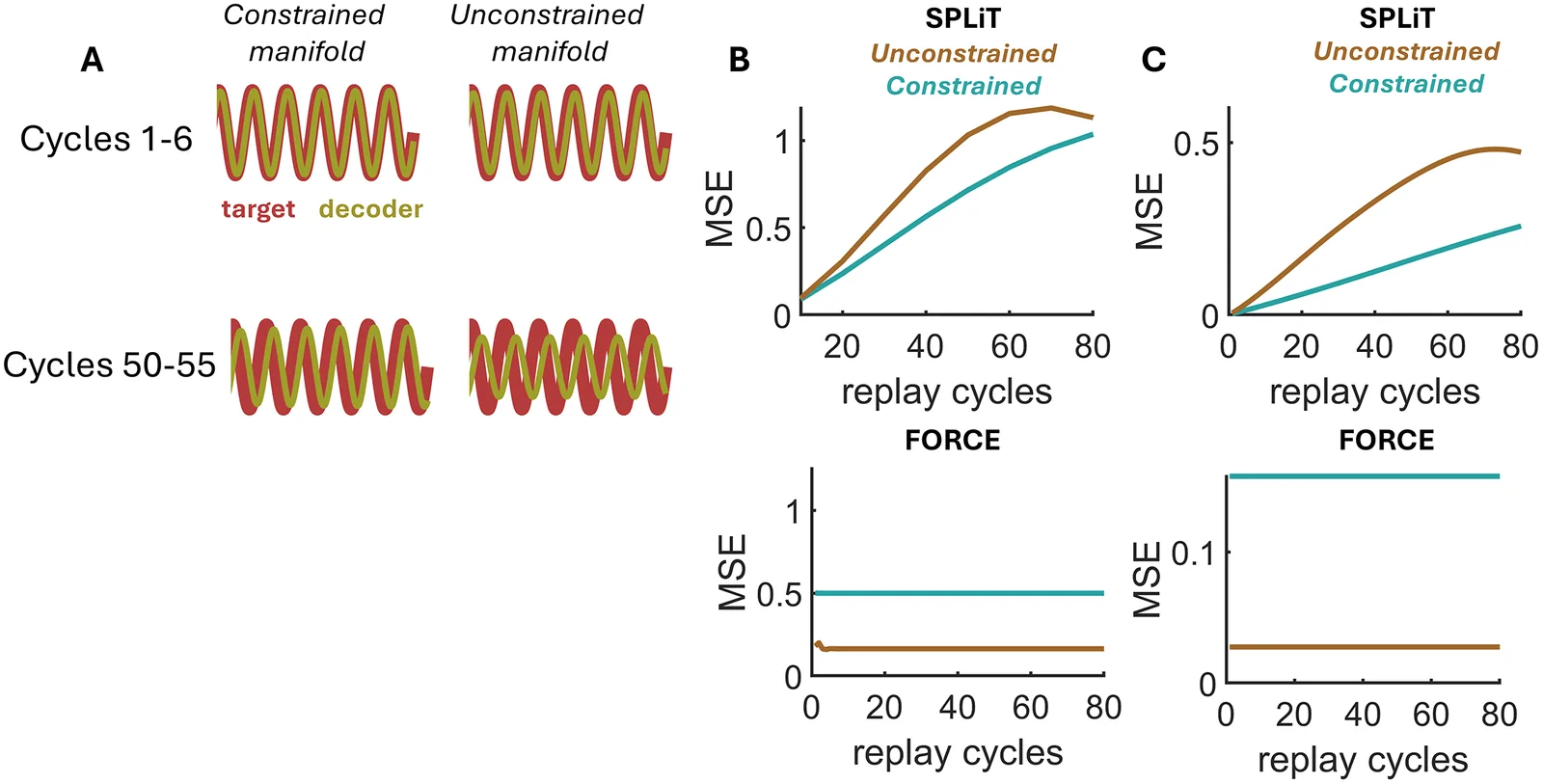
Autonomous replay of a temporal sequence. **(A)** Replay of a sine wave (2 Hz) after training. Performance with constrained vs. unconstrained recurrent activity is similar during the first few cycles of replay (cycles 1-6), but worse for unconstrained activity during later cycles (cycles 50-55). **(B)** Error is overall higher with unconstrained activity during replay of a 2 Hz sine wave. **(C)** Replaying MNIST digits yielded a similar result.

We examined the ability of recurrent networks to autonomously replay learned temporal sequences. Both a simpler (2 Hz sine wave) and more complex (MNIST) task were considered. First, we trained an RNN to track a 2 Hz sine wave lasting 10 seconds. Then, we froze the output weights of the RNN and fed the output of the network back to the RNN as input. The goal of the readout was to replay the learned sine wave pattern over as many cycles as possible. During the first few cycles, the network closely reproduced the target sine wave (Fig 7A). However, replay error increased gradually with further cycles. This degradation in performance occurred more slowly when activity was constrained to a fixed low-dimensional manifold than when activity evolved freely in the full high-dimensional state space (Fig 7B). Moreover, replay error was consistently higher for unconstrained activity than for constrained activity. Similar results were obtained when RNNs were trained to replay MNIST digits (Fig 7C). Together, these results show that constraining neural activity to a stable low-dimensional manifold enhanced the robustness of autonomous replay, therefore providing a computational advantage over unconstrained dynamics.

### SPLiT outperforms existing drift-adaptation rules under constrained dynamics

We compared SPLiT to six related approaches that were designed to address the challenge of representational drift. To enable direct comparisons, all approaches were trained on the same reservoir architecture and only the readout plasticity rule was altered. Three tasks were examined, namely a 2 Hz sine wave (for further oscillation frequencies, see S7 Fig), a Lorenz attractor, and a Rössler attractor. For all six related approaches, the decoder operated directly on the full activity $r(t)\in R^{N}$, in contrast to SPLiT which learns in manifold coordinates. For consistency, all models were implemented using the same reservoir architecture and task conditions. The scalar network output is given by


$$\begin{array}{c} z(t)=D(t)r(t), \end{array}$$ (19)

where, in contrast to SPLiT, the decoder D(t) acts on the full activity space rather than manifold coordinates.

First, we examined an ideal observer model (IO) [55]. In contrast to SPLiT, which updates only the readout weights, the ideal observer model uses a recursive least-squares update applied to the recurrent weights, after transforming the output error through a feedback matrix computed as the pseudo-inverse of the readout. The readout matrix D is fixed, and the output error


$$\begin{array}{c} e(t)=z(t)-z^{*}(t) \end{array}$$ (20)

is projected back into the network through a feedback matrix computed as the pseudoinverse of the readout. Each postsynaptic neuron i maintains an inverse correlation matrix


$$\begin{array}{c} \Gamma_{i}(t)\in R^{N\times N}. \end{array}$$ (21)

The recursive least-squares gain vector is


$$\begin{array}{c} g_{i}(t)=\frac{\Gamma_{i}(t)r(t)}{\text{1}+r(t{)}^{\text{T}}\Gamma_{i}(t)r(t)}. \end{array}$$ (22)

The incoming recurrent weights onto neuron i, denoted $w_{i}(t)$, are updated as


$$\begin{array}{c} {\dot{w}}_{i}(t)=-\eta_{\text{rec}}e_{i}(t)g_{i}(t), \end{array}$$ (23)

where $e_{i}(t)$ is the neuron-specific projected error and $\eta_{\text{rec}}=\text{0}.\text{5}$ is a scaling parameter. The inverse correlation estimate evolves according to


$$\begin{array}{c} {\dot{\Gamma }}_{i}(t)=-\frac{\Gamma_{i}(t)r(t)r(t{)}^{\text{T}}\Gamma_{i}(t)}{\text{1}+r(t{)}^{\text{T}}\Gamma_{i}(t)r(t)}. \end{array}$$ (24)

Unlike SPLiT, this approach does not track a drifting manifold and does not restrict learning to intrinsic coordinates.

**Fig 8:**
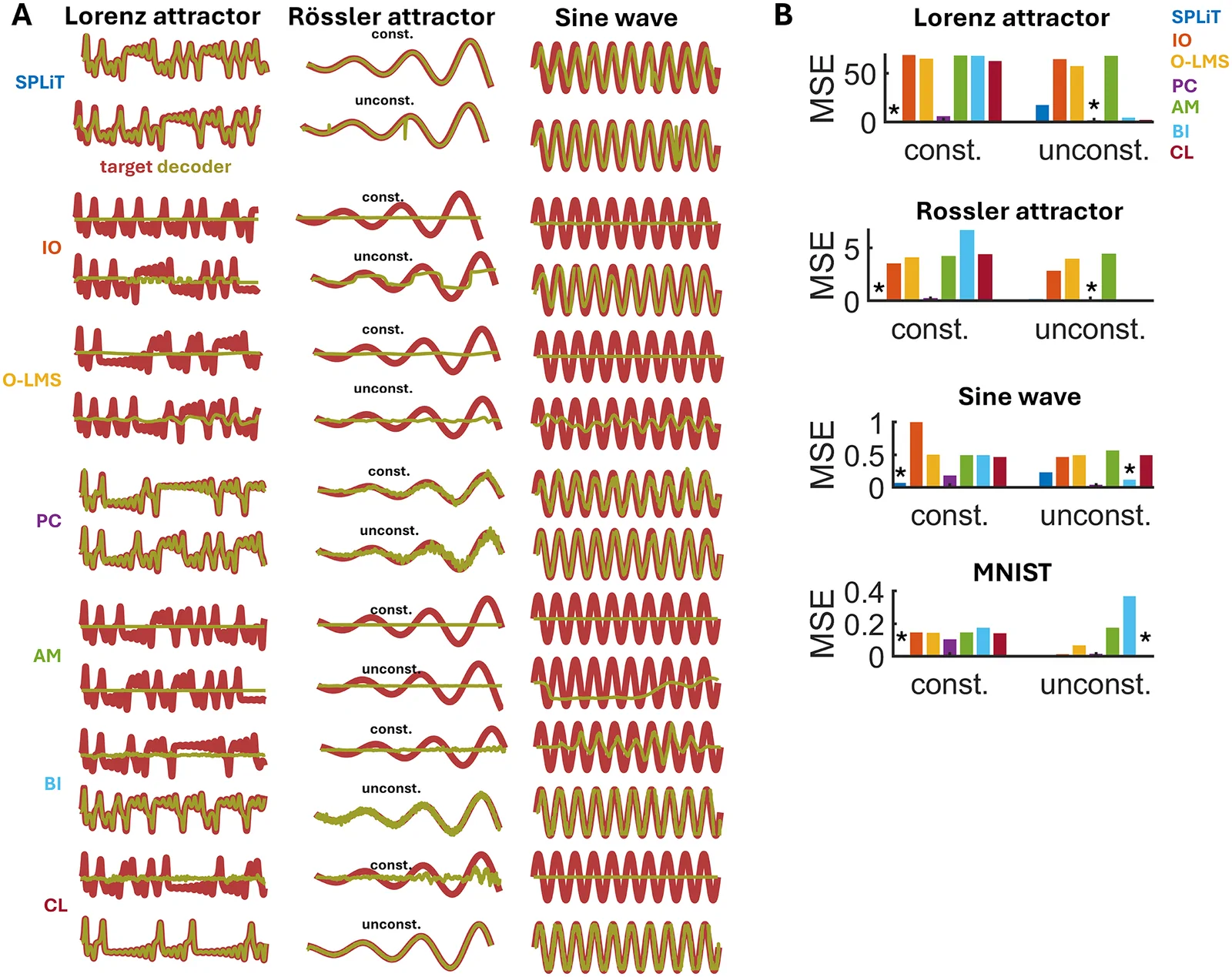
Comparison of alternative drift models trained to track temporal signals. **(A)** Synaptic learning rules were incorporated into a reservoir architecture where recurrent dynamics are either constrained to a fixed subspace (“const.”) or evolve in the full N-dimensional space (“unconst.”). **(B)** MSE across 10 independent realizations of each model (IO = ideal observer; O-LMS = online LMS; PC = predictive coding; AM = associative memory; BI = Bayesian inference; CL = continuous learning). Asterisks (“*”) indicate best performance for each target signal and network regime.

The ideal observer model failed to learn under the constrained regime (Fig 8A, 8B). In the original work describing the ideal observer model, “within-manifold learning” does not imply constraining the network state to a low-dimensional subspace. Rather, the full recurrent dynamics are preserved, and within-manifold perturbations are implemented as changes to the readout that act within the manifold coordinates. Consequently, the ideal observer model was not designed to operate under constrained neural representations.

Second, we compared SPLiT to related approaches combining Hebbian and homeostatic plasticity to stabilize drifting neural representations (O-LMS) [31,40]. Rule and O’Leary proposed that Hebbian plasticity, homeostatic regulation, and recurrent feedback could allow downstream readouts to continuously track evolving population codes without requiring explicit external error signals. Their framework demonstrated that recurrent dynamics suppress off-manifold variability by reinforcing stable population-level correlations. SPLiT shares several conceptual elements with this approach, including an online adaptation to representational drift, a low-dimensional population structure, and the use of biologically motivated plasticity rules. However, a key difference is that SPLiT integrates supervised learning with online manifold tracking. In O-LMS, the supervised error follows Eq. 20 and the decoder evolves according to


$$\begin{array}{c} \dot{D}(t)=-\eta_{\text{LMS}}e(t)r^{\text{T}}(t), \end{array}$$ (25)

with learning rate $\eta_{\text{LMS}}$=0.01. This rule depends solely on instantaneous presynaptic activity and scalar error. Because this rule is not formulated to capture temporal dependencies of the target signal, it performed poorly under constrained dynamics (Fig 8A, 8B).

Third, we considered a predictive coding (PC) rule [62–64]. Predictive coding models learn to predict future inputs rather than minimize supervised task error [62–64]. Let o(t) denote the current observation and o(t+Δt) the target at the next step. The prediction is


$$\begin{array}{c} \hat{o}(t+\Delta t)=D(t)r(t), \end{array}$$ (26)

with prediction error


$$\begin{array}{c} e_{\text{PC}}(t)=\hat{o}(t+\Delta t)-o(t+\Delta t). \end{array}$$ (27)

Readout weights are updated using a recursive least-squares rule,


$$\begin{array}{c} \dot{D}(t)=-\eta_{\text{RLS}}e_{\text{PC}}(t)g^{\text{T}}(t), \end{array}$$ (28)

where $\eta_{RLS}$=0.01 and g(t) is the gain (Eq. 22). In contrast to SPLiT, learning is driven by prediction error rather than task error, and no explicit manifold tracking is performed.

This approach performed well in both the constrained and unconstrained regimes (Fig 8A) but yielded higher error than SPLiT in the constrained scenario (Fig 8B). In general terms, predictive coding benefits from overcomplete internal representations; enforcing a low-dimensional constraint reduces representational redundancy and limits the model’s ability to capture temporal structure.

Fourth, we examined an associative memory (AM) mechanism designed to preserve an input-output association while the recurrent network undergoes drift [65]. The associative memory model updates decoder weights via covariance-based Hebbian plasticity. Let $\overset{―}{r}(t)$ and $\overset{―}{z}(t)$ denote running averages. The decoder update is


$$\begin{array}{c} \dot{D}(t)=\eta_{\text{COV}}\left(z(t)-\overset{―}{z}(t)\right){\left(r(t)-\overset{―}{r}(t)\right)}^{\text{T}}, \end{array}$$ (29)

with $\eta_{COV}=\text{0}.\text{0}\text{1}$. To prevent runaway growth, weights are normalized to maintain a fixed total synaptic strength,


$$\begin{array}{c} D(t)\leftarrow D(t)\frac{S}{{\left\|D(t)\right\|}_{\text{1}}}, \end{array}$$ (30)

where S=1 is the target total synaptic strength. This rule is unsupervised and does not incorporate an explicit task error term. The associative memory rule is not formulated to learn temporal signals and hence performed poorly for both constrained and unconstrained dynamics (Fig 8A, 8B).

Fifth, we evaluated a synaptic learning rule based on Bayesian inference (BI), designed to compensate for neuronal drift [66]. In the Bayesian inference model, the readout weights follow stochastic gradient ascent on a log-posterior distribution. Assuming Gaussian observation noise with variance $\sigma^{\text{2}}$, the gradient is expressed as


$$\begin{array}{c} \nabla_{D}\text{log}p\left(z^{*}(t)|r(t)\right)=\frac{z^{*}(t)-z(t)}{\sigma^{\text{2}}}r^{\text{T}}(t). \end{array}$$ (31)

With a Gaussian prior −λD(t), the Langevin update becomes


$$\begin{array}{c} \dot{D}(t)=\eta_{\text{BI}}\left(\frac{z^{*}(t)-z(t)}{\sigma^{\text{2}}}r^{\text{T}}(t)-\lambda D(t)\right)+\sqrt{\text{2}\eta }\xi (t), \end{array}$$ (32)

where $\eta_{\text{BI}}$=0.001. This stochastic sampling mechanism requires redundancy in the full activity space and does not exploit intrinsic manifolds. This rule performed well under unconstrained dynamics but showed poor performance in the constrained regime (Fig 8A, 8B). Intuitively, constraining the network to a fixed low-dimensional subspace removes the redundancy required for effective stochastic sampling of Bayesian inference, thereby degrading performance.

Finally, we examined a continuous-learning (CL) approach motivated by hyperplasticity [33]. In this model, synaptic plasticity consisted of a noisy delta rule applied to the readout weights, combined with a large learning rate. Weights evolve according to


$$\begin{array}{c} \dot{D}(t)=-\eta_{\text{CL}}e(t)r^{\text{T}}(t)-\varphi D(t)+\sigma_{\Delta \text{D}}\xi (t), \end{array}$$ (33)

where $\eta_{\text{CL}}$=0.5, φ=0.001, and $\sigma_{\Delta_{D}}$=0.05. No explicit manifold tracking mechanism is present. This approach performed well in the unconstrained regime but failed when the dynamics were constrained (Fig 8A, 8B). Overall, SPLiT outperformed all alternative models when recurrent dynamics were constrained to a low-dimensional manifold (Fig 8B). Further, SPLiT learning was close in performance to the top learning rules when trained with unconstrained RNNs.

### Online subspace tracking stabilizes learning under coordinate rotation

Simulations were performed with a gradual rotational drift (S8A Fig). In this scenario, the manifold basis evolves as


$$\begin{array}{c} Q(t+\Delta t)=Q(t)R\left(\Delta \theta_{t}\right), \end{array}$$ (34)

where $Q(t)\in R^{N\times k}$ is the orthonormal basis spanning the low-dimensional manifold at time t, Δt is the simulation time step, $R\left(\Delta \theta_{t}\right)\in R^{k\times k}$ is a rotation matrix, and $\Delta \theta_{t}$ is the incremental rotation angle. Under rotational drift, decoder performance depended on the strength of feedback (S8B, S8C Fig). This suggests that rotational drift relies on feedback to stabilize learning, allowing the decoder to remain aligned with the evolving population subspace. FORCE learning (S8B Fig) and alternative models did not perform accurately on this task (S9 Fig).

Next, we extended these results to the center-out reaching task with rotational perturbations. For most models, within-manifold perturbations yielded worse performance than outside-manifold perturbations (S10 Fig). This result differs from SPLiT, where performance was more sensitive to between- than within-manifold perturbations (Fig 3D), suggesting that tracking of the intrinsic low-dimensional activity subspace is critical for preserving decoder alignment under manifold drift.

To further establish whether online subspace tracking was required under rotational drift, simulations were performed where the manifold basis U(t) remained frozen once initialized. In these simulations, the frozen basis remained poorly aligned with the evolving population subspace (S11A Fig). By contrast, allowing U(t) to adapt over training improved its alignment with the evolving manifold. This improved alignment was accompanied by a lower decoding error (S11B, S11C Fig). Thus, under manifold drift, online subspace tracking contributes to maintaining decoder alignment and improving task performance.

Finally, simulations were performed to examine whether SPLiT could recover from a rotational perturbation introduced after training on the sine wave task. Small rotations produced little disruption to performance, whereas larger rotations caused progressively larger increases in error (S12 Fig). Even for large rotations, SPLiT maintained lower error than simulations where the manifold basis U(t) remained frozen, indicating that online subspace tracking improves decoding performance across a range of perturbation strengths.

## Discussion

This work introduced SPLiT, a new synaptic learning rule that tracks the low-dimensional structure of recurrent neural activity while performing supervised learning of a time-evolving target. SPLiT combines an unsupervised Oja rule to estimate the empirical activity manifold with a normalized LMS update operating in manifold coordinates. This mechanism allows the decoder to align with low-dimensional population structure during learning.

A key finding is that SPLiT enables recurrent neural networks to maintain a reliable output both when activity is constrained to a fixed low-dimensional manifold and when it is left unconstrained. In contrast, traditional LMS approaches such as FORCE learning exhibit degraded performance in the constrained regime, despite performing adequately when activity spans the full high-dimensional space. This degradation arises from a fundamental limitation of these approaches: when activity is confined to a k-dimensional manifold (where k≪N), the covariance matrix of recurrent activity becomes low-rank, with many eigenvalues approaching zero. As a result, decoder updates that depend on inverting the full covariance matrix become numerically unstable and sensitive to noise along weakly represented directions. SPLiT avoids this issue by operating within the intrinsic activity subspace.

Moreover, SPLiT exhibited reliable performance when perturbations were applied within the intrinsic manifold, but a degradation with perturbations outside of the manifold (Fig 3D). With these results, SPLiT provides a mechanism for the asymmetry between within- and outside-manifold learning observed experimentally [22]. A first series of simulations involved perturbations that imposed a misalignment relative to a fixed intrinsic manifold (Fig 3A-3C). In those simulations, the low-dimensional activity subspace remained stationary after initialization, such that subspace tracking by SPLiT served to align decoder weights with a fixed structure. To more directly model representational drift, we subsequently introduced rotational perturbations where the manifold basis evolved continuously over time (S8A Fig). Under these conditions, online adaptation of the estimated subspace became necessary to maintain alignment between decoder weights and the evolving population manifold. Simulations with a frozen manifold basis U(t) showed that manifold tracking improved both manifold alignment and decoding accuracy under drift, demonstrating that SPLiT performs online manifold tracking and not simply learning within a static low-dimensional subspace.

The novelty of SPLiT lies in the formulation of a unified framework that integrates supervised error-driven plasticity with an unsupervised rule that continuously aligns decoding weights with an evolving population subspace. To our knowledge, this is the first model to demonstrate that online manifold tracking can transform supervised learning from a poorly conditioned full-rank regression problem into a learning rule that is restricted to a time-evolving low-dimensional subspace.

From a biological standpoint, while the unsupervised subspace-tracking rule resembles experimentally supported Hebbian and Oja synaptic rules, the supervised decoder update is implemented using an explicit error signal and weight normalization for analytical tractability. While error-driven plasticity is widely hypothesized in cortical and cerebellar circuits [67–69], the exact implementation in SPLiT should be viewed as a computational model of such processes rather than a literal synaptic rule.

Simulation results derived from SPLiT align with experimental findings from brain-computer interface studies. In particular, animals can learn to control a cursor using neural activity that remains within the intrinsic neural manifold, whereas learning outside this manifold is slower [12]. Our modeling results provide a mechanism for these observations. We show that when activity patterns are constrained within a manifold, increasing the synaptic learning rate leads to a steep decline in error, whereas this decline is shallower with unconstrained activity (Fig 5C). Further, plasticity under constrained dynamics requires smaller weight changes (Fig 5D). Thus, constrained dynamics promote rapid learning by yielding a greater sensitivity to learning rates and requiring smaller adjustments to individual synapses.

Furthermore, we showed that constrained dynamics confer computational advantages including reduced sensitivity to recurrent gain and enhanced robustness to perturbations propagated through feedback. Computationally, this occurs because restricting activity to a low-dimensional subspace improves the conditioning of the regression problem faced by the decoder. Constraining activity to a low-dimensional subspace reduces the effective dimensionality of the decoder regression problem by restricting learning to the dominant covariance modes of population activity. While similar improvements in conditioning could be achieved using low-rank projections or pseudoinverse regularization, the novelty of SPLiT lies in providing a biologically motivated online mechanism for continuously estimating and tracking this subspace during learning.

Beyond improving task performance, SPLiT learning provides a biologically interpretable account of why learning constrained to intrinsic neural manifolds may proceed more rapidly than unconstrained adaptation. When manifold-constrained dynamics produce low-dimensional population activity, downstream readouts can achieve accurate decoding with relatively small synaptic modifications (Fig 5D). By contrast, unconstrained dynamics require larger and more variable weight updates to compensate for continuously evolving high-dimensional activity patterns. This distinction provides a mechanistic interpretation for experimental observations showing that within-manifold learning in motor cortex occurs on faster timescales than learning that requires activity outside of the intrinsic manifold [22]. More generally, the model suggests that low-dimensional neural population structure may reduce the synaptic burden associated with behavioral adaptation by restricting plasticity to a smaller set of behaviorally relevant degrees of freedom.

Together, these results suggest that intrinsic neural manifolds may not merely represent a statistical description of neural activity but may reflect a fundamental organization that shapes how learning rules operate within biological circuits. In this view, manifolds define the effective degrees of freedom available for rapid adaptation, while slower learning reflects the need to reshape those degrees of freedom themselves.

### Neural basis of representational drift

Recent theoretical work suggests that representational drift may not simply reflect noise, instability, or degradation of neural coding, but instead arise from ongoing synaptic plasticity within recurrent circuits [26,31,33,40]. In this perspective, neuronal populations remain plastic even after behavioral performance has stabilized, causing internal representations to reorganize over time while preserving task-relevant computations. Representational drift therefore reflects an active process of circuit remodeling rather than a passive failure of neural stability mechanisms.

Consistent with this view, Qin et al. [70] proposed that drift can emerge from noisy Hebbian learning in networks with multiple equivalent representational solutions, producing coordinated manifold rotations while preserving population-level structure. Similarly, Ratzon et al. [71] argued that drift may reflect a slow process of regularization in continually trained networks, where representations gradually reorganize toward sparser solutions despite stable task performance. Devalle et al. [72] further suggested that drift may be an unavoidable consequence of ongoing memory storage, whereby continual synaptic overwriting progressively decorrelates activity patterns. More recently, Eppler et al. [73] demonstrated that representational drift may emerge from the interaction between stochastic synaptic fluctuations and compensatory Hebbian plasticity. Together, these studies support the view that representational drift reflects an intrinsic property of adaptive neural systems undergoing continual synaptic turnover rather than a failure of neural coding. This view is consistent with experimental findings showing stable behavior despite substantial synaptic turnover [36,42–45]. An implication of these findings is that learned behavior does not require stable activity at the level of individual neurons but may instead be preserved through coordinated population dynamics combined with compensatory plasticity.

SPLiT is consistent with this interpretation of representational drift. In our framework, drift emerges because the structure of recurrent population activity evolves over time, causing the underlying manifold coordinates to reorganize. Drift can therefore occur both within the recurrent population manifold itself and indirectly at the level of decoder outputs if readout weights fail to track the evolving latent structure. Stable performance thus requires the continual adaptation of downstream readout weights to maintain alignment with ongoing changes in neural population structure. In this view, manifold stability should not be viewed as a static property of recurrent activity, but rather as a dynamic equilibrium emerging from the interaction between recurrent drift and continual plasticity in downstream readout populations.

This interpretation also provides a possible explanation for why neural manifolds often appear stable over short timescales yet gradually reorganize over longer periods [11,22]. Fast compensatory plasticity may preserve enough task-relevant structure to maintain behavior, while slower synaptic turnover and learning processes progressively reshape the underlying population structure. From this perspective, representational drift and behavioral stability are not contradictory phenomena, but reflect consequences of continual learning in neuronal circuits.

### Related approaches

Our findings are related to several recent modeling approaches but differ from them in important ways. Crucially, systematic comparisons between SPLiT and related plasticity rules using the same tasks and architecture revealed that none of the alternatives could learn with both constrained and unconstrained regimes. Comparisons included the following rules:

(1) *Ideal observer.* In related work by Feulner and Clopath (2021), a model was shown to learn both within- and outside-manifold perturbations [55]. Furthermore, within-manifold learning in the ideal observer model was more robust to misleading feedback. SPLiT extends this work by positing an explicit, biologically inspired plasticity mechanism to adapt readout weights in tandem with evolving population subspaces.
(2) *Online LMS.* Another relevant line of work proposed a synaptic mechanism for tracking representational drift by combining Hebbian and homeostatic plasticity [31,40]. In these studies, drift emerges from ongoing synaptic turnover and gradual reorganization of neural representations. Subsequent experimental and theoretical work has shown that such drift manifests as incremental changes in low-dimensional population manifolds while preserving task-relevant structure [22, 36]. SPLiT builds on this view of representational drift. However, unlike previous approaches, SPLiT combines online subspace tracking with a supervised decoder.
(3) *Predictive coding.* Learning rules based on predictive coding assume that circuits continuously minimize prediction errors arising from mismatches between internal latent states and external inputs [62–64]. Depending on the architecture, predictive coding frameworks can incorporate supervised learning signals. The key difference relative to SPLiT lies in how representational drift is handled. Predictive coding models generally optimize latent-state inferences and prediction consistency, whereas SPLiT performs online tracking of evolving low-dimensional population subspaces through manifold estimation.
(4) *Associative memory, Bayesian inference, and continuous learning.* Other models address representational drift through associative memory mechanisms [65], a stability-flexibility trade-off [26,66], continuous learning [33], or an exploration of degenerate representational manifolds during Hebbian learning [70]**.** In contrast to these approaches, we take drift as a starting point based on experimental findings and ask how a plasticity rule can track it to perform a task. From this perspective, drift is not something to be minimized or regulated, but a phenomenon to be accommodated through a compensatory synaptic plasticity mechanism such as SPLiT learning.
(5) *FORCE learning*. While FORCE learning is a well-established supervised learning algorithm for training recurrent neural networks to generate complex trajectories [53], it performed poorly when recurrent dynamics are constrained to a fixed low-dimensional manifold (Fig 2C). This failure happens because FORCE relies on recursive least-squares updates in the full population space and therefore assumes sufficiently rich, full-rank activity covariance. When activity is confined to a low-dimensional manifold, many directions in neuron space become unavailable, rendering covariance inversion ill-conditioned. Thus, the constrained regime considered here violates a core assumption underlying FORCE learning. We nevertheless included this comparison because it illustrates a broader point central to the present work: learning rules that operate directly in the full activity space can become unstable when neural population activity is intrinsically low-dimensional.

Importantly, impairments under constrained dynamics were also observed for several alternative plasticity rules that do not explicitly rely on full covariance inversion (Fig 8), including O-LMS, predictive coding, associative memory, Bayesian inference, and continuous-learning approaches. The comparison to FORCE should therefore not be interpreted primarily as a criticism of recursive least-squares methods themselves, but rather as an illustration that learning rules operating in the full neural activity space can degrade when population activity is low-dimensional.

Variants of FORCE learning that account for low-dimensional manifolds could likely mitigate representational drift by incorporating regularization or low-dimensional projections. However, our current goal was to provide a biologically interpretable online plasticity mechanism that performs manifold tracking and supervised learning within a cohesive framework. Finally, we note that several studies have applied Oja’s rule or related Hebbian normalization schemes to recurrent neural networks [74–83]. However, these studies did not consider Oja’s rule as a mechanism for tracking fluctuations in low-dimensional neural manifolds. The novelty of SPLiT lies in using Oja’s rule as a way to continuously estimate an evolving population subspace, which is then leveraged for supervised learning.

### Experimental predictions

Two experimental predictions of SPLiT learning can be articulated along with brain-computer interface (BCI) paradigms designed to test them.

First, SPLiT predicts that recovery from outside-manifold perturbations depends not only on changing decoder weights, but also on continually tracking the evolving structure of population activity. In our simulations, freezing the manifold estimate U(t) reduced performance even when the decoder was still allowed to adapt (S12 Fig). Experimentally, this idea could be tested by allowing a BCI decoder to remain plastic while holding fixed the neural subspace used by the decoder. SPLiT predicts that recovery should be impaired when decoder plasticity is preserved but online updating of the neural manifold is prevented.

Second, SPLiT assumes a separation of timescales between the relatively slow evolution of the low-dimensional manifold and the faster updates of the manifold-tracking rule. A prediction derived from this assumption is that improving the speed or accuracy of manifold tracking should accelerate recovery from outside-manifold perturbations. Experimentally, this prediction could be tested in a closed-loop BCI system by comparing decoders that update estimates of the neural activity manifold at different timescales. SPLiT predicts that, up to a point, faster manifold tracking should improve decoder-manifold alignment, accelerate recovery from outside-manifold perturbations, and reduce sensitivity to noise in the target signal.

This prediction is related to prior adaptive BCI approaches that incorporate online feature learning prior to readout adaptation, such as the GP-DKF framework of Brandman et al. [84]. However, SPLiT makes a more specific prediction: adaptation speed should depend not merely on adaptive feature learning per se, but on the ability of the decoder to maintain alignment with the evolving low-dimensional covariance structure of neural population activity. In SPLiT, recovery from outside-manifold perturbations depends on online tracking of the intrinsic activity subspace itself, rather than solely on nonlinear decoding.

### Future directions and conclusions

One open question concerns how plasticity could reshape information flow between communicating brain areas to selectively transfer within-manifold or outside-manifold information [9]. Recent theoretical work suggests that inter-areal communication may depend critically on manifold alignment [85]. An intriguing possibility is that SPLiT could allow a receiving area to continuously estimate and track the low-dimensional covariance structure of a sender population while restricting supervised adaptation to the instantaneous intrinsic subspace. While related models have proposed adaptive readouts capable of compensating for representational drift [31,70], SPLiT differs in that manifold tracking is treated as a key component of learning that is distinct from the decoder. In this framework, successful adaptation depends not only on decoder adaptation but on the alignment between decoder coordinates and the evolving activity manifold through online estimation. This predicts that inter-area communication under drift should depend on the trackability of low-dimensional population structure rather than solely on readout plasticity.

Although the present study focused on decoder adaptation within a reservoir framework, the manifold-tracking component of SPLiT could, in principle, be combined with biologically-motivated learning rules for recurrent neural networks such as e-prop or RFLO [46,48], allowing plasticity within the network to be constrained by an evolving low-dimensional population structure.

Further modeling efforts based on SPLiT will focus on adding biological constraints to the learning rule. The model employs rate-based neurons, explicit orthonormalization, and instantaneous access to global activity statistics to compute principal components. Real cortical circuits would approximate these operations through local synaptic plasticity rules. Moreover, the supervised decoder update assumes continuous error availability, whereas biological error signals are likely sparse or delayed relative to the fast timecourse of recurrent activity within local circuits. Finally, SPLiT assumes a linear manifold structure, whereas biological circuits may not be constrained to such simple structure.

Despite these limitations, incorporating more refined links to neural circuit biology will likely not overturn the computational principles of SPLiT, namely that a combination of unsupervised manifold tracking with supervised error-driven plasticity provides an efficient mechanism for learning under drifting population dynamics. Any neural circuit where population activity lies on evolving low-dimensional manifolds could, in theory, benefit from a plasticity rule that tracks this structure. Thus, SPLiT may provide a general computational principle linking synaptic plasticity to neural structure and behavioral adaptation.

In conclusion, we introduced SPLiT as a biologically motivated synaptic learning rule that reconciles supervised learning with drifting low-dimensional neural manifolds. By tracking population structure and restricting learning to intrinsic subspaces, SPLiT enables stable learning in regimes where standard LMS approaches fail. These results provide a computational account for the robustness of learning within intrinsic neural manifolds and suggest that plasticity rules that track ongoing population dynamics may be a key ingredient of computation in neuronal circuits.

## Methods

### Estimating changes in activity subspace

To quantify changes in the empirical activity subspace over time, we computed the principal angles between the initial empirical manifold and the activity manifold at subsequent time windows. This metric captures drift in the empirical covariance structure, and not displacement relative to the subspace Q. Let $U_{\text{0}}$ denote an orthonormal basis obtained by applying singular value decomposition to the mean-centered activity of Eq. 1 over an initial time window of fixed duration (100 ms). At time t, the basis $U_{t}$ was obtained in a similar fashion using sliding windows. We formed the cross-correlation matrix


$$\begin{array}{c} C_{t}=U_{\text{0}}^{\text{T}}U_{t}. \end{array}$$ (35)

Let $\left\{\sigma_{i}\left(C_{t}\right)\right\}$ be its singular values. The principal angles are


$$\begin{array}{c} \theta_{i}(t)=\text{arccos}\left(\sigma_{i}\left(C_{t}\right)\right), \end{array}$$ (36)

and changes in activity subspace can be summarized by the mean principal angle


$$\begin{array}{c} \overset{―}{\theta }(t)=\frac{\text{1}}{k}\sum_{i=\text{1}}^{k}\theta_{i}(t). \end{array}$$ (37)

The mean angle $\overset{―}{\theta }(t)$ provides a measure of how much the network’s activity subspace departs from the initial reference manifold. While other drift metrics may be informative, such as maximum angle, subspace overlap, or total subspace distance, Eq. 37 provides a useful summary of the empirical drift in the manifold over time. In unconstrained networks, increases in principal angle should be interpreted as deviations from the initial dominant activity subspace rather than evidence for a rotating manifold.

### Lorenz and Rössler attractors

The Lorenz attractor was defined by


$$\begin{array}{c} \dot{x}=\sigma (y-x), \end{array}$$

$$\begin{array}{c} \dot{y}=x(\rho -z)-y, \end{array}$$

$$\begin{array}{c} \dot{z}=xy-\beta z, \end{array}$$ (38)

with parameters σ =10, ρ = 28, and β = 8/3. For the Rössler attractor,


$$\begin{array}{c} \dot{x}=-y-z, \end{array}$$

$$\begin{array}{c} \dot{y}=x+ay, \end{array}$$

$$\begin{array}{c} \dot{z}=b+z(x-c), \end{array}$$ (39)

with parameters a = 0.2, b = 0.2, and c = 5.7.

## Supporting information

S1 Appendix Supporting information and supplementary figures. (DOCX)

S1 Code Code.zip. (ZIP)

S1 FigImpact of manifold adaptation rate ($\eta_{U}$) (A) and number of recurrent neurons (N) (B) on performance in the MNIST task.(TIF)

S2 FigSimulations where activity is initialized outside of the manifold.(A) Schema showing how SPLiT can realign the basis when the initial state of the decoder is an off-manifold basis. (B) Mean principal angle between the manifold basis Q(t) and the estimated decoder subspace U(t). Different curves show values of manifold rotation angle (θ). (C) MSE across rotation angles.(TIF)

S3 FigImpact of adding noise to the feedback signal.Solid line: Δr refers to the difference between the recurrent activity when noise is added to the MNIST target signal (zero mean, variance of 1) versus no noise. Values are means across ten independent realizations with frozen initial conditions but different target noise. Dashed line: Δr is the difference between the recurrent activity across independent simulations with no target noise. Values are means over ten independent realizations with different initial conditions.(TIF)

S4 FigImpact of recurrent gain and feedback strength.(A) In the constrained learning condition, increasing the feedback strength (α) reduces MSE during the MNIST tracing task. Altering the gain (g) of recurrent weights had no impact on MSE. (B) Mean ratio of the relative drive of feedback and recurrent activity (Eq. 18).(TIF)

S5 FigSimulation that matched the variance of recurrent activity in the constrained and unconstrained conditions.Dashed lines: best-fitting power laws. For constrained dynamics: R^2^ = 0.797, β=1.350). For unconstrained dynamics: R^2^ = 0.755 (β=0.001).(TIF)

S6 FigThe rank of the learning feedback ($I_{𝐟𝐛}$) has minimal impact on error when altering the variance of the random noise injected into the target signal.Feedback was set to either rank-1 (solid lines) or rank-k (dashed lines).(TIF)

S7 Fig Comparison of drift models trained to track oscillations of various frequencies (1 Hz, 5 Hz, and 10 Hz). MSE values are means over 10 independent realizations of each model. Right column shows a single run of SPLiT learning for each oscillation frequency.
(TIF)

S8 FigPerformance of SPLiT with rotational drift.(A) Schematic representing a rotation of the manifold coordinates. (B) Impact of learning feedback ($I_{\text{fb}}$) on training error (MSE) in a task where the decoder tracked a 1 Hz sine wave. (C) Examples of decoder output and target traces across varying strengths of learning feedback ($I_{\text{fb}}$).(TIF)

S9 Fig Task involving tracking a sine wave when the manifold undergoes rotational drift. Different models (A-F) attain poor performance compared to SPLiT (see Suppl. Fig S8C).
(TIF)

S10 Fig Manifold perturbations during a two-dimensional center-out reaching task. The performance of six models (A-F) is compared across different angles of manifold rotation applied during the perturbation. Simulations where the dynamics are constrained to a low-dimensional manifold (within-manifold) are compared to a condition where dynamics are unconstrained (outside-manifold).
(TIF)

S11 FigComparison of frozen versus adaptive decoder subspace U(t) in the sine wave task.(A) The mean principal angle remains high when U(t) is frozen but decreases when continuously adapted by SPLiT. (B) Error is higher when U(t) is frozen. (C) Example of traces obtained with frozen vs. adaptive U(t).(TIF)

S12 FigIntroducing a rotational perturbation in the sine wave task.Increasing the angle of manifold rotation (from 25 to 80 degrees) lowers task performance. Overall, error remains lower with adaptive U(t) compared to a condition where manifold adaptation remains frozen during training.(TIF)
