# Supplementary material for: Manifold-constrained plasticity enables stable learning in recurrent neural circuits
Source: PLoS Comput Biol. 2026 Aug 25;22(8):e1014719. doi: 10.1371/journal.pcbi.1014719 (PMC13529248; doi:10.1371/journal.pcbi.1014719)
Supplement: Supplementary file 1 [file pcbi.1014719.s001.docx]

**Supporting Information**

**Double Shrinkage Transfer Causal Learning: An Application to Alzheimer's Disease**

**S1 Appendix: Supplementary simulation and real-data analyses.**

[Supplementary simulation methods	2](#_Toc1767166393)

[Main simulation results	2](#_Toc1066729141)

[Table A. Results across methods as the source domain sample size varies (Scenario 1).	2](#_Toc1323187631)

[Table B. Results across methods as h-transferability varies (Scenario 2).	4](#_Toc1621349532)

[Table C. Results across methods as the covariate dimension varies (Scenario 3).	6](#_Toc601587541)

[Additional simulation sensitivity analyses	8](#_Toc528791511)

[Table D. Regularization sensitivity analysis for DSTCL.	8](#_Toc1111594091)

[Table E. Sensitivity analysis under increasingly extreme true propensity scores.	8](#_Toc1601642184)

[Table F. Trimming sensitivity analysis for DSTCL.	9](#_Toc92671343)

[Table G. Covariate-shift sensitivity analysis for DSTCL.	9](#_Toc601617726)

[Table H. Computational time and convergence diagnostics for DSTCL as the covariate dimension increases.	10](#_Toc1036110806)

[Real-data supplementary analyses	11](#_Toc2116906566)

[Table I. Variable-level source-target distribution diagnostics.	11](#_Toc1487074913)

[Table J. Propensity-score and effective-sample-size diagnostics in the ADNI analyses.	13](#_Toc853379005)

[Table K. DSTCL estimates from the ADNI imputation analyses.	13](#_Toc1845142369)

# Supplementary simulation methods

Unless otherwise specified, the target domain sample size was 50, each simulation setting was repeated 200 times, and percentile confidence intervals were constructed using 500 bootstrap resamples within each Monte Carlo replication. Performance was summarized using bias, empirical standard deviation (SD), mean squared error (MSE), and coverage.

Three model-specification regimes were considered: both propensity score (PS) and outcome regression (OR) models correctly specified, only the PS model correctly specified, and only the OR model correctly specified.

The main simulations varied the source domain sample size, h-transferability level, and covariate dimension. Additional analyses evaluated penalty-selection rules, increasingly extreme true propensity scores, propensity score trimming, target domain covariate shift, computation time, convergence.

## Main simulation results

**Table A. Results across methods as the source domain sample size varies (Scenario 1).**

**(a) Both PS and OR correct**

| **Source sample size** | **Estimator** | **Bias (SD)** | **MSE** | **Coverage** |
| --- | --- | --- | --- | --- |
| m=500 | Crude difference | 0.500 (0.890) | 1.040 | 0.880 |
|  | Known parameters | -0.040 (0.270) | 0.070 | 0.900 |
|  | GLM | - | - | - |
|  | Lasso | 0.540 (0.830) | 0.980 | 0.760 |
|  | DML1 | 0.440 (0.860) | 0.930 | 0.880 |
|  | DML2 | 0.460 (0.900) | 1.010 | 0.880 |
|  | TMLE | 0.500 (0.910) | 1.080 | 1.000 |
|  | l_1_-TCL | - | - | - |
|  | DSTCL | 0.090 (0.270) | 0.080 | 0.980 |
| m=1000 | Crude difference | 0.420 (0.872) | 0.933 | 0.915 |
|  | Known parameters | 0.001 (0.331) | 0.109 | 0.940 |
|  | GLM | - | - | - |
|  | Lasso | 0.448 (0.822) | 0.872 | 0.775 |
|  | DML1 | 0.280 (0.790) | 0.690 | 0.920 |
|  | DML2 | 0.250 (0.830) | 0.760 | 0.920 |
|  | TMLE | 0.340 (0.840) | 0.820 | 0.990 |
|  | l_1_-TCL | - | - | - |
|  | DSTCL | 0.064 (0.255) | 0.069 | 0.940 |
| m=2000 | Crude difference | 0.494 (0.814) | 0.903 | 0.910 |
|  | Known parameters | 0.024 (0.236) | 0.056 | 0.915 |
|  | GLM | - | - | - |
|  | Lasso | 0.518 (0.785) | 0.881 | 0.745 |
|  | DML1 | 0.440 (0.860) | 0.940 | 0.900 |
|  | DML2 | 0.380 (0.820) | 0.810 | 0.900 |
|  | TMLE | 0.330 (0.920) | 0.960 | 0.980 |
|  | l_1_-TCL | 0.240 (2.490) | 6.220 | 0.990 |
|  | DSTCL | 0.084 (0.271) | 0.080 | 0.900 |

**(b) PS correct only**

| **Source sample size** | **Estimator** | **Bias (SD)** | **MSE** | **Coverage** |
| --- | --- | --- | --- | --- |
| m=500 | Crude difference | 1.250 (0.510) | 1.830 | 0.270 |
|  | Known parameters | -0.120 (1.610) | 2.590 | 0.880 |
|  | GLM | - | - | - |
|  | Lasso | 1.230 (0.500) | 1.770 | 0.090 |
|  | DML1 | 1.110 (0.490) | 1.480 | 0.300 |
|  | DML2 | 1.120 (0.510) | 1.520 | 0.270 |
|  | TMLE | 0.860 (0.540) | 1.030 | 0.750 |
|  | l_1_-TCL | - | - | - |
|  | DSTCL | 0.170 (0.260) | 0.090 | 0.940 |
| m=1000 | Crude difference | 1.220 (0.450) | 1.690 | 0.260 |
|  | Known parameters | 0.190 (1.600) | 2.580 | 0.900 |
|  | GLM | - | - | - |
|  | Lasso | 1.210 (0.440) | 1.650 | 0.060 |
|  | DML1 | 1.200 (0.540) | 1.720 | 0.280 |
|  | DML2 | 1.190 (0.520) | 1.680 | 0.270 |
|  | TMLE | 0.890 (0.510) | 1.050 | 0.730 |
|  | l_1_-TCL | - | - | - |
|  | DSTCL | 0.120 (0.240) | 0.070 | 0.920 |
| m=2000 | Crude difference | 1.250 (0.480) | 1.800 | 0.240 |
|  | Known parameters | -0.050 (1.300) | 1.670 | 0.920 |
|  | GLM | - | - | - |
|  | Lasso | 1.240 (0.450) | 1.740 | 0.060 |
|  | DML1 | 1.190 (0.480) | 1.650 | 0.230 |
|  | DML2 | 1.200 (0.520) | 1.700 | 0.230 |
|  | TMLE | 0.940 (0.520) | 1.150 | 0.700 |
|  | l_1_-TCL | 0.230 (2.490) | 6.210 | 0.990 |
|  | DSTCL | 0.120 (0.260) | 0.080 | 0.880 |

**(c) OR correct only**

| **Source sample size** | **Estimator** | **Bias (SD)** | **MSE** | **Coverage** |
| --- | --- | --- | --- | --- |
| m=500 | Crude difference | 0.470 (0.870) | 0.970 | 0.900 |
|  | Known parameters | -0.010 (0.300) | 0.090 | 0.950 |
|  | GLM | - | - | - |
|  | Lasso | 0.510 (0.820) | 0.930 | 0.740 |
|  | DML1 | 0.400 (0.830) | 0.840 | 0.900 |
|  | DML2 | 0.350 (0.840) | 0.810 | 0.900 |
|  | TMLE | 0.440 (0.860) | 0.940 | 0.980 |
|  | l_1_-TCL | - | - | - |
|  | DSTCL | 0.110 (0.250) | 0.070 | 0.960 |
| m=1000 | Crude difference | 0.360 (0.860) | 0.870 | 0.940 |
|  | Known parameters | 0.020 (0.320) | 0.100 | 0.900 |
|  | GLM | - | - | - |
|  | Lasso | 0.410 (0.790) | 0.790 | 0.800 |
|  | DML1 | 0.530 (0.840) | 0.970 | 0.870 |
|  | DML2 | 0.520 (0.830) | 0.960 | 0.880 |
|  | TMLE | 0.520 (0.920) | 1.120 | 0.960 |
|  | l_1_-TCL | - | - | - |
|  | DSTCL | 0.070 (0.250) | 0.070 | 0.960 |
| m=2000 | Crude difference | 0.430 (0.790) | 0.820 | 0.920 |
|  | Known parameters | -0.020 (0.200) | 0.040 | 0.940 |
|  | GLM | - | - | - |
|  | Lasso | 0.470 (0.760) | 0.800 | 0.790 |
|  | DML1 | 0.388 (0.851) | 0.870 | 0.925 |
|  | DML2 | 0.338 (0.805) | 0.759 | 0.905 |
|  | TMLE | 0.396 (0.889) | 0.943 | 0.985 |
|  | l_1_-TCL | 0.150 (2.260) | 5.090 | 1.000 |
|  | DSTCL | 0.040 (0.250) | 0.060 | 0.960 |

“–” indicates that the corresponding result was unavailable. GLM was not estimable in these settings; for l_1_-TCL, it indicates that the simulation procedure was not successfully completed in all replications.

**Table B. Results across methods as h-transferability varies (Scenario 2).**

**(a) Both PS and OR correct**

| **h-transferability** | **Estimator** | **Bias (SD)** | **MSE** | **Coverage** |
| --- | --- | --- | --- | --- |
| h=6 | Crude difference | 0.420 (0.872) | 0.933 | 0.915 |
|  | Known parameters | 0.001 (0.331) | 0.109 | 0.940 |
|  | GLM | - | - | - |
|  | Lasso | 0.448 (0.822) | 0.872 | 0.775 |
|  | DML1 | 0.280 (0.790) | 0.690 | 0.920 |
|  | DML2 | 0.250 (0.830) | 0.760 | 0.920 |
|  | TMLE | 0.340 (0.840) | 0.820 | 0.990 |
|  | l_1_-TCL | - | - | - |
|  | DSTCL | 0.064 (0.255) | 0.069 | 0.940 |
| h=11 | Crude difference | 0.420 (0.830) | 0.870 | 0.880 |
|  | Known parameters | 0.000 (0.250) | 0.060 | 0.920 |
|  | GLM | - | - | - |
|  | Lasso | 0.440 (0.800) | 0.830 | 0.780 |
|  | DML1 | 0.370 (0.810) | 0.780 | 0.920 |
|  | DML2 | 0.350 (0.820) | 0.790 | 0.890 |
|  | TMLE | 0.450 (0.820) | 0.870 | 0.980 |
|  | l_1_-TCL | - | - | - |
|  | DSTCL | 0.130 (0.320) | 0.120 | 0.960 |
| h=16 | Crude difference | 0.509 (0.807) | 0.907 | 0.855 |
|  | Known parameters | 0.022 (0.340) | 0.116 | 0.915 |
|  | GLM | - | - | - |
|  | Lasso | 0.543 (0.753) | 0.859 | 0.715 |
|  | DML1 | 0.410 (0.750) | 0.730 | 0.880 |
|  | DML2 | 0.400 (0.770) | 0.740 | 0.880 |
|  | TMLE | 0.480 (0.800) | 0.860 | 0.990 |
|  | l_1_-TCL | - | - | - |
|  | DSTCL | 0.192 (0.415) | 0.208 | 0.920 |

**(b) PS correct only**

| **h-transferability** | **Estimator** | **Bias (SD)** | **MSE** | **Coverage** |
| --- | --- | --- | --- | --- |
| h=6 | Crude difference | 1.220 (0.450) | 1.690 | 0.260 |
|  | Known parameters | 0.190 (1.600) | 2.580 | 0.900 |
|  | GLM | - | - | - |
|  | Lasso | 1.210 (0.440) | 1.650 | 0.060 |
|  | DML1 | 1.200 (0.540) | 1.720 | 0.280 |
|  | DML2 | 1.190 (0.520) | 1.680 | 0.270 |
|  | TMLE | 0.890 (0.510) | 1.050 | 0.730 |
|  | l_1_-TCL | - | - | - |
|  | DSTCL | 0.120 (0.240) | 0.070 | 0.920 |
| h=11 | Crude difference | 0.850 (0.390) | 0.880 | 0.440 |
|  | Known parameters | -0.020 (1.120) | 1.250 | 0.900 |
|  | GLM | - | - | - |
|  | Lasso | 0.840 (0.370) | 0.850 | 0.140 |
|  | DML1 | 0.820 (0.410) | 0.840 | 0.410 |
|  | DML2 | 0.810 (0.440) | 0.840 | 0.420 |
|  | TMLE | 0.610 (0.450) | 0.570 | 0.860 |
|  | l_1_-TCL | - | - | - |
|  | DSTCL | 0.150 (0.300) | 0.110 | 0.940 |
| h=16 | Crude difference | 0.510 (0.360) | 0.390 | 0.690 |
|  | Known parameters | 0.180 (1.370) | 1.900 | 0.890 |
|  | GLM | - | - | - |
|  | Lasso | 0.510 (0.340) | 0.380 | 0.340 |
|  | DML1 | 0.420 (0.360) | 0.300 | 0.700 |
|  | DML2 | 0.420 (0.350) | 0.300 | 0.690 |
|  | TMLE | 0.210 (0.380) | 0.190 | 0.980 |
|  | l_1_-TCL | - | - | - |
|  | DSTCL | 0.190 (0.390) | 0.190 | 0.920 |

**(c) OR correct only**

| **h-transferability** | **Estimator** | **Bias (SD)** | **MSE** | **Coverage** |
| --- | --- | --- | --- | --- |
| h=6 | Crude difference | 0.360 (0.860) | 0.870 | 0.940 |
|  | Known parameters | 0.020 (0.320) | 0.100 | 0.900 |
|  | GLM | - | - | - |
|  | Lasso | 0.410 (0.790) | 0.790 | 0.800 |
|  | DML1 | 0.530 (0.840) | 0.970 | 0.870 |
|  | DML2 | 0.520 (0.830) | 0.960 | 0.880 |
|  | TMLE | 0.520 (0.920) | 1.120 | 0.960 |
|  | l_1_-TCL | - | - | - |
|  | DSTCL | 0.070 (0.250) | 0.070 | 0.960 |
| h=11 | Crude difference | 0.460 (0.800) | 0.850 | 0.900 |
|  | Known parameters | -0.070 (0.330) | 0.110 | 0.940 |
|  | GLM | - | - | - |
|  | Lasso | 0.480 (0.770) | 0.820 | 0.780 |
|  | DML1 | 0.350 (0.760) | 0.700 | 0.920 |
|  | DML2 | 0.330 (0.770) | 0.700 | 0.900 |
|  | TMLE | 0.410 (0.850) | 0.880 | 0.990 |
|  | l_1_-TCL | - | - | - |
|  | DSTCL | 0.090 (0.310) | 0.100 | 0.980 |
| h=16 | Crude difference | 0.470 (0.780) | 0.830 | 0.900 |
|  | Known parameters | -0.010 (0.220) | 0.050 | 0.940 |
|  | GLM | - | - | - |
|  | Lasso | 0.520 (0.760) | 0.850 | 0.720 |
|  | DML1 | 0.470 (0.730) | 0.740 | 0.890 |
|  | DML2 | 0.450 (0.740) | 0.740 | 0.870 |
|  | TMLE | 0.430 (0.860) | 0.920 | 0.980 |
|  | l_1_-TCL | - | - | - |
|  | DSTCL | 0.180 (0.380) | 0.180 | 0.940 |

“–” indicates that the corresponding result was unavailable. GLM was not estimable in these settings; for l_1_-TCL, it indicates that the simulation procedure was not successfully completed in all replications.

**Table C. Results across methods as the covariate dimension varies (Scenario 3).**

**(a) Both PS and OR correct**

| **Covariates** | **Estimator** | **Bias (SD)** | **MSE** | **Coverage** |
| --- | --- | --- | --- | --- |
| p=50 | Crude difference | 0.440 (0.830) | 0.880 | 0.920 |
|  | Known parameters | 0.000 (0.230) | 0.050 | 0.950 |
|  | GLM | - | - | - |
|  | Lasso | 0.480 (0.720) | 0.750 | 0.790 |
|  | DML1 | 0.460 (0.790) | 0.840 | 0.880 |
|  | DML2 | 0.510 (0.790) | 0.880 | 0.880 |
|  | TMLE | 0.230 (0.790) | 0.670 | 0.990 |
|  | l_1_-TCL | 0.260 (1.370) | 1.940 | 0.980 |
|  | DSTCL | 0.050 (0.260) | 0.070 | 0.950 |
| p=100 | Crude difference | 0.300 (0.850) | 0.800 | 0.920 |
|  | Known parameters | -0.040 (0.560) | 0.310 | 0.920 |
|  | GLM | - | - | - |
|  | Lasso | 0.340 (0.820) | 0.780 | 0.800 |
|  | DML1 | 0.370 (0.810) | 0.800 | 0.910 |
|  | DML2 | 0.390 (0.790) | 0.780 | 0.900 |
|  | TMLE | 0.400 (0.940) | 1.050 | 0.990 |
|  | l1-TCL | 0.210 (2.400) | 5.800 | 0.990 |
|  | DSTCL | 0.060 (0.270) | 0.080 | 0.970 |
| p=200 | Crude difference | 0.420 (0.872) | 0.933 | 0.915 |
|  | Known parameters | 0.001 (0.331) | 0.109 | 0.940 |
|  | GLM | - | - | - |
|  | Lasso | 0.448 (0.822) | 0.872 | 0.775 |
|  | DML1 | 0.280 (0.790) | 0.690 | 0.920 |
|  | DML2 | 0.250 (0.830) | 0.760 | 0.920 |
|  | TMLE | 0.340 (0.840) | 0.820 | 0.990 |
|  | l_1_-TCL | - | - | - |
|  | DSTCL | 0.064 (0.255) | 0.069 | 0.940 |

**(b) PS correct only**

| **Covariates** | **Estimator** | **Bias (SD)** | **MSE** | **Coverage** |
| --- | --- | --- | --- | --- |
| p=50 | Crude difference | 0.030 (0.160) | 0.030 | 0.940 |
|  | Known parameters | -0.060 (1.260) | 1.590 | 0.940 |
|  | GLM | - | - | - |
|  | Lasso | 0.100 (0.180) | 0.040 | 0.720 |
|  | DML1 | -0.010 (0.160) | 0.030 | 0.960 |
|  | DML2 | -0.010 (0.160) | 0.020 | 0.950 |
|  | TMLE | -0.070 (0.240) | 0.060 | 0.990 |
|  | l_1_-TCL | 0.260 (1.370) | 1.950 | 0.980 |
|  | DSTCL | 0.040 (0.230) | 0.060 | 0.960 |
| p=100 | Crude difference | 0.370 (0.340) | 0.250 | 0.760 |
|  | Known parameters | 0.080 (1.430) | 2.030 | 0.930 |
|  | GLM | - | - | - |
|  | Lasso | 0.380 (0.330) | 0.250 | 0.480 |
|  | DML1 | 0.360 (0.300) | 0.220 | 0.720 |
|  | DML2 | 0.380 (0.300) | 0.240 | 0.690 |
|  | TMLE | 0.140 (0.300) | 0.110 | 0.980 |
|  | l_1_-TCL | 0.210 (2.420) | 5.880 | 0.990 |
|  | DSTCL | 0.060 (0.250) | 0.060 | 0.940 |
| p=200 | Crude difference | 1.220 (0.450) | 1.690 | 0.260 |
|  | Known parameters | 0.190 (1.600) | 2.580 | 0.900 |
|  | GLM | - | - | - |
|  | Lasso | 1.210 (0.440) | 1.650 | 0.060 |
|  | DML1 | 1.200 (0.540) | 1.720 | 0.280 |
|  | DML2 | 1.190 (0.520) | 1.680 | 0.270 |
|  | TMLE | 0.890 (0.510) | 1.050 | 0.730 |
|  | l_1_-TCL | - | - | - |
|  | DSTCL | 0.120 (0.240) | 0.070 | 0.920 |

**(c) OR correct only**

| **Covariates** | **Estimator** | **Bias (SD)** | **MSE** | **Coverage** |
| --- | --- | --- | --- | --- |
| p=50 | Crude difference | 2.050 (0.820) | 4.880 | 0.260 |
|  | Known parameters | 0.170 (2.000) | 4.010 | 0.920 |
|  | GLM | - | - | - |
|  | Lasso | 1.850 (0.760) | 3.990 | 0.140 |
|  | DML1 | 1.700 (0.730) | 3.430 | 0.290 |
|  | DML2 | 1.760 (0.720) | 3.620 | 0.240 |
|  | TMLE | 0.850 (0.660) | 1.150 | 0.820 |
|  | l_1_-TCL | 0.090 (0.440) | 0.200 | 0.950 |
|  | DSTCL | 0.080 (0.260) | 0.080 | 0.910 |
| p=100 | Crude difference | 0.490 (0.870) | 1.000 | 0.900 |
|  | Known parameters | -0.030 (0.400) | 0.160 | 0.950 |
|  | GLM | - | - | - |
|  | Lasso | 0.550 (0.800) | 0.950 | 0.780 |
|  | DML1 | 0.330 (0.880) | 0.880 | 0.870 |
|  | DML2 | 0.370 (0.890) | 0.920 | 0.850 |
|  | TMLE | 0.320 (0.930) | 0.970 | 0.980 |
|  | l_1_-TCL | 0.180 (3.230) | 10.400 | 1.000 |
|  | DSTCL | 0.070 (0.250) | 0.070 | 0.970 |
| p=200 | Crude difference | 0.360 (0.860) | 0.870 | 0.940 |
|  | Known parameters | 0.020 (0.320) | 0.100 | 0.900 |
|  | GLM | - | - | - |
|  | Lasso | 0.410 (0.790) | 0.790 | 0.800 |
|  | DML1 | 0.530 (0.840) | 0.970 | 0.870 |
|  | DML2 | 0.520 (0.830) | 0.960 | 0.880 |
|  | TMLE | 0.520 (0.920) | 1.120 | 0.960 |
|  | l_1_-TCL | - | - | - |
|  | DSTCL | 0.070 (0.250) | 0.070 | 0.960 |

“–” indicates that the corresponding result was unavailable. GLM was not estimable in these settings; for l_1_-TCL, it indicates that the simulation procedure was not successfully completed in all replications.

## Additional simulation sensitivity analyses

**Table D. Regularization sensitivity analysis for DSTCL.**

| **Scenario** | **Tuning rule** | **Bias (SD)** | **MSE** | **Coverage** |
| --- | --- | --- | --- | --- |
| Both PS and OR correct | lambda.1se | 0.050 (0.260) | 0.070 | 0.950 |
| Both PS and OR correct | lambda.min | 0.060 (0.240) | 0.060 | 0.940 |
| PS correct only | lambda.1se | 0.040 (0.230) | 0.060 | 0.960 |
| PS correct only | lambda.min | 0.120 (0.240) | 0.070 | 0.900 |
| OR correct only | lambda.1se | 0.070 (0.250) | 0.070 | 0.960 |
| OR correct only | lambda.min | 0.070 (0.240) | 0.060 | 0.960 |

**Table E. Sensitivity analysis under increasingly extreme true propensity scores.**

**(a) True propensity-score diagnostics averaged over 200 replications**

| **Scenario** | **PS strength** | **Domain** | **Mean min** | **Mean p05** | **Mean median** | **Mean p95** | **Mean max** | **Mean prop. <0.05** | **Mean prop. >0.95** | **Mean ESS** |
| --- | --- | --- | --- | --- | --- | --- | --- | --- | --- | --- |
| Both PS and OR correct | 2 | Source | 0.00000 | 0.0011 | 0.598 | 0.9995 | 1.00000 | 0.227 | 0.284 | 127.24 |
| Both PS and OR correct | 2 | Target | 0.00019 | 0.0014 | 0.418 | 0.9968 | 0.99957 | 0.283 | 0.224 | 24.12 |
| Both PS and OR correct | 3 | Source | 0.00000 | 0.0000 | 0.648 | 1.0000 | 1.00000 | 0.297 | 0.364 | 140.98 |
| Both PS and OR correct | 3 | Target | 0.00000 | 0.0001 | 0.400 | 0.9998 | 0.99999 | 0.357 | 0.305 | 26.62 |
| PS correct only | 2 | Source | 0.00000 | 0.0011 | 0.598 | 0.9995 | 1.00000 | 0.227 | 0.284 | 127.24 |
| PS correct only | 2 | Target | 0.00019 | 0.0014 | 0.418 | 0.9968 | 0.99957 | 0.283 | 0.224 | 24.12 |
| PS correct only | 3 | Source | 0.00000 | 0.0000 | 0.648 | 1.0000 | 1.00000 | 0.297 | 0.364 | 140.98 |
| PS correct only | 3 | Target | 0.00000 | 0.0001 | 0.400 | 0.9998 | 0.99999 | 0.357 | 0.305 | 26.62 |
| OR correct only | 2 | Source | 0.00000 | 0.0010 | 0.603 | 0.9995 | 1.00000 | 0.228 | 0.287 | 131.96 |
| OR correct only | 2 | Target | 0.00018 | 0.0016 | 0.417 | 0.9972 | 0.99972 | 0.278 | 0.226 | 23.10 |
| OR correct only | 3 | Source | 0.00000 | 0.0000 | 0.645 | 1.0000 | 1.00000 | 0.300 | 0.364 | 135.97 |
| OR correct only | 3 | Target | 0.00000 | 0.0002 | 0.377 | 0.9997 | 0.99998 | 0.361 | 0.297 | 28.21 |

**(b) Estimator performance**

| **Scenario** | **PS strength** | **Bias (SD)** | **MSE** | **Coverage** |
| --- | --- | --- | --- | --- |
| Both PS and OR correct | 2 | 0.130 (0.380) | 0.160 | 0.940 |
| Both PS and OR correct | 3 | 0.110 (0.320) | 0.110 | 0.960 |
| PS correct only | 2 | 0.200 (0.380) | 0.190 | 0.890 |
| PS correct only | 3 | 0.170 (0.300) | 0.120 | 0.920 |
| OR correct only | 2 | 0.080 (0.290) | 0.090 | 0.980 |
| OR correct only | 3 | 0.180 (0.380) | 0.170 | 0.970 |

The PS-strength parameter scales the linear predictor in the data-generating treatment model. Larger values create probabilities closer to 0 or 1; it is a simulation control parameter rather than an estimated model parameter.

**Table F. Trimming sensitivity analysis for DSTCL.**

| **Scenario** | **PS strength** | **Trimming interval** | **Bias (SD)** | **MSE** | **Coverage** |
| --- | --- | --- | --- | --- | --- |
| Both PS and OR correct | 1 | No trimming | 0.060 (0.260) | 0.070 | 0.940 |
| Both PS and OR correct | 1 | [0.01, 0.99] | 0.060 (0.260) | 0.070 | 0.940 |
| Both PS and OR correct | 1 | [0.05, 0.95] | 0.060 (0.260) | 0.070 | 0.940 |
| Both PS and OR correct | 2 | No trimming | 0.140 (0.280) | 0.100 | 0.950 |
| Both PS and OR correct | 2 | [0.01, 0.99] | 0.140 (0.280) | 0.100 | 0.950 |
| Both PS and OR correct | 2 | [0.05, 0.95] | 0.130 (0.280) | 0.090 | 0.920 |
| PS correct only | 1 | No trimming | 0.110 (0.260) | 0.080 | 0.920 |
| PS correct only | 1 | [0.01, 0.99] | 0.110 (0.260) | 0.080 | 0.920 |
| PS correct only | 1 | [0.05, 0.95] | 0.110 (0.260) | 0.080 | 0.920 |
| PS correct only | 2 | No trimming | 0.210 (0.280) | 0.120 | 0.910 |
| PS correct only | 2 | [0.01, 0.99] | 0.210 (0.280) | 0.120 | 0.900 |
| PS correct only | 2 | [0.05, 0.95] | 0.200 (0.270) | 0.110 | 0.880 |
| OR correct only | 1 | No trimming | 0.040 (0.230) | 0.050 | 0.980 |
| OR correct only | 1 | [0.01, 0.99] | 0.040 (0.230) | 0.050 | 0.980 |
| OR correct only | 1 | [0.05, 0.95] | 0.040 (0.230) | 0.050 | 0.980 |
| OR correct only | 2 | No trimming | 0.120 (0.310) | 0.110 | 0.950 |
| OR correct only | 2 | [0.01, 0.99] | 0.120 (0.310) | 0.110 | 0.950 |
| OR correct only | 2 | [0.05, 0.95] | 0.130 (0.280) | 0.100 | 0.940 |

**Table G. Covariate-shift sensitivity analysis for DSTCL.**

**(a) Realized target-source shift and target-domain PS diagnostics**

| **Scenario** | **Rep.** | **Mean source X** | **Mean target X** | **Mean difference** | **Mean prop. PS <0.05** | **Mean prop. PS >0.95** | **Mean ESS** |
| --- | --- | --- | --- | --- | --- | --- | --- |
| Both PS and OR correct | 200 | 0.0000 | 0.0505 | 0.0505 | 0.033 | 0.201 | 24.49 |
| PS correct only | 200 | 0.0000 | 0.0505 | 0.0505 | 0.033 | 0.201 | 24.49 |
| OR correct only | 200 | 0.0001 | 0.0504 | 0.0504 | 0.035 | 0.202 | 26.89 |

**(b) DSTCL performance**

| **Scenario** | **Bias (SD)** | **MSE** | **Coverage** |
| --- | --- | --- | --- |
| Both PS and OR correct | 0.100 (0.280) | 0.090 | 0.960 |
| PS correct only | 0.150 (0.280) | 0.100 | 0.920 |
| OR correct only | 0.050 (0.280) | 0.080 | 0.960 |

The source-domain covariate mean was set to 0 and the target-domain mean was shifted by 0.05 for each non-intercept covariate.

**Table H. Computational time and convergence diagnostics for DSTCL as the covariate dimension increases.**

| **Scenario** | **p** | **Completed replications** | **DSTCL failures** | **Mean main-fit time (sec)** | **Mean time per bootstrap fit (sec)** |
| --- | --- | --- | --- | --- | --- |
| Both PS and OR correct | 50 | 200 | 0 | 1.034 | 1.398 |
| Both PS and OR correct | 100 | 200 | 0 | 1.287 | 1.896 |
| Both PS and OR correct | 200 | 200 | 0 | 1.714 | 13.962 |
| PS correct only | 50 | 200 | 0 | 1.412 | 1.405 |
| PS correct only | 100 | 200 | 0 | 0.856 | 1.034 |
| PS correct only | 200 | 200 | 0 | 2.005 | 15.251 |
| OR correct only | 50 | 200 | 0 | 1.007 | 1.308 |
| OR correct only | 100 | 200 | 0 | 1.321 | 1.851 |
| OR correct only | 200 | 200 | 0 | 1.893 | 16.037 |

# Real-data supplementary analyses

**Table I. Variable-level source-target distribution diagnostics.**

| **Variable** | **Source mean/prop.** | **Target mean/prop.** | **SMD** | **\|SMD\|** | **Wilcoxon/categorical P** |
| --- | --- | --- | --- | --- | --- |
| ABeta42 status | 0.4082 | 0.5306 | 0.245 | 0.245 | 0.1191 |
| Sex | 0.4320 | 0.6327 | 0.402 | 0.402 | 0.0086 |
| Education | 16.0721 | 15.5510 | -0.190 | 0.190 | 0.1888 |
| age | 62.4877 | 62.8163 | 0.046 | 0.046 | 0.7444 |
| cg25818615 | 0.8724 | 0.8723 | -0.002 | 0.002 | 0.9054 |
| cg03565013 | 0.8339 | 0.8277 | -0.057 | 0.057 | 0.2174 |
| cg04200627 | 0.8915 | 0.8975 | 0.404 | 0.404 | 0.0029 |
| cg21464891 | 0.8879 | 0.8847 | -0.036 | 0.036 | 0.2017 |
| cg06201680 | 0.0891 | 0.0917 | 0.076 | 0.076 | 0.3051 |
| cg23605175 | 0.9141 | 0.9183 | 0.264 | 0.264 | 0.0130 |
| cg26172387 | 0.9016 | 0.9053 | 0.247 | 0.247 | 0.0980 |
| cg25250374 | 0.7825 | 0.7852 | 0.051 | 0.051 | 0.7051 |
| cg26369996 | 0.9018 | 0.9039 | 0.149 | 0.149 | 0.6943 |
| cg03159926 | 0.8821 | 0.8828 | 0.046 | 0.046 | 0.9488 |
| cg15653569 | 0.8554 | 0.8551 | -0.014 | 0.014 | 0.6936 |
| cg03626739 | 0.1771 | 0.1768 | -0.008 | 0.008 | 0.9757 |
| cg17332016 | 0.1100 | 0.1072 | -0.201 | 0.201 | 0.0313 |
| cg11944093 | 0.8856 | 0.8890 | 0.151 | 0.151 | 0.3675 |
| cg10493585 | 0.8892 | 0.8937 | 0.220 | 0.220 | 0.0538 |
| cg08967134 | 0.8885 | 0.8895 | 0.055 | 0.055 | 0.7480 |
| cg07542043 | 0.8389 | 0.8315 | -0.319 | 0.319 | 0.0444 |
| cg04426031 | 0.8817 | 0.8866 | 0.215 | 0.215 | 0.1195 |
| cg15346359 | 0.9014 | 0.8939 | -0.198 | 0.198 | 0.4284 |
| cg10455672 | 0.7640 | 0.7665 | 0.094 | 0.094 | 0.7344 |
| cg05714838 | 0.8262 | 0.8231 | -0.056 | 0.056 | 0.6791 |
| cg13076468 | 0.9029 | 0.9034 | 0.031 | 0.031 | 0.6806 |
| cg00396899 | 0.7313 | 0.7480 | 0.196 | 0.196 | 0.3108 |
| cg23384045 | 0.8124 | 0.8124 | 0.004 | 0.004 | 0.5683 |
| cg20208633 | 0.2713 | 0.2733 | 0.022 | 0.022 | 0.9898 |
| cg07018739 | 0.8734 | 0.8717 | -0.102 | 0.102 | 0.5497 |
| cg03778029 | 0.1852 | 0.1714 | -0.328 | 0.328 | 0.0741 |
| cg00095431 | 0.8439 | 0.8454 | 0.050 | 0.050 | 0.2305 |
| cg02562299 | 0.8854 | 0.8981 | 0.248 | 0.248 | 0.9466 |
| cg19772847 | 0.8415 | 0.8145 | -0.356 | 0.356 | 0.0104 |
| cg06584030 | 0.1161 | 0.1144 | -0.137 | 0.137 | 0.3236 |
| cg14143728 | 0.8837 | 0.8849 | 0.084 | 0.084 | 0.7575 |
| cg03328984 | 0.8200 | 0.8129 | -0.109 | 0.109 | 0.1438 |
| cg22402154 | 0.8120 | 0.8139 | 0.030 | 0.030 | 0.8246 |
| cg07721983 | 0.8764 | 0.8775 | 0.074 | 0.074 | 0.7027 |
| cg03801758 | 0.8584 | 0.8621 | 0.252 | 0.252 | 0.0408 |
| cg05745971 | 0.8078 | 0.8092 | 0.042 | 0.042 | 0.8065 |
| cg09869112 | 0.8527 | 0.8506 | -0.121 | 0.121 | 0.3903 |
| cg02348989 | 0.8987 | 0.8983 | -0.020 | 0.020 | 0.4184 |
| cg08155817 | 0.1351 | 0.1377 | 0.168 | 0.168 | 0.2395 |
| cg21904271 | 0.8540 | 0.8538 | -0.003 | 0.003 | 0.6062 |
| cg04933176 | 0.7194 | 0.7134 | -0.066 | 0.066 | 0.5520 |
| cg09354295 | 0.6993 | 0.6996 | 0.008 | 0.008 | 0.8588 |
| cg02830555 | 0.7981 | 0.8110 | 0.233 | 0.233 | 0.3728 |
| cg10260572 | 0.8750 | 0.8821 | 0.209 | 0.209 | 0.3703 |
| cg05880455 | 0.8829 | 0.8825 | -0.017 | 0.017 | 0.9827 |
| cg01758743 | 0.9145 | 0.9178 | 0.186 | 0.186 | 0.0470 |
| cg13109095 | 0.7726 | 0.7762 | 0.070 | 0.070 | 0.8885 |
| cg01195881 | 0.9049 | 0.9114 | 0.172 | 0.172 | 0.1865 |
| cg02552490 | 0.8506 | 0.8287 | -0.180 | 0.180 | 0.8541 |
| cg02564761 | 0.2474 | 0.2513 | 0.054 | 0.054 | 0.9895 |
| cg10478549 | 0.7521 | 0.7497 | -0.032 | 0.032 | 0.7030 |
| cg07482257 | 0.6338 | 0.6173 | -0.166 | 0.166 | 0.3654 |
| cg15664161 | 0.8816 | 0.8982 | 0.399 | 0.399 | 0.0184 |
| cg20210263 | 0.8778 | 0.8746 | -0.154 | 0.154 | 0.3090 |
| cg20901246 | 0.1619 | 0.1546 | -0.262 | 0.262 | 0.0658 |
| cg26372998 | 0.8010 | 0.7992 | -0.040 | 0.040 | 0.5393 |
| cg07259651 | 0.9316 | 0.9299 | -0.206 | 0.206 | 0.1547 |
| cg19255828 | 0.1580 | 0.1558 | -0.059 | 0.059 | 0.7580 |
| cg12044973 | 0.8320 | 0.8325 | 0.019 | 0.019 | 0.4292 |
| cg14235674 | 0.2954 | 0.3147 | 0.320 | 0.320 | 0.0061 |
| cg14365043 | 0.5828 | 0.5801 | -0.048 | 0.048 | 0.9339 |
| cg06562865 | 0.1947 | 0.2056 | 0.254 | 0.254 | 0.0540 |
| cg17318529 | 0.1774 | 0.1860 | 0.219 | 0.219 | 0.2821 |
| cg25328154 | 0.1455 | 0.1569 | 0.235 | 0.235 | 0.0632 |
| cg12974637 | 0.1970 | 0.1945 | -0.045 | 0.045 | 0.9968 |
| cg05605414 | 0.8925 | 0.8931 | 0.049 | 0.049 | 0.9392 |
| cg02178763 | 0.1963 | 0.1857 | -0.141 | 0.141 | 0.4469 |
| cg11399192 | 0.8486 | 0.8441 | -0.190 | 0.190 | 0.0566 |
| cg22429640 | 0.5497 | 0.5683 | 0.168 | 0.168 | 0.3783 |
| cg12796873 | 0.8202 | 0.8137 | -0.266 | 0.266 | 0.0446 |
| cg05602920 | 0.8754 | 0.8763 | 0.049 | 0.049 | 0.9776 |
| cg00382930 | 0.7740 | 0.7695 | -0.126 | 0.126 | 0.5994 |
| cg24469729 | 0.7546 | 0.7489 | -0.178 | 0.178 | 0.2269 |
| cg14825116 | 0.9428 | 0.9333 | -0.274 | 0.274 | 0.1530 |
| cg20741319 | 0.8878 | 0.8783 | -0.258 | 0.258 | 0.0425 |
| cg13296755 | 0.9023 | 0.8984 | -0.262 | 0.262 | 0.0775 |
| cg27306191 | 0.7935 | 0.7890 | -0.094 | 0.094 | 0.4000 |
| cg02539882 | 0.8808 | 0.8809 | 0.010 | 0.010 | 0.7076 |
| cg14520626 | 0.1253 | 0.1339 | 0.207 | 0.207 | 0.0291 |
| cg08339744 | 0.8930 | 0.8941 | 0.056 | 0.056 | 0.9021 |
| cg00267207 | 0.7709 | 0.7891 | 0.135 | 0.135 | 0.1588 |
| cg06522703 | 0.9083 | 0.9137 | 0.319 | 0.319 | 0.0329 |
| cg16390418 | 0.9132 | 0.9087 | -0.075 | 0.075 | 0.6435 |
| cg05499127 | 0.6097 | 0.6138 | 0.051 | 0.051 | 0.8906 |
| cg10263181 | 0.8422 | 0.8402 | -0.043 | 0.043 | 0.3854 |
| cg18302890 | 0.7580 | 0.7682 | 0.131 | 0.131 | 0.2525 |
| cg09850632 | 0.2748 | 0.2660 | -0.149 | 0.149 | 0.4752 |
| cg12979921 | 0.8774 | 0.8764 | -0.039 | 0.039 | 0.8334 |
| cg02446366 | 0.1952 | 0.1853 | -0.200 | 0.200 | 0.0350 |
| cg02230692 | 0.9062 | 0.9069 | 0.061 | 0.061 | 0.9745 |
| cg00082732 | 0.7630 | 0.7519 | -0.184 | 0.184 | 0.1066 |
| cg05214708 | 0.8262 | 0.8275 | 0.050 | 0.050 | 0.4937 |
| cg26022717 | 0.8983 | 0.8968 | -0.076 | 0.076 | 0.2189 |
| cg12551751 | 0.8167 | 0.8232 | 0.215 | 0.215 | 0.1191 |
| cg11301372 | 0.8260 | 0.8270 | 0.029 | 0.029 | 0.5545 |
| cg26286043 | 0.6693 | 0.6588 | -0.135 | 0.135 | 0.4145 |
| cg03134408 | 0.8043 | 0.7995 | -0.198 | 0.198 | 0.1343 |
| cg25181751 | 0.2341 | 0.2395 | 0.068 | 0.068 | 0.6099 |
| cg03377871 | 0.8506 | 0.8433 | -0.165 | 0.165 | 0.0502 |

Standardized mean differences were used as the primary measures of source-target covariate comparability. Wilcoxon rank-sum tests were used for continuous variables, whereas chi-square or Fisher’s exact tests were used for categorical variables.

**Table J. Propensity-score and effective-sample-size diagnostics in the ADNI analyses.**

| **Estimator** | **PS min** | **PS p05** | **PS median** | **PS p95** | **PS max** | **ESS exposed** | **ESS unexposed** | **ESS total** |
| --- | --- | --- | --- | --- | --- | --- | --- | --- |
| l1-TCL | 0.002 | 0.060 | 0.397 | 0.745 | 0.904 | 17.44 | 20.59 | 33.34 |
| DSTCL | 0.300 | 0.337 | 0.409 | 0.484 | 0.521 | 25.58 | 22.87 | 46.78 |

The DSTCL propensity scores ranged from 0.300 to 0.521, and its total effective sample size was 46.78 relative to 49 target participants.

**Table K. DSTCL estimates from the ADNI imputation analyses.**

| **Analysis** | **Estimate** | **Percentile 95% CI** |
| --- | --- | --- |
| Primary | -6.333 | (-9.585, -2.475) |
| Median/mode sensitivity | -7.884 | (-18.008, -3.563) |

The simple-imputation sensitivity analysis used median imputation for continuous variables and mode imputation for categorical variables. The latter retained the direction of the DSTCL estimate but produced substantially greater bootstrap variability.
