# Supplementary material for: Manifold-constrained plasticity enables stable learning in recurrent neural circuits
Source: PLoS Comput Biol. 2026 Aug 25;22(8):e1014719. doi: 10.1371/journal.pcbi.1014719 (PMC13529248; doi:10.1371/journal.pcbi.1014719)
Supplement: Supplementary file 2 [file pcbi.1014719.s016.docx]

In this study, the authors provide a computational account of how stable learning may take place even in the face of representational drift. Through their proposed plasticity rule called SPLiT (Synaptic Projection Learning with intrinsic Tracking), they demonstrate how it may be beneficial to simultaneously track the evolving neural manifold during supervised learning, to allow for faster learning and robustness to noise. The SPLiT learning rule has two components: (1) an unsupervised Oja rule to track the manifold in an online manner and (2) a Least Mean Square (LMS) algorithm to train the readout weights of the recurrent neural network (RNN) based on the manifold coordinates.

The paper is well structured, and I thank the authors for their clarity of writing. I really liked how the paper aligns with the findings from the seminal BCI study by Sadtler et al. (2014), which highlighted important differences between learning within and outside manifold. This empirical connection, together with their results demonstrating the possible advantages of manifold-constrained learning using the SPLiT rule, are in my view the study’s strongest contributions.

As the authors themselves emphasize, the paper’s aim is not to provide a biologically plausible account of how the manifold may be tracked or how the readout weights may be trained, but instead to understand at a computational level how learning may adapt to representational drift. I understand the authors’ viewpoint, but I do believe that this paper would benefit from a more thorough treatment of the following points:

Major comments:

- Although SPLiT may be an abstraction and it is acknowledged that an explicit projection may not actually take place (and only allows here for a controlled experimental setting), it is not clear what possible underlying biology would be consistent with the current framework. Here, the projected activity y(t) does not map onto any set of neurons and lives abstractly somewhere between the activity r(t) and the network output z(t). Are the authors suggesting that this low-dimensional structure would live in the activity r(t) itself? If so, would it need to be tracked by a subset of the neurons, or estimated by another network? The Oja rule itself is considered biologically plausible (ignoring the normalization), but I am not entirely sure how it would be implemented here. How would the decoder/readout gain explicit access to the low-dimensional structure? Given how central this point is to the paper’s main arguments, I would be grateful if the authors could shed further light on this, either through toy experiments or via a deeper conceptual exposition.
- The abstract, introduction, discussion and methods sections are well structured and easy to read, but I wonder whether the results section could be finessed through more informative sub-headings that convey the main message of the relevant section, e.g., “manifold-constrained learning confers robustness to noise” instead of “learning noise target signals”. The flow is not entirely clear from the sub-headings, and I feel that the results section could be tightened in general.
- The comparison between SPLiT and FORCE in the constrained case seems somewhat contrived and not fully justified to me. FORCE will fail by design given that it is implicitly inverting the full covariance matrix. Is the comparison to FORCE really necessary here, or could a comparison be made for instance using a learning algorithm that does not involve the full covariance matrix?
- More generally, I am not entirely sure how to view comparisons with related approaches in the constrained scenario (where other approaches underperform since they do not estimate the low-dimensional structure). I think this probably links back to my first comment (and can be answered together), but is this comparison well-defined biologically? The constrained regime is simply one where neural activity remains confined to a lower-dimensional subspace, but under what circumstances would downstream readout neurons have explicit access to the low dimensional structure?

Minor comments:

- In Fig 6C, the authors argue that the error increases as the dimensionality (k) is increased. It seems that the MSE consistently decreases again above k~40. Could the authors extend k to higher values? Is this being run with several seeds?
- Could the authors expand on the main contributing factor here to drift in the empirical covariance structure? Is my understanding correct that drift is caused primarily by the change in readout weights D(t) through learning, which changes learning feedback input I_fb (t)? Does the noise term also potentially contribute?
- Does the drift noise \eta_r (listed in Table 1. Default model parameters) enter any relevant equations? Where is the drift noise term used?
- Would it make more sense to use y(t) or f(t) as the network output, instead of z(t)? I am more familiar with y(t) as output, but have no problem with the current notation if this tends to be the convention in this specific area.
- The authors consider here a reservoir computing setting, where the input weights and recurrent weights are fixed. It is however also possible for the input weights and recurrent weights to be learned through algorithms such as e-prop/RFLO. Would this framework also easily extend to such a scenario?

References:

Bellec, G., Scherr, F., Subramoney, A., Hajek, E., Salaj, D., Legenstein, R., & Maass, W. (2020). A solution to the learning dilemma for recurrent networks of spiking neurons. *Nature communications*, *11*(1), 3625.

Murray, J.M., 2019. Local online learning in recurrent networks with random feedback. *Elife*, *8*, p.e43299.
