# Supplementary material for: Manifold-constrained plasticity enables stable learning in recurrent neural circuits
Source: PLoS Comput Biol. 2026 Aug 25;22(8):e1014719. doi: 10.1371/journal.pcbi.1014719 (PMC13529248; doi:10.1371/journal.pcbi.1014719)
Supplement: Supplementary file 4 [file pcbi.1014719.s018.docx]

**Reviewer #1:** 
This manuscript introduces a learning rule for reservoir networks that separates two distinct processes: tracking changes of a low-dimensional activity manifold and learning on that drifting manifold. The approach is conceptually interesting and the results appear promising. I believe this is valuable work; however, there are several points that would benefit from clarification before publication. My comments fall into three categories: conceptual issues that may require additional analyses, presentation issues that currently make the manuscript difficult to follow, and minor points.

Conceptual issues

- Manifold rotation as the dominant form of drift. A growing body of evidence suggests that the dominant form of representational drift in neural circuits is a rotation of the population activity manifold, rather than a remapping that keeps activity confined to the same manifold (e.g. Sylte et al. 2025; recent review by Eppler et al. 2025, and references therein; see also Ziv et al. 2013, where this effect is visible in the figures although not emphasized). During drift, a substantial fraction of neurons drop in and out of the active population, which appears more consistent with a rotation of the entire manifold than with activity remaining on a fixed one. This is precisely the regime in which, based on the results presented, the proposed algorithm seems to perform least well. I would therefore encourage the authors to include an analysis in which drift is implemented as a sequence of small, incremental rotations, and to benchmark SPLiT against FORCE and the other algorithms compared in Figure 8. How does performance degrade (or not) as the number and magnitude of rotations vary?

We thank the reviewer for this important suggestion. We agree that recent evidence increasingly supports representational drift as a gradual rotation of low-dimensional population structure rather than abrupt remapping. To address this point, we added new analyses in which the manifold evolves through incremental rotations. These analyses are presented in a new section titled “Online subspace tracking stabilizes learning under rotational drift” (lines 623+):

Simulations were performed with a gradual rotational drift (Suppl. Fig S8A). The manifold basis evolves as

$$Q\left( t+\Delta t \right)=R\left( \Delta\theta_{t} \right)Q(t)$$

(34)

where $Q\left( t \right)\in\mathbb{R}^{N\times d}$ is the orthonormal basis spanning the low-dimensional manifold at time $t$, $\Delta t$ is the simulation time step, $R\left( \Delta\theta_{t} \right)\in\mathbb{R}^{d\times d}$ is a rotation matrix, and $\Delta\theta_{t}$ is the incremental rotation angle. Under rotational drift, decoder performance depended on the strength of feedback (Suppl. Fig S8B,C). This suggests that rotational drift relies on stabilizing feedback, allowing the decoder to remain aligned with the evolving population subspace. FORCE learning (Suppl. Fig S8B) and alternative models did not perform accurately on this task (Suppl Fig S9).

Next, we extended these results to the center-out reaching task with rotational perturbations. For all models except Bayesian inference, within-manifold perturbations yielded worst performance than between-manifold perturbations (Suppl. Fig S10). This result differs from SPLiT, where performance was more sensitive to between- than within-manifold perturbations (Fig 3D), suggesting that tracking of the intrinsic low-dimensional activity subspace is critical for preserving decoder alignment under manifold drift.

To further establish whether online subspace tracking was required under rotational drift, simulations were performed where the manifold basis $U\left( t \right)$ remained frozen once initialized. In these simulations, the frozen basis remained poorly aligned with the evolving population subspace (Suppl. Fig S11A). By contrast, allowing $U\left( t \right)$ to adapt over training improved the alignment of $U\left( t \right)$ with the evolving manifold. This improved alignment was accompanied by a lower decoding error (Fig S11B,C). Thus, under manifold drift, online subspace tracking contributes to maintaining decoder alignment and improving task performance.

Finally, simulations were performed to examine whether SPLiT could recover from a rotational perturbation introduced after training on the sine wave task. Small rotations produced little disruption to performance, whereas larger rotations caused progressively larger increases in error (Suppl. Fig S12). Even for large rotations, however, SPLiT maintained lower error than simulations where the manifold basis $U\left( t \right)$ remained frozen, indicating that online subspace tracking improves decoding performance across a range of perturbation strengths.

- Task generality. The temporal tracking task used as the primary benchmark is relatively abstract. It would be helpful to see the reaching task evaluated more prominently. In particular, I encourage the authors to include a comparison analogous to Figure 8 using the reaching task instead of the temporal tracking task. Ideally, this could also be evaluated on a rotated manifold, to assess performance under a biologically plausible form of drift with a non-stationary target manifold.

We added a comparison of various models on the reaching task (Suppl. Fig. S10), with accompanying text (lines 631+):

Next, we extended these results to the center-out reaching task with rotational perturbations. For all models except Bayesian inference, within-manifold perturbations yielded worst performance than between-manifold perturbations (Suppl. Fig S10). This result differs from SPLiT, where performance was more sensitive to between- than within-manifold perturbations (Fig 3D), suggesting that tracking of the intrinsic low-dimensional activity subspace is critical for preserving decoder alignment under manifold drift.

- Normalization and gain. The manuscript states that the network is normalized to the largest eigenvalue, while also treating gain as a free parameter. As written, I found this difficult to reconcile: if the gain is modified and the network is subsequently renormalized to a largest eigenvalue of 1, the gain change would appear to have no lasting effect. I would appreciate clarification of the order of operations and why both procedures are necessary, or alternatively a simplification of the parameterization to ensure internal consistency.

We removed Eq.4 as it was obsolete (part of an earlier version of the model). This allows simulations to isolate the role of recurrent gain. The correct version of the code has been uploaded along with the manuscript.

Presentation issues

- Description of the method in the Results. The manuscript introduces SPLiT in the Results section but would benefit from a more intuitive or qualitative description of the method at that stage. In particular, it would help to briefly outline what the rule does, which variables it modifies, and what behavior it is expected to induce in the network. As it stands, it is difficult to interpret the reported results or follow the logic of the early figures without repeatedly consulting the Methods. I recommend including a concise, high-level description of the learning rule in the Results section, covering at minimum the type of network used, the mechanism of SPLiT, how training proceeds, and how dimensionality is constrained, before presenting results. A similar point applies to the handwritten digit task: a brief description in the main text before or alongside the results would substantially improve readability.

We moved the Methods section into Results. We also added a brief description of the handwritten digit task (lines 343+):

Individual MNIST digits were first reduced to a one-pixel-wide representation preserving their overall shape and topology. The resulting coordinates were then converted to a continuous two-dimensional trajectory of 1,000 data points that evolved over time (one point per time step). The decoder was trained using SPLiT to reproduce these trajectories.

- Motivation for FORCE's behavior at low dimensionality. On p. 3 (l. 82) and p. 4 (l. 141), the text alludes to a specific reason why FORCE performs poorly in low-dimensional settings, but the explanation is deferred to the Discussion. Providing at least some intuition in the Results section would help the reader follow the narrative without needing to skip ahead.

We thank the reviewer for this suggestion. We now provide an intuitive explanation when discussing Fig.2C (lines 249+):

Intuitively, FORCE assumes that neural activity spans a sufficiently rich high-dimensional space such that small changes in decoder weights can reconstruct the target signal. However, when activity is restricted to a low-dimensional manifold, many directions in neuron space become unavailable, causing the covariance structure of neural activity to become low-rank. In this regime, FORCE becomes ill-conditioned because the decoder attempts to invert activity patterns that no longer contain independent dimensions. This instability arises because FORCE distributes decoder updates across directions that are poorly aligned with the intrinsic manifold. In contrast, SPLiT continuously aligns decoder updates with the dominant low-dimensional activity subspace (Fig 2D). Although FORCE could in principle be modified through a pseudoinverse formulation, or by rewriting the learning rule in intrinsic manifold coordinates, such approaches assume prior access to the manifold structure itself. In contrast, SPLiT provides a synaptic mechanism that continuously estimates and tracks the evolving activity subspace during learning, thereby constraining decoder adaptation to directions supported by the intrinsic population dynamics.

Minor points

- Discussion of drift mechanisms. Recent theoretical work suggests that representational drift may in part reflect ongoing learning or synaptic plasticity (Qin et al. 2023; Ratzon et al. 2024; Devalle et al. 2025; Eppler et al. 2026). The Discussion would benefit from engaging more directly with this literature. In particular, how does ongoing learning interact with manifold stability at the representational level? Does the network output also drift, and if so, is ongoing plasticity in the readout population the relevant mechanism?

We agree that the Discussion did not sufficiently address recent theoretical work proposing that representational drift may reflect ongoing learning and synaptic plasticity. To address this, we added a new section termed “Neural basis of representational drift” (lines 726+) discussing how continual plasticity within recurrent circuits can drive manifold drift while downstream readout plasticity compensates to preserve stable task output:

Recent theoretical work suggests that representational drift may not simply reflect noise, instability, or degradation of neural coding, but instead arise from ongoing synaptic plasticity within recurrent circuits [26,31,33,40]. In this perspective, neuronal populations remain plastic even after behavioral performance has stabilized, causing internal representations to reorganize over time while preserving task-relevant computations. Representational drift therefore reflects an active process of circuit remodeling rather than a passive failure of neural stability mechanisms.

Consistent with this view, Qin et al. [70] proposed that drift can emerge from noisy Hebbian learning in networks with multiple equivalent representational solutions, producing coordinated manifold rotations while preserving population-level structure. Similarly, Ratzon et al. [71] argued that drift may reflect a slow process of regularization in continually trained networks, where representations gradually reorganize toward sparser solutions despite stable task performance. Devalle et al. [72] further suggested that drift may be an unavoidable consequence of ongoing memory storage, whereby continual synaptic overwriting progressively decorrelates activity patterns. More recently, Eppler et al. [73] demonstrated that representational drift may emerge from the interaction between stochastic synaptic fluctuations and compensatory Hebbian plasticity. Together, these studies support the view that representational drift reflects an intrinsic property of adaptive neural systems undergoing continual synaptic turnover rather than a failure of neural coding. This view is consistent with experimental findings showing stable behavior despite substantial synaptic turnover [36,42–45]. An implication of these findings is that learned behavior does not require stable activity at the level of individual neurons but may instead be preserved through coordinated population dynamics combined with compensatory plasticity.

SPLiT is consistent with this interpretation of representational drift. In our framework, drift emerges because the structure of recurrent population activity evolves over time, causing the underlying manifold coordinates to reorganize. Drift can therefore occur both within the recurrent population manifold itself and indirectly at the level of decoder outputs if readout weights fail to track the evolving latent structure. Stable performance thus requires the continual adaptation of downstream readout weights to maintain alignment with ongoing changes in neural population structure. In this view, manifold stability should not be viewed as a static property of recurrent activity, but rather as a dynamic equilibrium emerging from the interaction between recurrent drift and continual plasticity in downstream readout populations.

This interpretation also provides a possible explanation for why neural manifolds often appear stable over short timescales yet gradually reorganize over longer periods [11,22]. Fast compensatory plasticity may preserve enough task-relevant structure to maintain behavior, while slower synaptic turnover and learning processes progressively reshape the underlying population structure. From this perspective, representational drift and behavioral stability are not contradictory phenomena, but reflect consequences of continual learning in neuronal circuits.

- Principal angle metric (p. 4, l. 135; Fig. 2). It would be helpful to clarify whether a mean principal angle near zero is a meaningful outcome under this definition, or whether random, uncorrelated angles would also average to approximately zero. If the latter, the metric may not distinguish dynamics in the intended way.

We thank the reviewer for this clarification. We agree that the interpretation of the principal angle metric should be stated more explicitly. In the revised manuscript, we now clarify as follows (lines 241+):

Before examining the performance of SPLiT on this task, we verified that in a scenario where an RNN was designed with constrained activity (Eqs. 1-4), dynamics remained confined to a fixed manifold. This was quantified by computing the mean principal angle (Methods, Eqs. 35-37) between the initial manifold and the instantaneous activity subspace over time. Under constrained dynamics, this angle remained close to zero (Fig 2A-B), indicating strong alignment between the two subspaces. Thus, constrained RNN activity remained within a fixed manifold determined at the onset of training.

- Figure 2. The left (panels A-C) and right (panels D-F) parts could be labeled explicitly as “constrained” and “unconstrained” (e.g., as titles or annotations) to make the comparison immediately clear.

Ok done.

- Figure 4. A legend should be added to the MNIST tracking panel, as it is currently unclear what the colors represent.

Ok we added a colorbar to figures 4 and 6.

- Use of λ. The symbol λ is used both for the learning rate and for eigenvalues. Distinct notation would improve clarity.

Ok we changed λ to $\eta_{D}$ throughout to reflect the learning rate of the decoder.

- Figures 5, 6, and 7. These figures compare the constrained and unconstrained versions of SPLiT but do not include FORCE. As FORCE is a standard baseline, including it would strengthen the comparison.

Ok we added FORCE to Figures 5,6,7.

- Figure 6A. A colorbar appears to be missing and should be added.

Ok added.

- Figure 6C. At certain dimensionalities, the MSE decreases again after an initial rise. This non-monotonic pattern is unexpected and currently unexplained. It would be helpful to either discuss the underlying mechanism or clarify whether this might reflect an artifact.

Yes, it was an artefact. We re-ran with different initial seeds and this effect goes away. We clarified the figure legend accordingly:

Panels B-C are means over 10 independent realizations with random initial seeds.

- Figure 7A. A legend is missing and should be added.

Ok added.

- Methods: initialization of W. The initialization procedure is described in a way that seems more complex than necessary. If lower-rank matrices cannot be generated directly, it would be helpful to briefly explain why the current approach is required.

Eq.4 was not obsolete in the model and was not employed in simulations so we removed it from the manuscript.

**Reviewer #2:**

The authors show that two modifications to FORCE learning in recurrent neural network (RNNs): (1) Restriction to a lower-dimensional linear subspace, and (2) Tracking this subspace using Oja's rule (unsupervised, compare to Pehlevan/Qin/Chklovsii principal subspace whitening), lead to a learning solution with favourable performance and some ability to automatically track drift. The paper contains new insights that represent a substantive contribution to the field and that will be of broader interest.

That said, I struggled to parse out the core results from this manuscript. I'm therefore requesting considerable major revisions before this moves towards a final published version — but these are not meant in any way to be a criticism of the underlying science. My hope is that they will improve the accessibility and impact of this paper.

Overall, I think the author's have a very nice test-bed for new work. However, manuscript clarity needs to be improved, mathematical insights need to be tightened up, and I am not sure that all claims are substantiated with the analyses as presented (and other claims are phrased in a way that make them sound similar to prior work). I suspect the underlying ideas are good: although the revisions needed are substantial in volume, I expect they are all quite feasible.

We sincerely thank the reviewer for taking the time to review our work. In the past 25 years, not often have I received such detailed comments on a manuscript. Please find below a point-by-point response to your queries.

\## Major:

\### Results

The lack of mathematics in the results section makes this paper difficult to understand. The equations (covered in methods) are the central result of the paper, and it is difficult to interpret the results, figures, discussion without internalising them. I would argue that the whole of the methods should be folded into the results section to support the reader, and I believe there are some examples of PLoS papers that take this approach? The reader needs understand the network structure, dynamics, and which pieces are drifting, perturbed, and plastic (with which learning rules), to set the stage upon which the results will play out.

Ok, we moved large portions of the Methods section into the Results.

\### 140-141: "Standard Force update ill conditioned":

It's important to state what this update actually is (mathematically) so that the reader has a clear understanding of what "ill conditioned" means specifically and why this is the case. Presumably it's something about manifold misalignment limiting the variation that's accessible to learning?

We added the following (lines 249+):

“Intuitively, FORCE assumes that neural activity spans a sufficiently rich high-dimensional space such that small changes in decoder weights can reconstruct the target signal. However, when activity is restricted to a low-dimensional manifold, many directions in neuron space become unavailable, causing the covariance structure of neural activity to become low-rank. In this regime, FORCE becomes ill-conditioned because the decoder attempts to invert activity patterns that no longer contain independent dimensions. This instability arises because FORCE distributes decoder updates across directions that are poorly aligned with the intrinsic manifold. In contrast, SPLiT continuously aligns decoder updates with the dominant low-dimensional activity subspace (Fig 2D). Although FORCE could in principle be modified through a pseudoinverse formulation, or by rewriting the learning rule in intrinsic manifold coordinates, such approaches assume prior access to the manifold structure itself. In contrast, SPLiT provides a synaptic mechanism that continuously estimates and tracks the evolving activity subspace during learning, thereby constraining decoder adaptation to directions supported by the intrinsic population dynamics.”

We can also include more math (in supplementary, section titled “Comparison to FORCE learning”):

FORCE learning updates decoder weights using an online recursive least-squares estimate of the inverse activity covariance. More specifically, FORCE minimizes

$$\mathcal{L}\left( D \right)=\sum_{t} \left\| Dr\left( t \right)-z\left( t \right) \right\|^{2},$$

(S1.44)

where the least-squares solution depends on the inverse covariance matrix

$$C^{-1}=\left( \sum_{t} r\left( t \right)r\left( t \right)^{T} \right)^{-1}.$$

(S1.45)

When activity is constrained to a $d$-dimensional manifold, recurrent activity can be written as

$$r\left( t \right)=Qx\left( t \right),$$

(S1.46)

where $Q\in\mathbb{R}^{N\times d}$, $Q^{T}Q=I_{d}$, and $d\ll N$. The full activity covariance is therefore

$$C=\sum_{t} r\left( t \right)r\left( t \right)^{T}=Q\left( \sum_{t} x\left( t \right)x\left( t \right)^{T} \right)Q^{T},$$

(S1.47)

and hence $\mathrm{rank}\left( C \right)\leq d$. Because $d<N$, $C$ is rank-deficient and cannot be inverted in the full $N$-dimensional neural space. Consequently, the recursive inverse-covariance matrix used by FORCE is ill-conditioned under constrained dynamics. Most neural-space directions have zero variance, and decoder updates along those directions are not identifiable from the data. This explains why FORCE performs poorly when applied directly to full neural activity under low-dimensional constrained dynamics.

By contrast, SPLiT performs learning in the intrinsic coordinates $x\left( t \right)=Q^{T}r(t)$. The corresponding covariance is

$$C_{x}=\sum_{t} x\left( t \right)x\left( t \right)^{T}\in\mathbb{R}^{d\times d},$$

(S1.48)

which can be full-rank if the activity explores the $d$-dimensional manifold. SPLiT therefore avoids inverting a singular $N\times N$ covariance matrix and instead adjusts the decoder in the well-conditioned intrinsic subspace.

The premise that FORCE learning cannot handle low-rank structure without regularisation (or using pseudoinverse to restrict it to appropriate subspaces), appears to be reaching a bit. I think this can be done more thoughtfully, to avoid triggering some who've worked in these areas who might find such modifications obvious/immediate. The authors recover new results about learning speed, weight update magnitudes, etc, and it may be that emphasising the biological interpretability of this is a stronger pitch?

We thank the reviewer for this comment. We agree that our original wording may have overstated the limitation of FORCE learning in low-rank settings. Our intent here is to show that standard FORCE becomes poorly conditioned when recurrent activity is strongly constrained to a low-dimensional manifold. We revised the Discussion as follows (lines 657+):

In contrast, traditional LMS approaches such as FORCE learning exhibit degraded performance in the constrained regime, despite performing adequately when activity spans the full high-dimensional space. This degradation arises from a fundamental limitation of these approaches: when activity is confined to a $d$-dimensional manifold (where $d\ll N$), the covariance matrix of recurrent activity becomes low-rank, with many eigenvalues approaching zero. As a result, decoder updates that depend on inverting the full covariance matrix become numerically unstable and sensitive to noise along weakly represented directions. SPLiT avoids this issue by operating within the intrinsic activity subspace.

Following the reviewer’s suggestion, we also strengthened the discussion of the biological and computational implications of manifold-constrained learning. See Discussion (lines 709+):

Beyond improving task performance, SPLiT learning provides a biologically interpretable account of why learning constrained to intrinsic neural manifolds may proceed more rapidly than unconstrained adaptation. When manifold-constrained dynamics produce low-dimensional population activity, downstream readouts can achieve accurate decoding with relatively small synaptic modifications (Fig 5D). By contrast, unconstrained dynamics require larger and more variable weight updates to compensate for continuously evolving high-dimensional activity patterns. This distinction provides a mechanistic interpretation for experimental observations showing that within-manifold learning in motor cortex occurs on faster timescales than learning that requires activity outside of the intrinsic manifold [22]. More generally, the model suggests that low-dimensional neural population structure may reduce the synaptic burden associated with behavioral adaptation by restricting plasticity to a smaller set of behaviorally relevant degrees of freedom.

We also added this to the Discussion (lines 810+):

Variants of FORCE learning that account for low-dimensional manifolds could likely mitigate representational drift by incorporating regularization or low-dimensional projections. However, our current goal was to provide a biologically interpretable online plasticity mechanism that performs manifold tracking and supervised learning within a cohesive framework.

\### 145:

This paragraph starts talking about constrained/unconstrained RNNs. The methods section defining the RNN and the weight construction for each scenario should be folded in, prior to this paragraph, so that the reader can visualise what these terms mean with appropriate mathematical clarity.

Ok we moved a large portion of the math to the Results section.

\### 146-147: "RNN activity exhibited a drift in manifold":

This is potentially confusing as other senses of drift are used elsewhere in the paper. If I read this correctly, what's happening here probably that the rank N RNN has excess capacity and thus permits multiple solutions (many ways to recover the useful input-driven states from the reservoir).

I can see that "drift" here is defined in Eq. 8. It's not clear to me whether the increased variation just reflects extra (untrained) chaotic activity which is stable (but read as variable by your estimator), or whether there is typically a single low-D manifold that actually changes over time. I expect the former, but if it is the latter it might imply a very strong influence of the feedback connections on this readout?

We thank the reviewer for this important clarification. We agree that our original wording could be interpreted as implying a coherent rotation of a single low-dimensional manifold over time. In the unconstrained simulations, however, the principal-angle metric primarily captures deviations from the initial dominant activity subspace, which can arise from the recruitment of additional high-dimensional activity modes rather than a rigidly rotating manifold.

We have therefore revised the manuscript to clarify this distinction (lines 263+):

Results obtained from a constrained RNN were compared to an unconstrained scenario, where recurrent dynamics were free to explore the full N-dimensional activity space. In this regime, activity no longer remained confined to a stable low-dimensional subspace, yielding large principal-angle deviations relative to the initial activity subspace (Fig 2E-F). These deviations reflect the emergence of additional high-dimensional activity modes rather than a single rotating manifold. When network dynamics were unconstrained, both SPLiT learning and FORCE accurately tracked the sine wave (Fig 2G). In SPLiT, removing the feedback term (I_fb) (Eq. 1) disrupted performance under constrained dynamics but not when dynamics were left unconstrained (Fig 2C vs. 2G).

Also added this sentence (lines 899+):

In unconstrained networks, increases in principal angle should be interpreted as deviations from the initial dominant activity subspace rather than evidence for a rotating manifold.

Further, we replaced “manifold drift” with “changes in activity subspace” wherever relevant.

\### 168: "Two types of perturbations..."

This is another area where inline equations are needed to follow these results; It needs to be made clearer that these perturbations don't change the RNN's input weights, or recurrent weight subspace, but only affect a stage of readout weights omitted from Figure 1b.

It may be useful to mention the extent to which these perturbations change the linear subspace of $r$ via feedback connections (if at all), as a large effect here paints a more complicated picture. My read is that in the constrained networks changing the readout change doesn't change the linear subspace of $r$ very much.

We thank the reviewer for identifying an ambiguity in our description of manifold perturbations. We agree that the original text might incorrectly suggest that perturbations directly modified the recurrent connectivity matrix or the intrinsic recurrent activity subspace itself. We have revised the manuscript to clarify that perturbations were applied at the decoder stage while leaving the recurrent weights $W$ and the intrinsic manifold basis unchanged (lines 286+):

we introduced an abrupt perturbation at the decoding stage while leaving both the recurrent connectivity matrix $W$ and the intrinsic manifold basis $U$ unchanged (Fig 1B).

We also added inline equations defining how the perturbation was applied (lines 289+):

Two types of perturbations were examined. In a within-manifold perturbation, latent coordinates were remapped through an orthogonal rotation within the intrinsic activity subspace,

$$y_{\mathrm{rotated}}\left( t \right)=Py\left( t \right), P^{T}P=I,$$

(15)

where $P\in\mathbb{R}^{d\times d}$ is an orthonormal random rotation matrix obtained by QR decomposition. This perturbation preserves the underlying manifold while rotating the coordinate representation within that subspace. In an outside-manifold perturbation, the decoder basis itself was rotated toward directions orthogonal to the intrinsic manifold,

$$U'=\mathrm{orth}\left( \cos\left( \theta\right)U+\sin\left( \theta\right)U_{\perp} \right),$$

(16)

where $U_{\perp}$ satisfies $QU_{\perp}=0$ and $\theta$ controls the degree of outside-manifold rotation. Both within- and between-manifold perturbations involved rotational changes in manifold structure rather than additive translations or shifts in neural activity. Trials lasted 10 s, with perturbations introduced half-way through each trial. Following perturbations, decoder learning was suspended for 1.6 s while recurrent activity and feedback dynamics continued to evolve. Decoder adaptation was then reactivated and performance was evaluated during the subsequent period.

Also added this (lines 328+):

In constrained networks, perturbations induced only minor changes in the dominant recurrent activity subspace because the recurrent dynamics remained restricted to the fixed low-dimensional manifold ($U$). Thus, performance degradation primarily reflected a mismatch between decoder and recurrent activity rather than a large-scale reorganization of recurrent population dynamics.

Finally, we revised fig.1B and corresponding figure legend.

\### 173: "Marked performance degradation:"

At this point it's not clear whether this is before or after the decoding weights have had time to re-train. I think these results must be post re-training, because otherwise the in-manifold case would not have had a chance to recover performance? In which case, I think the reason out-of-manifold cannot compensate is that the subspace tracking on U is persistently biased by being mixed with a fixed, orthogonal matrix which lies mostly in the null space of $r$.

We thank the reviewer for pointing out this ambiguity. We have revised the text to clarify that the reported performance reflects the post-perturbation period after the decoder was allowed to adapt, except during a short freeze window immediately following the perturbation. This freeze window was included to isolate the immediate effect of the imposed misalignment before adaptive compensation began. The following was added (lines 317+):

With outside-manifold perturbations, error increased with the rotation angle $\theta$ because the decoder basis was rotated toward directions orthogonal to the intrinsic recurrent manifold, thereby reducing the alignment between decoder coordinates and the dominant recurrent activity subspace.

\### 180 "are consistent with"

Is this a bit different from e.g. what one imagines with the BCI results (Schwartz, Feulner), where off-manifold perturbation learning is possible, but quite slow?

A closer analogue to these experiments might be to initialise U to a mostly off-manifold state, and notice how long it takes Oja's rule to re-discover the in-manifold variations and make learning possible?

It also seems to be the case that the perturbations here are *not* modelling gradual drift, but rather an abrupt change --- which is something I missed on the first read as I was biased by other "drift" manuscripts.

We agree that the perturbations studied in Fig. 3 correspond to abrupt decoder remappings rather than gradual representational drift. We have revised the text to make this distinction explicit and to avoid conflating these two phenomena. Modified text (line 283):

To examine the impact of an abrupt remapping of the decoder […]

We also agree that the BCI experiments of Sadtler et al. and related work demonstrate that outside-manifold learning is possible, albeit substantially slower than within-manifold adaptation. Our simulations are consistent with this interpretation: outside-manifold perturbations did not completely abolish recovery but instead produced substantially larger and more persistent decoding error as the decoder became increasingly misaligned with the intrinsic activity subspace.

Finally, we appreciate the reviewer’s suggestion regarding initialization outside the manifold. This is an interesting extension in which the decoder basis initially lies outside the intrinsic recurrent subspace and must gradually realign through manifold tracking. Following the reviewer’s recommendation, we performed additional simulations in which the decoder basis was initialized at varying principal angles relative to the intrinsic recurrent manifold (lines 321+):

As an additional test, we initialized the decoder basis $U$ outside of the intrinsic recurrent subspace. The mean principal angle between $U$ and the intrinsic manifold was initially high but declined rapidly over training (Suppl. Fig S2), indicating that SPLiT continuously drives the decoder basis toward the dominant intrinsic activity subspace generated by the network.

\### 206-215:

It's not immediately clear to me yet what the traced MNIST digits adds, apart from a somewhat harder task. I suppose this shows that loss of even one degree of freedom impacts FORCE learning, but what is the mathematical intuition for the cause of this? This level of sensitivity is a curious result, which probably needs to be examined closer?

The MNIST simulations were intended to test whether the observations obtained with the simple sine-wave task generalized to a substantially more complex temporal trajectory. Importantly, the MNIST task revealed that the failure of FORCE under constrained dynamics was not limited to extremely low-dimensional manifolds but persisted even when the manifold dimensionality approached the full network dimension (e.g., (k=N-1)). This sensitivity shows that the issue is not merely insufficient dimensionality, but rather the presence of rank-deficient or poorly conditioned covariance structure induced by constrained recurrent dynamics.

We added the following (lines 355+):

Because FORCE learning relies on recursive least-squares updates in the full $N$-dimensional neuron space, it becomes ill-conditioned when the covariance matrix is close to singular. Even removing a small number of degrees of freedom can destabilize inversion-based learning updates. By contrast, SPLiT learns within the intrinsic subspace, avoiding the need to invert poorly conditioned directions outside the manifold. The MNIST task makes this effect prominent because accurate digit tracing requires stable decoder updates over many time steps.

\### 217-229 "recurrent gain":

It needs to be clearer in the main text what $g$ actually is, with maths be folded into the main text. It is also a bit ambiguous: $W$ is sampled with random normal weights, with $g$ scaling their standard deviation. But, $W$ is then immediately scaled to have target spectral radius $\rho^*$. As the spectral radius of an $N\timesN$ unit normal random matrix is approximately $\sqrt N$, scaling this by $g/{\sqrt{N}}$ gives an expected spectral radius of $\rho\approx g$. The scaling will then multiply this by $\rho^*/\rho$, removing the impact of $g$ as a parameter.

This makes it hard to interpret this section and Figure 5 --- unless the explicit normalisation of the spectral radius was omitted here? I'm sure I'm missing something, but other readers may have the same confusion? Lines 227-229 might be key here, but I think this needs the usual eigenvalue disk plots for make it clear what's happening?

The reviewer is correct that, in the original formulation, explicitly rescaling the recurrent matrix to a fixed spectral radius would obscure the independent role of the recurrent gain parameter (g). This step was part of an earlier version of the model and was not incorporated in the simulations performed (the newer code has been uploaded along with the revised manuscript). In the revised manuscript, we have therefore removed the spectral normalization step (Eq.4). Consequently, g directly controls the effective recurrent amplification and dynamical regime of the network, consistent with the standard reservoir computing literature and classical results on chaotic recurrent dynamics. We have clarified the text as follows (lines 372+):

In unconstrained networks, increasing $g$ broadens the eigenvalue distribution of the recurrent connectivity matrix and increases the spectral radius of the recurrent dynamics (Fig 5A, top). By contrast, in the constrained regime, recurrent activity is projected to a fixed low-dimensional manifold, suppressing many of the high-dimensional modes that would otherwise emerge as $g$ increases. As a result, constrained dynamics are less sensitive to $g$ (Fig 5A, bottom).

\### 238-250 (un)constrained scaling with λ:

Equation (16) needs to be moved into the main results here to make it clear what λ is. It could be the raw learning rate for un-normalized LMS, it could multiple a unit-length-normalised update $e_i y^\top$, but in this case it's the learning rate for LMS where the variance of the output rate vector $y$ is scaled to $\approx 1$.

Ok we moved Eq.16 (now Eq.12) to the main results (also, we changed from lambda to eta_D to avoid a notation conflict with eigenvalues).

This result may interact with D Raman (2019) "Fundamental bounds..." which analyses how the structure of the Hessian interacts with λ?

We thank the reviewer for pointing out the connection to the theoretical framework developed by Raman et al. (2019), which provides an analysis of how learning depends on network geometry and learning rate. We agree that our findings are related to these ideas. We have added the following (lines 440+):

Recent theoretical work has shown that learning performance depends on the conditioning of the optimization landscape [59]. In particular, Raman et al. [60] showed that learning speed is constrained by the learning rate and the shape of the error surface. Our results are consistent with this framework. By constraining recurrent activity to a low-dimensional manifold, SPLiT reduces the dimensionality of the optimization problem and suppresses high-dimensional activity modes. This produces a more structured feature space, allowing larger decoder learning rates to accelerate convergence without destabilizing learning. In contrast, unconstrained recurrent dynamics generate high-dimensional activity patterns that slow error reduction.

The slope of the improvement also seems to be a bit of a red herring: It's isn't immediately obvious that λ normalised for all changes, and so one might say "well, simply scale λ faster for the unconstrained case". If F5b is correct, however, all simulations show a global minimum around λ=30, and this minimum is lower for the constrained case. Thus, the "faster learning" in the constrained case could be better described as "has access to a markedly better global minimum, and proceeds towards this solution proportionately faster".

We agree that the slope of the MSE versus learning-rate relationship can be misleading because one could compensate for slower learning in the unconstrained regime by increasing the decoder learning rate. We have therefore revised the text to clarify that the key distinction is not merely that constrained dynamics learn faster, but that they converge toward a substantially better global minimum (lines 428+):

A series of simulations examined the impact of the decoder learning rate ($\eta_{D}$). In the constrained case, error decreased rapidly as $\eta_{D}$ increased (Fig 5C), whereas the unconstrained case exhibited a shallower improvement. Power-law fits revealed that error scaled approximately as MSE~$\eta_{D}^{-\beta}$, with a larger exponent for the constrained network ($\beta$=1.498, R^2^=0.955) than the unconstrained network ($\beta$= 0.653, R^2^=0.819). Thus, manifold-constrained learning not only approached optimal solutions more rapidly as $\eta_{D}$ increased, but also attained lower error rates.

These results suggest that constraining recurrent dynamics to a stable low-dimensional manifold improves decoder optimization. In the constrained regime, learning occurs within a compact and well-conditioned feature space, allowing larger learning rates to accelerate convergence without destabilizing the decoder. In contrast, unconstrained dynamics generate high-dimensional activity patterns that produce poorer conditioning and higher residual error.

The result that appears relevant for biology is the performance increase that you can get for a fixed amount of synaptic plasticity. This is interesting, but may be more related to overall higher attainable performance rather than a capacity for faster learning?

We agree with the reviewer that the biologically relevant point is not simply “faster learning”, but rather the relation between learning performance and the amount of synaptic modification required to achieve it. We clarified the text as follows (lines 450+):

Unconstrained dynamics generate high-dimensional and continuously drifting activity patterns, forcing the decoder to make larger corrective adjustments to track the target output. In contrast, constraining activity to a stable low-dimensional manifold produces well-structured features, allowing accurate decoding to be achieved with relatively small weight modifications. This is relevant from a biological perspective given that large synaptic updates are metabolically costly and could have a destabilizing effect on neural dynamics.

There is another possibility, which would render this result trivial and needs to be controlled for: The unconstrained case simply has more useless variation in $y$, which attenuates the magnitude of the relevant in-magnitude learning signals when you normalize $y$ to unit length. A fairer comparison, then, would be to scale $y$ so that the variance of the in-manifold subspace is matched between the constrained, unconstrained conditions?

We agree that this is an important control. We therefore repeated the learning-rate analysis after matching the variance of the parallel component across constrained and unconstrained regimes (lines 456+):

As an additional control, simulations were performed where we equalized the variance of the in-manifold activity between constrained and unconstrained regimes. The performance of constrained networks persisted in this scenario (Suppl. Fig S5). Thus, the performance of constrained networks cannot be explained solely by a lower variance in activity. Rather, the computational advantage stems from improved optimization under constrained dynamics.

\### 282 Learning noisy target signals

This seems to be a key result. Would you get the same outcome if you forced the feedback or output through a low-rank projection, but left the latent dynamics full rank?

We added the following (lines 502+):

To determine the contribution of feedback ($I_{\mathrm{fb}}$) to robustness to noisy targets, simulations were performed where recurrent dynamics remained unconstrained while the feedback was restricted to a low-dimensional subspace. Lowering the rank of the feedback to 1 produced only minimal changes in decoder performance compared to rank $k$ (Suppl. Fig S6). Thus, the improved robustness of manifold-constrained learning was largely independent of the feedback rank. Rather, robustness arises from constraining the recurrent dynamics themselves, which suppresses high-dimensional activity modes.

\### 329 Comparisons to related approaches

I am struggling to get much out of this section, mostly because the structure of the other learning rules is not conveyed in the text. The reader cannot contemplate how their mathematical particulars lead to these differences.

We moved the math description of alternative models to the main text.

There isn't much new intuition from this section, other than to show that there exists a benchmark such that SPLiT is superior. This more limited results might be better conveyed in a compact table, as Figure 8 provides little intuition?

Ok, we edited Figure 8. We find this visually better than a table.

Also, sinsusoid learning is quite simple, perhaps too simple? Could figure space be better used showing how these other networks solve traced-MNIST or the chaotic attractors in F9?

Ok we added chaotic attractors to Figure 8.

\### 337-369

Without a mathematical summary of each rule, it is difficult to take away real intuition from this section. It feels like a dry list.

Could it be composed into some sort of full-page-figure-table insert in some way? A graphic design inspired by metanalysis tables of studies exploring various genes or anatomical connections from physiology? (light suggestion)

As mentioned earlier, we moved the bulk of the math description to the main text.

\## Figures:

\### Figure 1:

Would it make sense to move r(t) into one of the circles, and W a bit closer to the mid of the arrow, to make it faster to gloss that these are states and weights, respectively?

Ok done

The methods appear to show feedback projections from z to r in the simulations, these should probably be included as they are structurally important?

Ok done

I think it would be very helpful to cue the reader as to which parts of the model are fixed, plastic, and drifting somehow. It seems important that the input and recurrent structure is fixed, and that all drift (and perturbations) take place in the mapping from r to z. My hunch is there should be a third node or box between the RNN and z cuing the reader that this is where drift happens. Some annotation that D is plastic may be nice as well?

Ok we expanded figure 1 accordingly.

I'm struggling to interpret (A) literally. As drawn it appears to show that learning is constrained in a way that keeps neuronal states in a specific subspace. If I understand the simulations correctly, the low-d subspace of the RNN is actually fixed. Then, drift and other perturbations are optionally added to the readout, before passing to readout weights that learn via LMS. There is also principle subspace tracking on the output via Oja's rule on an orthonormal matrix U, but both drift and plasticity are downstream of this. Thus, the statistics of the states that drive the evolution of U only change to the extent that changes in fed-back of the read-out "z" alter the correlation structure of r? With this read, "constrained plasticity" might actually be (mostly) "plasticity using an RNN reservoir with fixed, low-rank recurrent weights".

We thank the reviewer for this point. You are correct that, in the manifold-constrained simulations, the recurrent dynamics themselves are restricted to a fixed low-dimensional recurrent subspace through projection of the recurrent connectivity matrix. In this sense, the constrained reservoir is fundamentally low-rank. SPLiT does not itself impose low-dimensional recurrent activity through the learning rule alone. Rather, SPLiT combines (i) low-dimensional recurrent dynamics, (ii) online manifold tracking through Oja’s rule, and (iii) decoder adaptation within the tracked manifold coordinates. Along with other clarifications mentioned earlier, we have revised Fig. 1 to make this distinction clearer.

\### Figure 2:

It may be that there are nice intuitive cartoons that explain why each of the results in C, F happened. (optional)

Done. See Figure 2G.

\### Figure 3:

Ensure that the center-out diagrams are scaled to be circular, the current (squished) format is not to publication quality.

Done.

There are a few things here I'm confused about, in relation to text lines 160--186. Most of these are probably my own confusion, but I think I will not be the only reader that struggles to interpret the results, so there is merit in updating his.

"shifting activity": This could imply an in-manifold translation (and the figure shows a translation as well). However, the methods seem to describe it as a rotation? This matters, I think. If the network has learned to leverage the feedback connections, then a DC bias may be compensated more easily? It may be that with feedback translations can be compensated without updating the readout weights, but for rotations this would only work when the angle is small (so that feedback corrections remain correlated with the true direction)?

The reviewer is correct that the perturbation is fundamentally implemented as a rotation, not a translation. We clarified figure 3 caption:

After training, a within-manifold perturbation was applied by rotating activity within the intrinsic manifold coordinates, which did not disrupt performance.

This has also been made more obvious by the math we added to describe decoder basis rotations, specifically Eq.16.

Further, we replaced the straight arrow in Fig. 3B with a rotational arrow and did the same thing for fig.1A.

In D we see accuracy as a function of perturbation angle. My read is that this is after both the Oja updates on U and the LMS updates on D have had a chance to settle, post learning? Based on Eq 20 definining out of manifold perturbations, small θ should amount to no perturbation, which raises the question of why the constrained MSE remains ~3 for θ=0 out-of-manifold perturbations? Should these not reduce to an in-manifold perturbation (or no perturbation at all)?

The issue is that small residual error remained because performance was evaluated during a finite post-perturbation recovery window while manifold tracking and decoder adaptation were still evolving. We re-ran these two panels using a larger window.

Finally, note that for unconstrained dynamics, the MSE floor above zero reflects baseline unconstrained decoding error (now shown in black dashed lines in Figure 2D).

\### Figure 5:

The task and network parameters are used for these simulations should be documented in the caption.

We amended the first sentence of that section (line 370):

In follow-up simulations using the MNIST task,[…]

\### Figure 9:

Richer figures akin to F78 might be nice here? I think F8 is possibly redundant, visualization might be better served here?

See revised Figure 8.

\## Discussion

\### 411 "regulate the geometry of supervised learning"

I wasn't quite sure what this meant specifically, to me it came across as a vague way to say "an effect happened". Is there a way to be a bit more specific, incisive, clear?

Replaced as follows (lines 682+):

To our knowledge, this is the first model to demonstrate that online manifold tracking can transform supervised learning from a poorly conditioned full-rank regression problem into a learning rule that is restricted to a time-evolving low-dimensional subspace.

\### 428 "reduced sensitivity to recurrent gain"

This is probably a key result, but needs to be supported and conveyed a bit better in the main results. There needs to be some quantification of the extent of recurrence through the internal weights verses the output feedback pathway. Deeper mathematical intuition regarding the recurrent gain result should not be too difficult to add, although I'm still confused about how $g$ and the spectral radius normalization interact.

We added the following (lines 397+):

Under constrained dynamics, recurrent activity evolves within a rank-$d$ subspace. Because decoder learning depends only on activity projected into this low-dimensional subspace, increasing $g$ cannot recruit additional high-dimensional modes. In contrast, unconstrained networks allow gain-dependent amplification across the full recurrent spectrum, producing increasingly high-dimensional activity as $g$ increases.

We also added this (lines 415+):

In the absence of feedback ($\alpha$=0), constrained networks exhibited high error that remained largely insensitive to $g$, indicating that constrained manifolds alone did not lead to accurate decoding (Suppl Fig S4A). Increasing feedback strength ($\alpha$>0) reduced error across all values of $g$. Across values of $\alpha$, decoder performance remained insensitive to recurrent gain, suggesting that manifold-constrained dynamics suppress instabilities due to recurrent gain. The relative drive of feedback and recurrent activity was quantified using the mean ratio

$$R_{\mathrm{fb}}=\frac{1}{T}\sum_{t=1}^{T} \frac{\left\| I_{fb}\left( t \right) \right\|}{\left\| Wr\left( t \right) \right\|}.$$

(18)

Across values of $\alpha$>0 and $g$ between 0.5 and 1.5, $R_{\mathrm{fb}}$ remained above one, indicating that feedback input was larger than recurrent input, and hence played a dominant role in shaping the instantaneous recurrent dynamics during learning.

Also, when you say "noise added to target", won't this also include output noise feedback through $I_\text{fb}$ when running in generative mode (not training). So, this is more generically about how output errors excite the dynamics in $r$. This is probably important to examine, as heavy use of $I_\text{fb}$ can give control-based solutions that are more robust to perturbations, at the expense of allowing noise to excite the dynamics (there is a well known tradeoff in control).

changed to “robustness to perturbations propagated through feedback” (line 701)

We also added (lines 403+):

Because the target signal enters the recurrent dynamics through the feedback term $I_{fb}(t)$ (Eq. 1), noisy targets can perturb the network not only through decoder error but also by injecting fluctuations directly into recurrent activity. To quantify this effect, we measured the mean trajectory deviation

$$\Delta r=\frac{1}{T}\sum_{t=1}^{T} \left\| r_{noisy}\left( t \right)-r_{clean}\left( t \right) \right\|,$$

(17)

while varying feedback strength $\alpha$. Using frozen initial conditions, we compared recurrent activity $r_{noisy}\left( t \right)$ generated when the target signal contained additive noise (zero-mean, unit variance) against activity $r_{clean}\left( t \right)$ generated with a noise-free target. Increasing feedback strength produced progressively larger $\Delta r$, suggesting a trade-off between stronger feedback improving learning while at the same time allowing target noise to disrupt recurrent dynamics (Suppl. Fig S3). Perturbations induced by noisy feedback remained smaller than the variability $\Delta r$ observed across different initial conditions with a noise-free target. Hence, feedback-driven perturbations produced smaller alterations in recurrent activity than the variability induced by differences in initial conditions alone.

\### 431 "SPLiT transforms a poorly conditioned full-rank inverse problem into a well-conditioned projection onto the principal subspace"

I think it may be strategic to hedge here; There is part of the field that will say "this disappears immediately if I put a pseudoinverse or low-rank projection here, and that's the obvious standard engineering choice".

To me, the key novelty was more that Oja or Pehlevan-Chklovsii subspace whitening solutions can be slotted in to handle this low rank learning in a flexible and biologically plausible way.

It's not that SPLiT fixed ill conditioning (this may be generally easy), it's that (presumedly?) the fixed used here is likely to be more similar to the one that the brain uses?

Also, I still feel like "ill conditioning" needs to be spelled out more clearly as this could mean hundreds of specific things.

We revised the text as follows (lines 703+):

Constraining activity to a low-dimensional subspace reduces the effective dimensionality of the decoder regression problem by restricting learning to the dominant covariance modes of population activity. While similar improvements in conditioning could be achieved using low-rank projections or pseudoinverse regularization, the novelty of SPLiT lies in providing a biologically motivated online mechanism for continuously estimating and tracking this subspace during learning.

And this to clarify ill conditioning (lines 660+):

This degradation arises from a fundamental limitation of these approaches: when activity is confined to a $d$-dimensional manifold (where $d\ll N$), the covariance matrix of recurrent activity becomes low-rank, with many eigenvalues approaching zero. As a result, decoder updates that depend on inverting the full covariance matrix become numerically unstable and sensitive to noise along weakly represented directions. SPLiT avoids this issue by operating within the intrinsic activity subspace.

\### 438 "while slower learning reflects the need to reshape those degrees of freedom themselves"

A major deficiency of this paper appears to be the lack of any real tracking of drifting manifolds by the dynamics on $U$. Since the input and recurrent weights are fixed, the subspace of $r$ is also fixed, and $U$ is hard coded to the dimension of this subspace. Thus, $U$ need only converge during the initial learning, and no further tracking (e.g. during perturbations) is needed. Indeed, the way perturbations are done they actually hard-code a countermand to $U$'s learning that plasticity in $U$ cannot compensate (hence the large steady-state errors even after re-training?)

If I read the math correctly, what this paper is missing is a slow, drift-like change in how the low-$d$ variations in $r$ are presented to the readout $U$, which could highlight the learning benefits of Oja's rule for tracking evolving subspaces. Line 438 seems to almost imply that this analysis was happened, and is phrased as if it is a conclusion of the analysis in the paper. At present, however, I suspect it is more of a distillation of prior drift results that comes to mind when contemplating this work?

We thank the reviewer for identifying an important limitation of the original constrained-manifold formulation. We agree that when the manifold basis is fixed, the activity subspace is effectively stationary, and the role of online manifold tracking is therefore reduced after initial convergence. To address this concern, we added a new rotational drift model in which the manifold basis evolves continuously according to a small incremental rotation matrix. Under this formulation, the covariance structure of recurrent activity changes continuously over time, requiring ongoing adaptation of the estimated principal subspace U(t). See the new subsection “Online subspace tracking stabilizes learning under rotational drift” (lines 623+):

Simulations were performed with a gradual rotational drift (Suppl. Fig S8A). The manifold basis evolves as

$$Q\left( t+\Delta t \right)=R\left( \Delta\theta_{t} \right)Q(t)$$

(34)

where $Q\left( t \right)\in\mathbb{R}^{N\times d}$ is the orthonormal basis spanning the low-dimensional manifold at time $t$, $\Delta t$ is the simulation time step, $R\left( \Delta\theta_{t} \right)\in\mathbb{R}^{d\times d}$ is a rotation matrix, and $\Delta\theta_{t}$ is the incremental rotation angle. Under rotational drift, decoder performance depended on the strength of feedback (Suppl. Fig S8B,C). This suggests that rotational drift relies on stabilizing feedback, allowing the decoder to remain aligned with the evolving population subspace. FORCE learning (Suppl. Fig S8B) and alternative models did not perform accurately on this task (Suppl Fig S9).

Next, we extended these results to the center-out reaching task with rotational perturbations. For most models, within-manifold perturbations yielded worst performance than between-manifold perturbations (Suppl. Fig S10). This result differs from SPLiT, where performance was more sensitive to between- than within-manifold perturbations (Fig 3D), suggesting that tracking of the intrinsic low-dimensional activity subspace is critical for preserving decoder alignment under manifold drift.

To further establish whether online subspace tracking was required under rotational drift, simulations were performed where the manifold basis $U\left( t \right)$ remained frozen once initialized. In these simulations, the frozen basis remained poorly aligned with the evolving population subspace (Suppl. Fig S11A). By contrast, allowing $U\left( t \right)$ to adapt over training improved its alignment with the evolving manifold. This improved alignment was accompanied by a lower decoding error (Fig S11B,C). Thus, under manifold drift, online subspace tracking contributes to maintaining decoder alignment and improving task performance.

Finally, simulations were performed to examine whether SPLiT could recover from a rotational perturbation introduced after training on the sine wave task. Small rotations produced little disruption to performance, whereas larger rotations caused progressively larger increases in error (Suppl. Fig S12). Even for large rotations, trhiSPLiT maintained lower error than simulations where the manifold basis $U\left( t \right)$ remained frozen, indicating that online subspace tracking improves decoding performance across a range of perturbation strengths.

Further, we have revised the Discussion to clarify that the original perturbation experiments examined imposed decoder misalignment, whereas the new rotational drift model explicitly examines continuous manifold evolution and online subspace tracking (lines 669+):

A first series of simulations involved perturbations that imposed a misalignment relative to a fixed intrinsic manifold (Fig 3A-C). In those simulations, the low-dimensional activity subspace remained stationary after initialization, such that subspace tracking by SPLiT served to align decoder weights with a fixed structure. To more directly model representational drift, we subsequently introduced rotational perturbations where the manifold basis evolved continuously over time (Suppl Fig S8A). Under these conditions, online adaptation of the estimated subspace became necessary to maintain alignment between decoder weights and the evolving population manifold. Simulations with a frozen manifold basis $U\left( t \right)$ showed that manifold tracking improved both manifold alignment and decoding accuracy under drift, demonstrating that SPLiT performs online manifold tracking and not simply learning within a static low-dimensional subspace.

We also added new results in Suppl. Fig. S11 (line 636+):

To further establish whether online subspace tracking was required under rotational drift, simulations were performed where the manifold basis $U\left( t \right)$ remained frozen once initialized. In these simulations, the frozen basis remained poorly aligned with the evolving population subspace (Suppl. Fig S11A). By contrast, allowing $U\left( t \right)$ to adapt over training improved the alignment of $U\left( t \right)$ with the evolving manifold. This improved alignment was accompanied by a lower decoding error (Fig S11B,C). Thus, under manifold drift, online subspace tracking contributes to maintaining decoder alignment and improving task performance.

\### 443 "was able to perform successful learning"

"could learn", perhaps?

fixed.

\### 445 "related work [60]"

Since there is only one paper here, charitable norms would be to cite the authors by name; "Feulner and Clopath (2021) show...".

Fixed.

Rather than saying "their framework did not", a more charitable take could read "SPLiT extends this work by positing an explicit, biologically-plausible plasticity mechanism to adapts readout weights in tandem with evolving population subspaces, maintaining subspace alignments required for rapid learning".

Fixed.

Consider also if similar notes apply to how (2,3,4) are quoted and discussed on lines 451 457 462?

Ok we applied edits as appropriate.

\### 451 "combining Hebbian and homeostatic"

I believe [31] as cited also uses Oja's rule to track drift, and further includes a study of recurrent dynamics to projec-out off-manifold variability. These works are different from Godin and Thivierge, but contain more similar structural elements than what is implied by this phrasing.

Ok we replaced with the following (lines 570+):

Second, we compared SPLiT to related approaches combining Hebbian and homeostatic plasticity to stabilize drifting neural representations (O-LMS) [31,40]. Rule and O’Leary proposed that Hebbian plasticity, homeostatic regulation, and recurrent feedback could allow downstream readouts to continuously track evolving population codes without requiring explicit external error signals. Their framework demonstrated that recurrent dynamics suppress off-manifold variability by reinforcing stable population-level correlations. SPLiT shares several conceptual elements with this approach, including an online adaptation to representational drift, a low-dimensional population structure, and the use of biologically motivated plasticity rules. However, a key difference is that SPLiT integrates supervised learning with online manifold tracking. In O-LMS, the supervised error follows Eq. 20 and the decoder evolves according to

$$\dot{D}\left( t \right)=\eta_{\mathrm{LMS}}e\left( t \right)r^{T}\left( t \right),$$

(25)

with learning rate $\eta_{\mathrm{LMS}}$=0.01. This rule depends solely on instantaneous presynaptic activity and scalar error. Because this rule is not formulated to capture temporal dependencies of the target signal, it performed poorly under constrained dynamics (Fig 8A,B).

\### 453 "the form of drift considered here operates at the population level and manifests as changes in low-dimensional neural manifold"

As noted by Qin and colleagues, drift in vivo (and as modelled in 40 and elsewhere), largely consists of incremental rotation of the low-$d$ manifold encoding population activity (whilst preserving encoded structure. This is implied in how Driscoll (2017) was conveyed and interpreted in subsequent early works on drift. The early drift work ecompasses the type of drift that this claim appears to tag as new in Godin and Thivierg?

Revised as follows (lines 773+):

*(2) Online LMS.* Another relevant line of work proposed a synaptic mechanism for tracking representational drift by combining Hebbian and homeostatic plasticity [31,40]. In these studies, drift emerges from ongoing synaptic turnover and gradual reorganization of neural representations. Subsequent experimental and theoretical work has shown that such drift manifests as incremental changes in low-dimensional population manifolds while preserving task-relevant structure [22,36]. SPLiT builds on this view of representational drift. However, unlike previous approaches, SPLiT combines online subspace tracking with a supervised decoder.

It is also a bit confusing to class the specific manipulations in "Godin and Thivierg" as "changes in low-dimensional neural manifold", if I've parsed the math correctly. It looks like the only way the manifold for $r$ can really change is via signals arriving as $I_\text{fb}$, which (as noted in Methods), can at best change how off-manifold transients in $r$ are excited, or somewhat adjust the covariance structure of $r$ within its constrained rank $d$ subspace. Gradual drift of population codes does not appear to be explored (or at least was not clearly conveyed in the current writeup)?

The new rotational drift simulations address this concern. Also, we added this sentence to the first paragraph of Discussion (line 654):

This mechanism allows the decoder to align with low-dimensional population structure during learning.

\### 455 It is not clear how the combination of Hebbian and homeostatic plasticity could be extended to successfully track collective subspace dynamics.

The setup in Godin and Thivierg may have the potential to explore these dynamics, if the perturbations took the form of gradual rotations that mixed in- and off- manifold spaces, and if these were placed *before* the readout weights $U$ that follow Oja's rule for subspace tracking. In this case, there are three scenarios to explore. Firstly, failure of $U$ to adapt to changes will remove the correlated variability needed to update decoding weights $D$, degrading task performance. There is a (second) null result that successful tracking in $U$ simply retains the capacity to learn. This is quite like Qin's result, in that the ongoing LMS error feedback would still be needed to re-learn the task to compensate for in-manifold rotations. Thirdly, there is also some possibility that running Oja's rule on $U$ performs internal transfer learning more like [31], and somehow leads to savings that cause re-learning to be faster than what could be explained by plasticity in $D$ alone. These are the sort of analyses I'd expect to have seen with this model, to substantiate line 455? Then again, it may be that it was exactly these that were performed. In which case, this can be resolved by presenting results more clearly?

The new rotational drift simulations, and simulations on frozen vs. adaptive U partly address these concerns. Further, we added the following (lines 643+):

Finally, simulations were performed to examine whether SPLiT could recover from a rotational perturbation introduced after training on the sine wave task. Small rotations produced little disruption to performance, whereas larger rotations caused progressively larger increases in error (Suppl. Fig S12). Even for large rotations, however, SPLiT maintained lower error than simulations where the manifold basis $U\left( t \right)$ remained frozen, indicating that online subspace tracking improves decoding performance across a range of perturbation strengths.

\### line 457 on Predictive coding. I'm not sure there's a strong distinction between predictive coding and supervised learning here. In predictive coding, it's generally possible to clamp various input/output/edge nodes to varying degrees, which controls the influence of external (supervised) inputs on internal (generated) "predictions" that sets the activity statistics for learning. These properties are dual, and for example a quantity that is a descending prediction becomes an external input if the network order is reversed. And, more general topologies can mix more than two inputs and outputs, rendering the distinction less clear. In other words, I would not call predictive coding unsupervised.

Edited as follows (lines 780+):

(3) Predictive coding. Learning rules based on predictive coding assume that circuits continuously minimize prediction errors arising from mismatches between internal latent states and external inputs [61–63]. Depending on the architecture, predictive coding frameworks can incorporate supervised learning signals. The key difference relative to SPLiT lies in how representational drift is handled. Predictive coding models generally optimize latent-state inferences and prediction consistency, whereas SPLiT explicitly performs online tracking of evolving low-dimensional population subspaces through Oja-based manifold estimation coupled to supervised decoder adaptation.

\### 464

It is probably appropriate to cite Qin et al (2023) "coordinated..." here, and likely elsewhere.

ok we added (line 789): or an exploration of degenerate representational manifolds during Hebbian learning [70].

We also added this on the Qin paper (lines 733+):

Consistent with this view, Qin et al. [70] proposed that drift can emerge from noisy Hebbian learning in networks with multiple equivalent representational solutions, producing coordinated manifold rotations while preserving population-level similarity structure.

\### 477 "learning speed should decrease"

I don't think this is a specific enough prediction. Andy Schwartz's group have already shown that in vs out-of-manifold readouts lead to different learning rates. And, by the intermediate value theorem, smoothly interpolating between these will also smoothly interpolate learning rates. With appropriate normalisation, it's would be trivial to interpret this as cosine similarity, and sweep minor differences under the rug of "slight deviations reflect divergences between the brain's internal measures of representational similarity and the metrics used here". This is also a prediction largely implied by Feulner and Clopath, and so is not new to Godin and Thivierg. Is there a way to add a bit more Godin-and-Thivierg-specific meat to this?

Modified as follows (lines 823+):

First, SPLiT predicts that recovery from outside-manifold perturbations depends not only on changing decoder weights, but also on continually tracking the evolving structure of population activity. In our simulations, freezing the manifold estimate $U(t)$ reduced performance (Suppl. Fig S12), even when the decoder was still allowed to adapt. Experimentally, this idea could be tested by allowing the BCI decoder to remain plastic while holding fixed the neural subspace used by the decoder. SPLiT predicts that recovery should be impaired when decoder plasticity is preserved but online updating of the neural manifold is prevented.

\### 484 "smaller decoder updates"

This is not actually a prediction about biology. It appears to be saying that... if a BCI decoder were to use the same structure as SPLiT, then it would exhibit the same learning properties as characterized in this paper? But, the decoder is a design choice, and not a fact about biology. So, this appears to reduce to a claim more like "we conjecture that the results here for SPLiT are generic and will reproduce across a wider range of learning problems than the ones explored here". This is all well and good, but can something deeper be said?

Changed to (lines 830+):

Second, SPLiT assumes a separation of timescales between the relatively slow evolution of the low-dimensional manifold and the faster updates of the manifold-tracking rule. A prediction derived from this assumption is that improving the speed or accuracy of manifold tracking should accelerate recovery from outside-manifold perturbations. Experimentally, this prediction could be tested in a closed-loop BCI system by comparing decoders that update estimates of the neural activity manifold at different timescales. SPLiT predicts that, up to a point, faster manifold tracking should improve decoder-manifold alignment, accelerate recovery from outside-manifold perturbations, and reduce sensitivity to noise in the target signal.

\### 494

I wonder if this idea is related to David Brandmann's decoder that found some benefit to adding an adaptive, Gaussian-process feature learning stage prior to the adaptive linear readout, in BCIs?

We added (lines 838+):

This prediction is related to prior adaptive BCI approaches that incorporate online feature learning prior to readout adaptation, such as the GP-DKF framework of Brandman et al. [84]. However, SPLiT makes a more specific prediction: adaptation speed should depend not merely on adaptive feature learning per se, but on the ability of the decoder to maintain alignment with the evolving low-dimensional covariance structure of neural population activity. In SPLiT, recovery from outside-manifold perturbations depends on online tracking of the intrinsic activity subspace itself, rather than solely on nonlinear decoding.

I think this paragraph may be THE KEY result of this work, and is burying the lede. Suppl. Info. Eq. S1.7 and some of these supplemental analyses likely already contain the results needed to address above-noted confusions in the main text, and pivoting to emphasise them might be a good idea?

We thank the reviewer for highlighting this point. We agree that the dependence of SPLiT on the relationship between manifold drift and online manifold tracking constitutes a central conceptual result of the work. We revised the manuscript (lines 830+ as mentioned earlier, and lines 838+).

\### 504 An intriguing possibility is that SPLiT could allow a receiving area to track the manifold of a sender population and adapt its readout accordingly, thereby preserving information transfer despite ongoing drift.

As phrased, this appears identical to something already solved in results presented in [31] (unsupervised form) and Qin et al (2023) (supervised form)? This does not appear to be a new prediction or capacity of SPLiT. But, SPLiT is structurally different from these works. I believe it has specific innovations that should be highlighted here?

Revised as follows (lines 850+):

An intriguing possibility is that SPLiT could allow a receiving area to continuously estimate and track the low-dimensional covariance structure of a sender population while restricting supervised adaptation to the instantaneous intrinsic subspace. While related models have proposed adaptive readouts capable of compensating for representational drift [31,70], SPLiT differs in that manifold tracking is treated as a key component of learning that is distinct from the decoder. In this framework, successful adaptation depends not only on decoder adaptation but on the alignment between decoder coordinates and the evolving activity manifold through online estimation. This predicts that inter-area communication under drift should depend on the trackability of low-dimensional population structure rather than solely on readout plasticity.

\## Minor:

On a first read, I made a note that that the abstract, summary might be tightened up with a few more editing passes. The ¶ Lines 102-111 struck me as not quite carrying their weight, with the claims at this point phrased in a way that doesn't clearly distinguish them from the picture of representational drift in prior work. I would invite the authors to revise these after folding in all reviewer comments.

Revised as follow (lines 105+):

We demonstrate that SPLiT supports learning in networks with dynamically evolving manifolds by coupling online manifold tracking to supervised decoder adaptation. Unlike approaches that compensate for drift through readout plasticity alone [26,31,40,55], SPLiT continuously estimates the empirical low-dimensional activity subspace and restricts learning to manifold coordinates. This allows decoder weights to remain aligned with evolving population dynamics while suppressing unstable high-dimensional activity modes. Using a rate-based recurrent network, we show that SPLiT reliably learns time-varying target signals under both constrained and unconstrained dynamics, yielding faster convergence, smaller weight updates, and greater robustness to noisy teaching signals than full-rank learning rules. Analytical results demonstrate that SPLiT learns the optimal decoder projected onto the instantaneous principal subspace and maintains bounded error under drift. These results show that online tracking of low-dimensional population activity constitutes a computational mechanism for stable supervised learning under representational drift.

I would invite the authors to do an editing pass for wordiness, replacing (if desired) "novel"-> "new", "in order to" -> "to", "is able to successfully"->"can", and similar.

Ok we did an editing pass to tighten the text.

If cross-referencing "Methods", is it possible to link to the relevant part of methods to be more specific?

We move the bulk of Methods to the Results section and ensured that other parts are cross-referenced appropriately.

Watch out for equivocation: "marked" "substantive" "robust" "readily"; In some cases text may read stronger without qualifiers.

Ok we removed unnecessary qualifiers.

**Reviewer #3:**

In this study, the authors provide a computational account of how stable learning may take place even in the face of representational drift. Through their proposed plasticity rule called SPLiT (Synaptic Projection Learning with intrinsic Tracking), they demonstrate how it may be beneficial to simultaneously track the evolving neural manifold during supervised learning, to allow for faster learning and robustness to noise. The SPLiT learning rule has two components: (1) an unsupervised Oja rule to track the manifold in an online manner and (2) a Least Mean Square (LMS) algorithm to train the readout weights of the recurrent neural network (RNN) based on the manifold coordinates.

The paper is well structured, and I thank the authors for their clarity of writing. I really liked how the paper aligns with the findings from the seminal BCI study by Sadtler et al. (2014), which highlighted important differences between learning within and outside manifold. This empirical connection, together with their results demonstrating the possible advantages of manifold-constrained learning using the SPLiT rule, are in my view the study’s strongest contributions.

As the authors themselves emphasize, the paper’s aim is not to provide a biologically plausible account of how the manifold may be tracked or how the readout weights may be trained, but instead to understand at a computational level how learning may adapt to representational drift. I understand the authors’ viewpoint, but I do believe that this paper would benefit from a more thorough treatment of the following points:

Major comments:

- Although SPLiT may be an abstraction and it is acknowledged that an explicit projection may not actually take place (and only allows here for a controlled experimental setting), it is not clear what possible underlying biology would be consistent with the current framework. Here, the projected activity y(t) does not map onto any set of neurons and lives abstractly somewhere between the activity r(t) and the network output z(t). Are the authors suggesting that this low-dimensional structure would live in the activity r(t) itself? If so, would it need to be tracked by a subset of the neurons, or estimated by another network? The Oja rule itself is considered biologically plausible (ignoring the normalization), but I am not entirely sure how it would be implemented here. How would the decoder/readout gain explicit access to the low-dimensional structure? Given how central this point is to the paper’s main arguments, I would be grateful if the authors could shed further light on this, either through toy experiments or via a deeper conceptual exposition.

Thank you for this insightful comment. We clarified the text as follows (lines 183+):

The manifold coordinates $y\left( t \right)$ should not be interpreted as requiring a distinct neural population that represents a latent state separate from the recurrent activity $r(t)$. Rather, $y\left( t \right)$ corresponds to dominant covariance patterns embedded within the population activity. Thus, the projection in Eq. 8 provides a description of ongoing activity rather than a separate dynamical variable.

Also, in response to another reviewer’s comment, we now include simulations where we initialized the decoder basis outside the intrinsic recurrent subspace (Suppl. Fig.S2). Results show that prior knowledge of the manifold is not required, as the decoder can recover the dominant covariance structure online.

- The abstract, introduction, discussion and methods sections are well structured and easy to read, but I wonder whether the results section could be finessed through more informative sub-headings that convey the main message of the relevant section, e.g., “manifold-constrained learning confers robustness to noise” instead of “learning noise target signals”. The flow is not entirely clear from the sub-headings, and I feel that the results section could be tightened in general.

Ok we clarified the subheadings

- The comparison between SPLiT and FORCE in the constrained case seems somewhat contrived and not fully justified to me. FORCE will fail by design given that it is implicitly inverting the full covariance matrix. Is the comparison to FORCE really necessary here, or could a comparison be made for instance using a learning algorithm that does not involve the full covariance matrix?

Added to Discussion (lines 794+):

*(5) FORCE learning*. While FORCE learning is a well-established supervised learning algorithm for training recurrent neural networks to generate complex trajectories [53], it performed poorly when recurrent dynamics are constrained to a fixed low-dimensional manifold (Fig 2C). This failure happens because FORCE relies on recursive least-squares updates in the full population space and therefore assumes sufficiently rich, full-rank activity covariance. When activity is confined to a low-dimensional manifold, many directions in neuron space become unavailable, rendering covariance inversion ill-conditioned. Thus, the constrained regime considered here violates a core assumption underlying FORCE learning. We nevertheless included this comparison because it illustrates a broader point central to the present work: learning rules that operate directly in the full activity space can become unstable when neural population activity is intrinsically low-dimensional.

Also added (lines 804+):

Importantly, impairments under constrained dynamics were also observed for several alternative plasticity rules that do not explicitly rely on full covariance inversion (Fig 8), including O-LMS, predictive coding, associative memory, Bayesian inference, and continuous-learning approaches. The comparison to FORCE should therefore not be interpreted primarily as a criticism of recursive least-squares methods themselves, but rather as an illustration that learning rules operating in the full neural activity space can degrade when population activity is low-dimensional.

- More generally, I am not entirely sure how to view comparisons with related approaches in the constrained scenario (where other approaches underperform since they do not estimate the low-dimensional structure). I think this probably links back to my first comment (and can be answered together), but is this comparison well-defined biologically? The constrained regime is simply one where neural activity remains confined to a lower-dimensional subspace, but under what circumstances would downstream readout neurons have explicit access to the low dimensional structure?

This comment was largely addressed in earlier responses (see modified text in lines 183+).

Minor comments:

- In Fig 6C, the authors argue that the error increases as the dimensionality (k) is increased. It seems that the MSE consistently decreases again above k~40. Could the authors extend k to higher values? Is this being run with several seeds?

Yes this is an artefact that went away when taking an average over multiple random seeds.

- Could the authors expand on the main contributing factor here to drift in the empirical covariance structure? Is my understanding correct that drift is caused primarily by the change in readout weights D(t) through learning, which changes learning feedback input I_fb (t)? Does the noise term also potentially contribute?

We added (lines 212+):

In the present framework, drift in the covariance structure arises from the interaction between decoder adaptation and recurrent dynamics. Specifically, changes in decoder weights $D(t)$ alter the feedback input $I_{\mathrm{fb}}(t)$, which in turn modifies the trajectories of recurrent activity explored by the network. As learning progresses, these trajectories reshape the covariance structure of the network. Thus, even though the recurrent connectivity matrix $W$ remains fixed, the low-dimensional manifold can evolve over time. The stochastic noise term $\xi\left( t \right)$ contributes additional variability to neural activity but it is not the primary source of manifold drift in the present simulations.

- Does the drift noise \eta_r (listed in Table 1. Default model parameters) enter any relevant equations? Where is the drift noise term used?

We thank the reviewer for pointing out this ambiguity. In the original draft, the parameter η_r was implicitly absorbed into the stochastic activity term in Eq. 1, which made its role unclear. We have now revised Eq. 1 to make this dependence explicit by writing the noise term as η_rξ(t), where η_r controls the amplitude of stochastic perturbations to the recurrent dynamics. This change clarifies the relationship between the parameter listed in Table 1 and the stochastic perturbations used in the simulations.

- Would it make more sense to use y(t) or f(t) as the network output, instead of z(t)? I am more familiar with y(t) as output, but have no problem with the current notation if this tends to be the convention in this specific area.

y(t) is already used for manifold coordinates, so reusing it as the network output would create ambiguity. Notation f(t) is also commonly used for nonlinear activation functions, so keeping z(t) as the decoder output is cleaner.

- The authors consider here a reservoir computing setting, where the input weights and recurrent weights are fixed. It is however also possible for the input weights and recurrent weights to be learned through algorithms such as e-prop/RFLO. Would this framework also easily extend to such a scenario?

We thank the reviewer for this suggestion. The present work focuses on a reservoir-computing setting in order to isolate the role of online manifold tracking during supervised decoder adaptation. However, we agree that an important future direction would be to combine SPLiT with recurrent learning rules such as e-prop or RFLO. We added this (lines 859+):

Although the present study focused on decoder adaptation within a reservoir framework, the manifold-tracking component of SPLiT could in principle be combined with biologically-motivated learning rules for recurrent neural networks such as e-prop or RFLO [46,48], allowing plasticity within the network to be constrained by an evolving low-dimensional population structure.

References:

Bellec, G., Scherr, F., Subramoney, A., Hajek, E., Salaj, D., Legenstein, R., & Maass, W. (2020). A solution to the learning dilemma for recurrent networks of spiking neurons. *Nature communications*, *11*(1), 3625.

Murray, J.M., 2019. Local online learning in recurrent networks with random feedback. *Elife*, *8*, p.e43299.
