# Supplementary material for: Manifold-constrained plasticity enables stable learning in recurrent neural circuits
Source: PLoS Comput Biol. 2026 Aug 25;22(8):e1014719. doi: 10.1371/journal.pcbi.1014719 (PMC13529248; doi:10.1371/journal.pcbi.1014719)
Supplement: Peer review history

# Peer review: Decision Letter 0

**DOI:** 10.1371/journal.pcbi.1014719.r001

**Authors:** Andrea E. Martin, Timothée Proix

8 May 2026

PCOMPBIOL-D-26-00458

Manifold-Constrained Plasticity Enables Stable Learning in Recurrent Neural Circuits

PLOS Computational Biology

Dear Dr. Thivierge,

Thank you for submitting your manuscript to PLOS Computational Biology. After careful consideration, we feel that it has merit but does not fully meet PLOS Computational Biology's publication criteria as it currently stands. Therefore, we invite you to submit a revised version of the manuscript that addresses the points raised during the review process.

Please submit your revised manuscript by Jul 08 2026 11:59PM. If you will need more time than this to complete your revisions, please reply to this message or contact the journal office at ploscompbiol@plos.org. When you're ready to submit your revision, log on to https://www.editorialmanager.com/pcompbiol/ and select the 'Submissions Needing Revision' folder to locate your manuscript file.

Please include the following items when submitting your revised manuscript:

* A letter that responds to each point raised by the editor and reviewer(s). You should upload this letter as a separate file labeled 'Response to Reviewers'. This file does not need to include responses to formatting updates and technical items listed in the 'Journal Requirements' section below.

* A marked-up copy of your manuscript that highlights changes made to the original version. You should upload this as a separate file labeled 'Revised Manuscript with Track Changes'.

* An unmarked version of your revised paper without tracked changes. You should upload this as a separate file labeled 'Manuscript'.

If you would like to make changes to your financial disclosure, competing interests statement, or data availability statement, please make these updates within the submission form at the time of resubmission. Guidelines for resubmitting your figure files are available below the reviewer comments at the end of this letter

We look forward to receiving your revised manuscript.

Kind regards,

Timothée Proix

Academic Editor

PLOS Computational Biology

Andrea E. Martin

Section Editor

PLOS Computational Biology

**Journal Requirements:**

1) We ask that a manuscript source file is provided at Revision. Please upload your manuscript file as a .doc, .docx, .rtf or .tex. If you are providing a .tex file, please upload it under the item type u2018LaTeX Source Fileu2019 and leave your .pdf version as the item type u2018Manuscriptu2019.

2) <carina-action-element class="ng-star-inserted">Please upload all main figures as separate Figure files in .tif or .eps format. For more information about how to convert and format your figure files please see our guidelines: </carina-action-element>

<carina-action-element class="ng-star-inserted">https://journals.plos.org/</carina-action-element><carina-action-element class="ng-star-inserted">ploscompbiol</carina-action-element><carina-action-element class="ng-star-inserted">/s/figures</carina-action-element>

3) We have noticed that you have uploaded Supporting Information files, but you have not included a complete list of legends. Please add a full list of legends for your Supporting Information files after the references list.

**Reviewers' comments:**

Reviewer's Responses to Questions

**Comments to the Authors:**

**Please note here if the review is uploaded as an attachment.**

Reviewer #1: Review of: Manifold-Constrained Plasticity Enables Stable Learning in Recurrent Neural Circuits

This manuscript introduces a learning rule for reservoir networks that separates two distinct processes: tracking changes of a low-dimensional activity manifold and learning on that drifting manifold. The approach is conceptually interesting and the results appear promising. I believe this is valuable work; however, there are several points that would benefit from clarification before publication. My comments fall into three categories: conceptual issues that may require additional analyses, presentation issues that currently make the manuscript difficult to follow, and minor points.

Conceptual issues

- Manifold rotation as the dominant form of drift. A growing body of evidence suggests that the dominant form of representational drift in neural circuits is a rotation of the population activity manifold, rather than a remapping that keeps activity confined to the same manifold (e.g. Sylte et al. 2025; recent review by Eppler et al. 2025, and references therein; see also Ziv et al. 2013, where this effect is visible in the figures although not emphasized). During drift, a substantial fraction of neurons drop in and out of the active population, which appears more consistent with a rotation of the entire manifold than with activity remaining on a fixed one. This is precisely the regime in which, based on the results presented, the proposed algorithm seems to perform least well. I would therefore encourage the authors to include an analysis in which drift is implemented as a sequence of small, incremental rotations, and to benchmark SPLiT against FORCE and the other algorithms compared in Figure 8. How does performance degrade (or not) as the number and magnitude of rotations vary?

- Task generality. The temporal tracking task used as the primary benchmark is relatively abstract. It would be helpful to see the reaching task evaluated more prominently. In particular, I encourage the authors to include a comparison analogous to Figure 8 using the reaching task instead of the temporal tracking task. Ideally, this could also be evaluated on a rotated manifold, to assess performance under a biologically plausible form of drift with a non-stationary target manifold.

- Normalization and gain. The manuscript states that the network is normalized to the largest eigenvalue, while also treating gain as a free parameter. As written, I found this difficult to reconcile: if the gain is modified and the network is subsequently renormalized to a largest eigenvalue of 1, the gain change would appear to have no lasting effect. I would appreciate clarification of the order of operations and why both procedures are necessary, or alternatively a simplification of the parameterization to ensure internal consistency.

Presentation issues

- Description of the method in the Results. The manuscript introduces SPLiT in the Results section but would benefit from a more intuitive or qualitative description of the method at that stage. In particular, it would help to briefly outline what the rule does, which variables it modifies, and what behavior it is expected to induce in the network. As it stands, it is difficult to interpret the reported results or follow the logic of the early figures without repeatedly consulting the Methods. I recommend including a concise, high-level description of the learning rule in the Results section, covering at minimum the type of network used, the mechanism of SPLiT, how training proceeds, and how dimensionality is constrained, before presenting results. A similar point applies to the handwritten digit task: a brief description in the main text before or alongside the results would substantially improve readability.

- Motivation for FORCE's behavior at low dimensionality. On p. 3 (l. 82) and p. 4 (l. 141), the text alludes to a specific reason why FORCE performs poorly in low-dimensional settings, but the explanation is deferred to the Discussion. Providing at least some intuition in the Results section would help the reader follow the narrative without needing to skip ahead.

Minor points

- Discussion of drift mechanisms. Recent theoretical work suggests that representational drift may in part reflect ongoing learning or synaptic plasticity (Qin et al. 2023; Ratzon et al. 2024; Devalle et al. 2025; Eppler et al. 2026). The Discussion would benefit from engaging more directly with this literature. In particular, how does ongoing learning interact with manifold stability at the representational level? Does the network output also drift, and if so, is ongoing plasticity in the readout population the relevant mechanism?

- Principal angle metric (p. 4, l. 135; Fig. 2). It would be helpful to clarify whether a mean principal angle near zero is a meaningful outcome under this definition, or whether random, uncorrelated angles would also average to approximately zero. If the latter, the metric may not distinguish dynamics in the intended way.

- Figure 2. The left (panels A-C) and right (panels D-F) parts could be labeled explicitly as “constrained” and “unconstrained” (e.g., as titles or annotations) to make the comparison immediately clear.

- Figure 4. A legend should be added to the MNIST tracking panel, as it is currently unclear what the colors represent.

- Use of λ. The symbol λ is used both for the learning rate and for eigenvalues. Distinct notation would improve clarity.

- Figures 5, 6, and 7. These figures compare the constrained and unconstrained versions of SPLiT but do not include FORCE. As FORCE is a standard baseline, including it would strengthen the comparison.

- Figure 6A. A colorbar appears to be missing and should be added.

- Figure 6C. At certain dimensionalities, the MSE decreases again after an initial rise. This non-monotonic pattern is unexpected and currently unexplained. It would be helpful to either discuss the underlying mechanism or clarify whether this might reflect an artifact.

- Figure 7A. A legend is missing and should be added.

- Methods: initialization of W. The initialization procedure is described in a way that seems more complex than necessary. If lower-rank matrices cannot be generated directly, it would be helpful to briefly explain why the current approach is required.

Reviewer #2: Uploaded as attachment.

Reviewer #3: Review uploaded as an attachment.

**********

**Have the authors made all data and (if applicable) computational code underlying the findings in their manuscript fully available?**

The PLOS Data policy requires authors to make all data and code underlying the findings described in their manuscript fully available without restriction, with rare exception (please refer to the Data Availability Statement in the manuscript PDF file). The data and code should be provided as part of the manuscript or its supporting information, or deposited to a public repository. For example, in addition to summary statistics, the data points behind means, medians and variance measures should be available. If there are restrictions on publicly sharing data or code —e.g. participant privacy or use of data from a third party—those must be specified.

Reviewer #1: Yes

Reviewer #2: Yes

Reviewer #3: Yes

**********

PLOS authors have the option to publish the peer review history of their article (what does this mean?). If published, this will include your full peer review and any attached files.

If you choose “no”, your identity will remain anonymous but your review may still be made public.

**Do you want your identity to be public for this peer review?**  For information about this choice, including consent withdrawal, please see our Privacy Policy.

Reviewer #1: **Yes:** Jens-Bastian Eppler

Reviewer #2: No

Reviewer #3: No

[NOTE: If reviewer comments were submitted as an attachment file, they will be attached to this email and accessible via the submission site. Please log into your account, locate the manuscript record, and check for the action link "View Attachments". If this link does not appear, there are no attachment files.]

**Figure resubmission:**

While revising your submission, we strongly recommend that you use PLOS’s NAAS tool (https://ngplosjournals.pagemajik.ai/artanalysis) to test your figure files. NAAS can convert your figure files to the TIFF file type and meet basic requirements (such as print size, resolution), or provide you with a report on issues that do not meet our requirements and that NAAS cannot fix.

After uploading your figures to PLOS’s NAAS tool - https://ngplosjournals.pagemajik.ai/artanalysis, NAAS will process the files provided and display the results in the "Uploaded Files" section of the page as the processing is complete. If the uploaded figures meet our requirements (or NAAS is able to fix the files to meet our requirements), the figure will be marked as "fixed" above. If NAAS is unable to fix the files, a red "failed" label will appear above. When NAAS has confirmed that the figure files meet our requirements, please download the file via the download option, and include these NAAS processed figure files when submitting your revised manuscript.

**Reproducibility:**

To enhance the reproducibility of your results, we recommend that authors of applicable studies deposit laboratory protocols in protocols.io, where a protocol can be assigned its own identifier (DOI) such that it can be cited independently in the future. Additionally, PLOS ONE offers an option to publish peer-reviewed clinical study protocols. Read more information on sharing protocols at https://plos.org/protocols?utm_medium=editorial-email&utm_source=authorletters&utm_campaign=protocols

Attachment Submitted filename: 20260414 PLoS Review PCOMPBIOL-D-26-00458.md

Attachment Submitted filename: Reviewer Report.docx

# Author comment: Author response to Decision Letter 1

**DOI:** 10.1371/journal.pcbi.1014719.r002

15 Jun 2026

Attachment Submitted filename: PLoS reply to reviewers r2 v2.docx

# Peer review: Decision Letter 1

**DOI:** 10.1371/journal.pcbi.1014719.r003

**Authors:** Andrea E. Martin, Timothée Proix

17 Aug 2026

Dear Dr. Thivierge,

We are pleased to inform you that your manuscript 'Manifold-Constrained Plasticity Enables Stable Learning in Recurrent Neural Circuits' has been provisionally accepted for publication in PLOS Computational Biology.

Before your manuscript can be formally accepted you will need to complete some formatting changes, which you will receive in a follow up email. A member of our team will be in touch with a set of requests.

Please note that your manuscript will not be scheduled for publication until you have made the required changes, so a swift response is appreciated.

IMPORTANT: The editorial review process is now complete. PLOS will only permit corrections to spelling, formatting or significant scientific errors from this point onwards. Requests for major changes, or any which affect the scientific understanding of your work, will cause delays to the publication date of your manuscript.

Should you, your institution's press office or the journal office choose to press release your paper, you will automatically be opted out of early publication. We ask that you notify us now if you or your institution is planning to press release the article. All press must be co-ordinated with PLOS.

Thank you again for supporting Open Access publishing; we are looking forward to publishing your work in PLOS Computational Biology.

Best regards,

Timothée Proix

Academic Editor

PLOS Computational Biology

Andrea E. Martin

Section Editor

PLOS Computational Biology

***********************************************************

Reviewer's Responses to Questions

**Comments to the Authors:**

**Please note here if the review is uploaded as an attachment.**

Reviewer #1: I thank the authors for their careful revisions and for addressing my comments. I am satisfied with the changes and believe that the manuscript has been substantially improved. I enjoyed reading the paper and support its publication.

Reviewer #2: Dear Authors,

I have gone over your revisions and feel they address my questions and concerns well enough to merit publication. Thank you for this: drilling through the math was quite challenging but a rewarding study in the end. Many unanswered questions remain, and I look forward to those questions being explored in follow up studies building upon your work (:

Reviewer #3: I thank the authors for their careful responses and revisions. In particular, moving part of the Methods section to the beginning of the Results has greatly improved the manuscript's readability. My previous comments have been satisfactorily addressed, and I have no further concerns regarding the manuscript.

**********

**Have the authors made all data and (if applicable) computational code underlying the findings in their manuscript fully available?**

The PLOS Data policy requires authors to make all data and code underlying the findings described in their manuscript fully available without restriction, with rare exception (please refer to the Data Availability Statement in the manuscript PDF file). The data and code should be provided as part of the manuscript or its supporting information, or deposited to a public repository. For example, in addition to summary statistics, the data points behind means, medians and variance measures should be available. If there are restrictions on publicly sharing data or code —e.g. participant privacy or use of data from a third party—those must be specified.

Reviewer #1: None

Reviewer #2: Yes

Reviewer #3: None

**********

PLOS authors have the option to publish the peer review history of their article (what does this mean?). If published, this will include your full peer review and any attached files.

If you choose “no”, your identity will remain anonymous but your review may still be made public.

**Do you want your identity to be public for this peer review?**  For information about this choice, including consent withdrawal, please see our Privacy Policy.

Reviewer #1: **Yes:** Jens-Bastian Eppler

Reviewer #2: No

Reviewer #3: No

# Editor report: Acceptance letter

**DOI:** 10.1371/journal.pcbi.1014719.r004

**Authors:** Andrea E. Martin, Timothée Proix

PCOMPBIOL-D-26-00458R1

Manifold-Constrained Plasticity Enables Stable Learning in Recurrent Neural Circuits

Dear Dr Thivierge,

I am pleased to inform you that your manuscript has been formally accepted for publication in PLOS Computational Biology. Your manuscript is now with our production department and you will be notified of the publication date in due course.

The corresponding author will soon be receiving a typeset proof for review, to ensure errors have not been introduced during production. Please review the PDF proof of your manuscript carefully, as this is the last chance to correct any errors. Please note that major changes, or those which affect the scientific understanding of the work, will likely cause delays to the publication date of your manuscript.

Soon after your final files are uploaded, unless you have opted out, the early version of your manuscript will be published online. The date of the early version will be your article's publication date. The final article will be published to the same URL, and all versions of the paper will be accessible to readers.

For Research, Software, and Methods articles, you will receive an invoice from PLOS for your publication fee after your manuscript has reached the completed accept phase. If you receive an email requesting payment before acceptance or for any other service, this may be a phishing scheme. Learn how to identify phishing emails and protect your accounts at https://explore.plos.org/phishing.

Thank you again for supporting PLOS Computational Biology and open-access publishing. We are looking forward to publishing your work!

With kind regards,

Sharmila Kamatchi

PLOS Computational Biology | Carlyle House, Carlyle Road, Cambridge CB4 3DN | United Kingdom ploscompbiol@plos.org | Phone +44 (0) 1223-442824 | ploscompbiol.org | @PLOSCompBiol
